# Profile of Polyphenolic and Essential Oil Composition of Polish Propolis, Black Poplar and Aspens Buds

**DOI:** 10.3390/molecules23061262

**Published:** 2018-05-25

**Authors:** Piotr Okińczyc, Antoni Szumny, Jakub Szperlik, Anna Kulma, Roman Franiczek, Beata Żbikowska, Barbara Krzyżanowska, Zbigniew Sroka

**Affiliations:** 1 Department of Pharmacognosy, Wrocław Medical University, 50556 Wrocław, Poland; beata.zbikowska@umed.wroc.pl (B.Ż.); zbigniew.sroka@umed.wroc.pl (Z.S.); 2 Department of Chemistry, Wrocław University of Environmental and Life Sciences, 50375 Wrocław, Poland; antjasz@o2.pl; 3 Department of Biotechnology, Wrocław University, 51148 Wrocław, Poland; anna.kulma@uwr.edu.pl; 4 Department of Microbiology, Wrocław Medical University, 50368 Wrocław, Poland; roman.franiczek@umed.wroc.pl (R.F.); barbara.krzyzanowska@umed.wroc.pl (B.K.)

**Keywords:** bee glue, poplars, principal component analysis (PCA), essential oils (EOs), bee glue’s polyphenols

## Abstract

In this work, we studied similarities and differences between 70% ethanol in water extract (70EE) and essential oils (EOs) obtained from propolis, black poplars (*Populus nigra* L.) and aspens (*P. tremula* L.) to ascertain which of these is a better indicator of the plant species used by bees to collect propolis precursors. Composition of 70EE was analyzed by UPLC-PDA-MS, while GC-MS was used to research the EOs. Principal component analyses (PCA) and calculations of Spearman’s coefficient rank were used for statistical analysis. Statistical analysis exhibited correlation between chemical compositions of propolis and *Populus* buds’ 70EE. In the case of EOs, results were less clear. Compositions of black poplars, aspens EOs and propolises have shown more variability than 70EE. Different factors such as higher instability of EOs compared to 70EE, different degradation pattern of benzyl esters to benzoic acid, differences in plant metabolism and bees’ preferences may be responsible for these phenomena. Our research has therefore shown that 70EE of propolis reflected the composition of *P. nigra* or complex aspen–black poplar origin.

## 1. Introduction

Propolis (bee glue) is a viscous, aromatic substance produced by different species of bees, the most commercially available being the one produced by honey bee (*Apis mellifera* L.). Bee glue is a mixture of plant exudates, bees wax and secretions of bees’ glands. Propolis is used by bees to insulate hive, repair any damage to hive structure and for hygienic purposes. The biological activities of bee glue are widely and well documented in the literature, including its medicinal properties such as antimicrobial, anti-inflammatory, antioxidative and wound-healing [1,2]. All of the biologically active components of propolis originate from plant exudates and resins, which are called plant precursors. Therefore, the species of those plants play a pivotal role in the properties of the final product [1,2].

Although bees are capable of foraging from a variety of plant sources, they show preference and prioritize certain species of plants, depending on a region. Therefore, composition of propolis is not completely random and it could be divided into several chemical types, based on main plant precursors [1,2,3,4].

In temperate climatic zone, exudates from buds of black poplar (*Populus nigra* L.) are predominantly collected [3,4]. However, it was shown that other *Populus* species could also be propolis precursors [3,4,5,6,7], among them *P. tremula* L. (aspen). It is significant that composition of aspens’ exudates is different from black poplars resins. [3,4,5,6,7]. The presence of phenolic acids glycerides in aspen buds and aspen propolis constitutes the main difference [3,4,5,6,7]. These components are absent in *P. nigra* L. buds exudates [3,4,5,6,7] which contain their own specific markers such as flavonoids (e.g., chrysin). Other mixed types of propolis such as aspen–poplar [3,4,5] and aspen–birch–poplar [6] are also known.

Composition of propolis and its plant precursors are not fully known, which is especially apparent in the area of comparative investigation of bee glue and its plant sources. Available data for comparative analysis between bee glue and *Populus* from temperate climate include research performed using different techniques such as TLC (thin layer chromatography), TLC-MS (thin layer chromatography coupled with mass spectrometry) [5], GC-MS (gas chromatography coupled with mass spectrometry) after sililation [3,4,6,7], and UHPLC-MS/MS (ultra-high performance liquid chromatography coupled with tandem mass spectrometry) [8,9]. However, comparative analysis of volatile components remains under-researched [2]. Moreover, not all aspects of the composition of *Populus* genus essential oils (EOs) have been studied. According to Jerković and Mastelić [10], *P. nigra* L. leaf buds contain mainly oxygenated sesquiterpenoids. As far as we know, the composition of volatile compounds of aspen and other *Populus* buds is still not properly researched. Available information on *P. tremula* L. essential oils includes only emission of terpenoids from leaves [11] and bark [12].

It is also noteworthy that propolis composition is unstable during vegetation season with reference to the data of polyphenolic extract of tropical samples (Brazilian green [13], Brazilian red [14], and Sonoran [15]) and research from south Portugal [16]. Similar investigation has not been performed for aspen–poplar propolis. 

Therefore, as the biological activities of propolis are related to the concentration of polyphenols and volatile components, observation of their profiles may be useful in future investigations, for example in the search of cheaper or more easily available propolis replacements. Poplar is a known source of various folk remedies, effectiveness of which has sometimes been documented. Over time, such remedies fell into disuse and mostly replaced by propolis. Nevertheless, poplar derivates still could be used as food additives. As propolis is known for instability of both its composition and biological activity, usually with no clear cause and effect correlation between the two, *Populus* extracts could be a favorable replacement. Such a solution could even be economically viable considering the low cost of propagation and fast growth of the trees.

Due to a greater abundance and a generally higher stability of non-volatile components in propolis and *Populus* spp. exudates 70% ethanol in water extracts (70EE), we present a hypothesis that 70EE are better for identifying propolis plant precursors compared to essential oils (EOs).

## 2. Results

### 2.1. Composition of Essential Oils of Propolis and Its Plant Precursor

During preliminary research, hydrodistillation–extraction (HDE) using a Deryng apparatus and SDE (simultaneous distillation–extraction to dichloromethane) was performed to obtain volatile propolis derivates. Unlike HDE, SDE did not show any problems with clogging by waxy residue.

The presence of 279 components (29% were tentatively identified and 71% were fully identified) was exhibited by GC-MS analysis. Only the components that exceeded at least 10% in single sample of those shown in Table 1 and Table 2 were used in statistical analyses. Full results are attached as Supplementary Materials (Tables S1–S3). General amounts of compounds according to chemical classes are shown in Table 3 and Table 4.

Identified components were assigned to several groups (see Table 3) and samples were characterized by the amounts of several component types. The highest amount of investigated essential oils was observed in black poplar sample: PN-3 (4.33%). The remaining *P. nigra* samples exhibited a range of 0.50% to 1.50% of EOs. *P. tremula* EOs’ content varied from trace amount (<0.05%) to 0.36%. Propolis contained 0.07% to 2.8% of EOs.

Black poplars buds exhibited different EOs profile, differing in both quality and quantity. Some buds contained mainly oxygenated sesquiterpenoids (83% in PN3 and 86% in PN5; large amount of α-, β- and γ-eudesmols), while two other samples were mainly composed of sesquiterpene hydrocarbons (73% in PN1 and 66% in PN2; main components were *ar*- and γ-curcumens and δ-cadinen). PN4, PN6 and PN7 showed a different profile, containing a mix of sesquiterpenes and sesquiterpenoids (45% in PN4, 32% in PN6, and 40% in PN7) and derivates of benzoic acid (49% in PN4, 54% in PN6 and 48% in PN7, mainly prenyl benzoate).

Aspen buds also exhibited different profiles of EOs. Four aspens contained mostly benzoic acid derivates (69% in PT4, 55% in PT1 and 51% in PT5). In PT1, benzyl benzoate was the main component (45.45%), while composition of PT4 and PT5 was more complex. Another pattern of composition was observed in PT2 and PT3, which contained mainly sesquiterpene derivates (91% in PT2 and 92% in PT3). EOs of PT2 were composed of similar amount of sesquiterpene hydrocarbons (*cis*-β-cariophyllene and α-guaiene) and oxygenated sesquiterpenoids (cariophyllene oxide), while PT3 contained higher amount of oxygenated sesquiterpenoids than sesquiterpenes. In the case of the last sample, PT6, a different type of EOs profile was observed:a mixture of 2-phenylethanol, unidentified aliphatic component, benzyl alcohol and eugenol.

Propolis’ EOs were usually composed of benzoic acid derivates (mainly benzoic acid and benzyl benzoate) and a mixture of oxygenated sesquiterpenoids (typical components were α-, β-, and γ-eudesmols). Only two samples (PR-LS6 and PR-GR) contained low amount of benzoic acid derivates. Moreover, in PR-GR, concentration of sesquiterpenes hydrocarbons (mainly *ar*- and γ-curcumens) was higher than oxygenated sesquiterpenoids.

### 2.2. Identification of Compounds Present in 70EE and UPLC-PDA-MS Profile of Propolis and Populus Buds

Results of polyphenols analysis are shown in Table 5 and Table 6. Complete results of the UPLC-PDA-MS (ultra-performance liquid chromatography coupled with photodiode array and mass spectrometry) analysis with identification data are attached as Supplementary Materials (Tables S4–S7). Seventy-seven components were identified in all samples, most of which were identified based on literature data (see supplement Table S4), according to the characteristic UV (ultraviolet light) absorption spectrum and mass fragmentation in negative ionization mode. In the case of most flavonoids, free phenolic acids and their monoesters, exhaustive literature data allow certain extent of identification. Phenolic acids glycerides proved difficult to identify, especially prediction of glycerol substitution position to phenolic acids due to a scarcity of HPLC-MS ion fragmentation literature data and a lack of available standards.

The profiles of 70% ethanol in water extract have shown certain variation between the samples. Propolis, aspens and black poplars exhibited qualitative and quantitative differences within their groups. In the case of *P. nigra*, four samples (PN1, PN2, PN3 and PN5) contained mainly flavonoid aglycones (chrysin, pinocembrin chalcone, galangin, pinocembrin-3-*O*-acetate and pinostrobin chalcone) and lower amount of free phenolic acid (*p*-coumaric acid) and their monoesters (benzyl and cinnamyl esters of *p*-coumaric acid). Different composition was observed for three *P. nigra* buds (PN4, PN6 and PN7), which contained a higher amount of free phenolic acids and their monoesters, but contained less flavonoid aglycones than PN4, PN6 and PN7.

Unlike black poplars, 70EE of aspens contained mainly free phenolic acids, phenolic acid glycerides (especially 2-acetyl-1,3-di-*p*-coumaroylglycerol and some phenolic acid monoesters (for example, *p*-coumaric acid benzyl ester). It is worth noting, however, that profiles of aspens were more uniform than those of black poplars.

Propolis samples had a composition resembling a mix of substances characteristic for aspens (phenolic acid glycerides) or poplar (flavonoids) (18 samples) or exhibited a profile similar to *P. nigra* (three samples: Polish (PR-LS6), German (PR-GR) and Canadian propolis (PR-CAN)).

### 2.3. Statistical Analysis of 70% Ethanol in Water Extracts and Essential Oils

Principal component analysis of 70EE has shown that a nine-principal-component model explained 99.3% of total variance, while 99.2% of total variance of EOs PCA analysis required a thirteen-principal-component model. Models limited to only two most important principal components explained 75.4% of 70EE total variance and 58.5% of EOs total variance. When poplars and aspens were excluded from analysis, the explanations of total variance were higher for two-component models (91.5% for 70EE and 79.2% for EOs).

**Figure 1:**
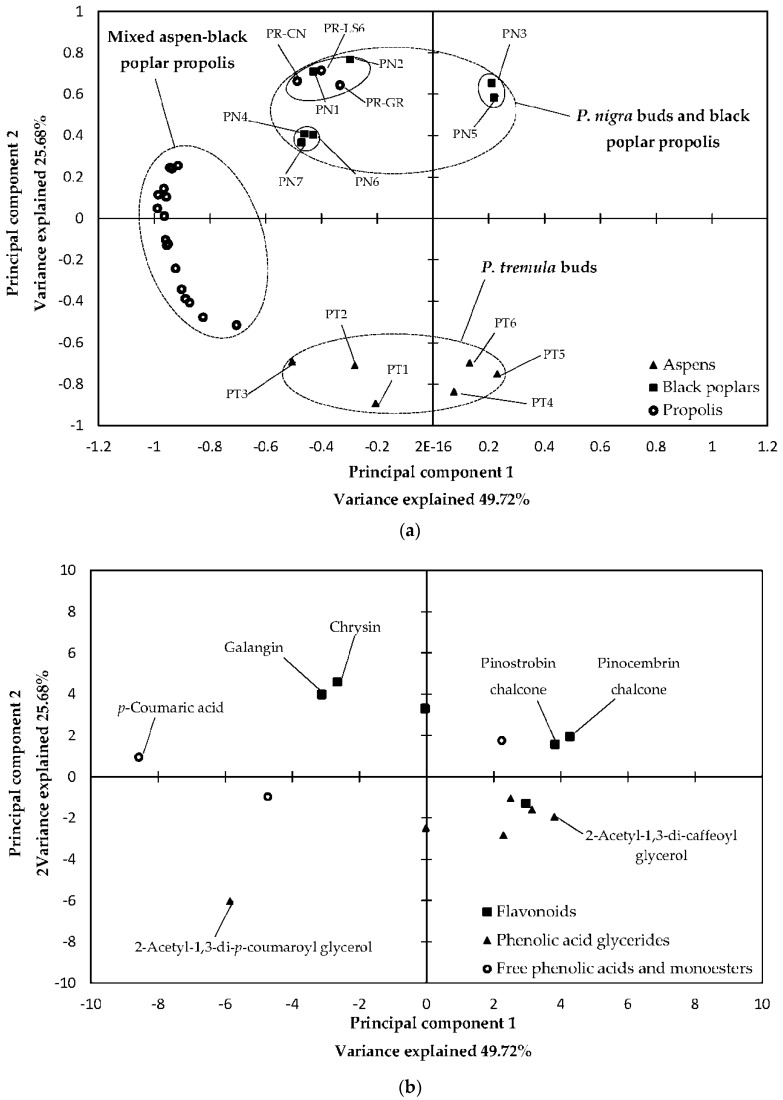
PCA of 70% ethanol in water extracts analysis: score plot (**a**); and load plot (**b**).

**Figure 2:**
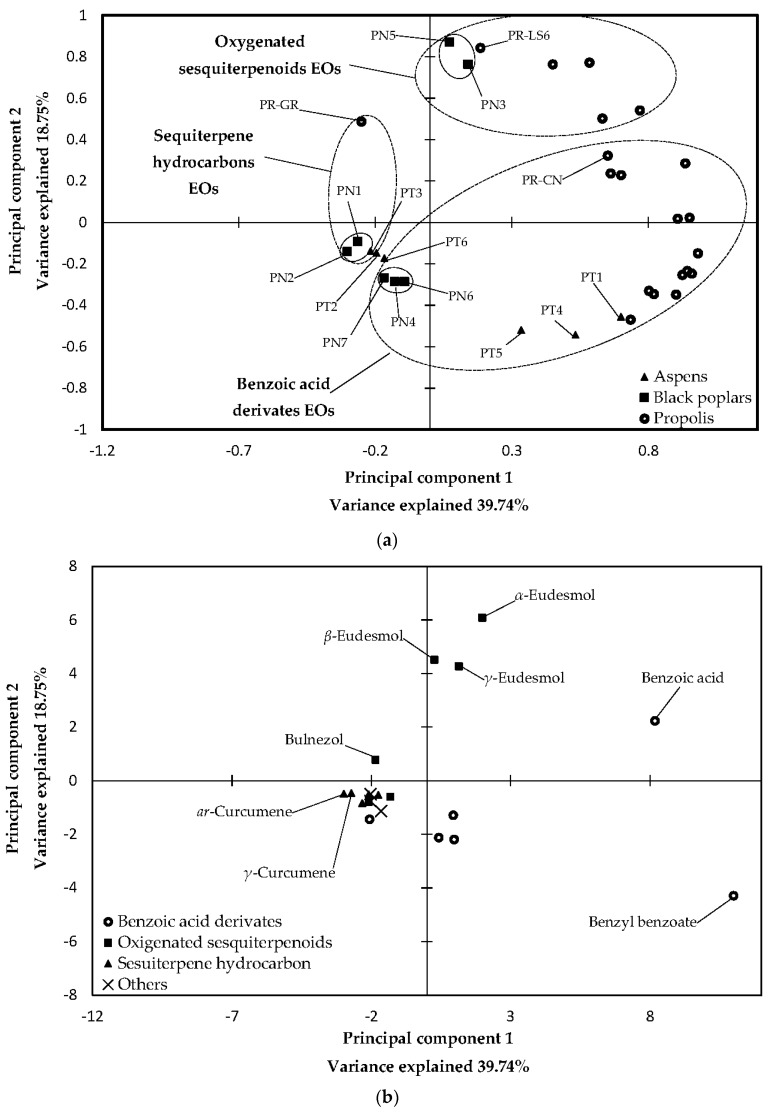
PCA of essential oils: score plot (**a**); and load plot (**b**).

Graphical results of PCA analysis (two-principal-component models: factor scores and their loading) are presented in Figure 1 (PCA of 70EE) and Figure 2 (PCA of EOs).

In PCA of 70EE, the first principal component was composed mainly of *p*-coumaric acid, 2-acetyl-1,3-di-*p*-coumaroylglycerol, p-coumaric acid benzyl ester, pinocembrin chalcone and pinostrobin chalcone. 2-acetyl-1,3-di-*p*-coumaroylglycerol, galangin, chrysin, pinobanksin-3-*O*-acetate and 2-acetyl-1-caffeoyl-3-di-*p*-coumaroylglycerol exhibited the highest effect on the second principal component of 70EE, with the strength of their impact decreasing in the order they were mentioned. For the EOs analyze, benzyl benzoate, benzoic acid, *ar*- and γ-curcumens and cis-β-caryophyllene were the main components of the first principal component. The second principal component of EOS was composed of α- and β-eudesmols, benzyl benzoate, γ-eudesmol and benzoic acid.

As shown in Figure 1, 70EE samples may be divided in several groups (*P. nigra* buds and black poplar propolis, mixed aspen–black poplar propolis and *P. tremula* buds). It is significant that propolis samples were separated according to the plant origin (black poplar propolis or mixed aspen–poplar bee glue). Conversely, for EOs, propolis samples could not be divided according to plant origins and were separated according to their main chemical components. Moreover, some black poplars and aspens samples were close to each other. In PCA of EOs, black poplars samples reflect similar distribution to each other such as in PCA of 70EE (PN1 and PN2 created one sub-cluster; PN3 and PN5 created the second sub-cluster; and PN4, PN6 and PN7 created the third sub-cluster). In the case of propolis and aspen samples, their own clusters were not preserved in PCA of EOs (see Figure 1 and Figure 2 and compare the distribution of black poplars, aspens and propolis samples).

Analysis of Spearman’s coefficient rank exhibited more similarities between 70EEs compositions than EOs. More positive correlations between propolis and *Populus* buds were observed for 70EEs (42) than EOs (35). Numbers of positive correlations between same propolis samples (propolis vs. propolis) were 300 for 70EEs and 246 for EOs.

Moreover, propolis EOs were more often observed to exhibit positive correlation with *Populus* buds that were not their plant precursor than in 70EE analysis. The most outstanding result was observed for PR-LS2 bee glue. The 70EE of this sample was correlated with black poplar (PN1), but the EOs with aspens (PT1 and PT4).

## 3. Discussion

### 3.1. The Composition of Populus spp. and Propolis EOs

The average amount of EOs in worldwide propolis is about 0.5% [2], which was reflected by the analyzed samples. For black poplars, *P. nigra* buds contained 0.27% (fresh buds) and 0.12% (dried buds) EOs [10]. The majority of analyzed samples followed a similar pattern, except for PN3, where it reached 4.3%. It may have been due to the highest apparent proportional content of resins in those samples. However, detailed investigation of resin content was beyond the scope of this work. For aspen’s buds, similar information could not be found.

In the literature, only varieties of profiles characterized by high content of β-eudesmol (19.6%) and lower of α-eudesmol (6.0%) in dried *P. nigra* leaf buds were published [10]. Our results differed, as there were changes both to the general composition as well as to which of the compounds was dominant among them. Contrary to the literature data, the dominant compounds in the analyzed samples were sesquiterpene hydrocarbons, including *ar*- and γ-curcumens, and δ-cadinen, and derivates of benzoic acid. It may be speculated it was due to regional dominance of different chemotypes and thus different substances that were collected by the bees. A similar phenomenon occurred in the case of the aspens, although this warrants further research.

It is noteworthy that both aspen and black poplar buds contained no or trace amounts of benzoic acid. In our opinion, high amount of this component in propolis may be the result of degradation of some volatile and non-volatile benzyl derivates. Some data [17,18,19,20,21] suggest that bees preferentially collect exudates from vegetative poplar buds which are rich in benzyl derivates (as they inhibit *Populus* buds cracking and maturation into leaves). Benzoic acid is a known food preservative and it may be possible that bees in the northern hemisphere have a preference towards exudates degradation which results in the presence of this component in propolis.

However, it has been shown [2] that propolis EOs may contain high concentration of other compounds characteristic of *P. nigra* (for example, δ-cadinen in Albanian propolis). In our samples, PR-GR contained high amount of *ar*- and γ-curcumens, which were main components of two black poplar samples. It is worth noting that EOs components exhibited their own biological activity such as antibacterial [2,18] and acaricidal [19] and some of those components may work as a primary or secondary attractant or on contrary a repellent, for honey bees.

### 3.2. The Composition of Populus spp. and Propolis 70EE

The results obtained are in agreement with earlier publications [3,4,5,8,9,20,21] showing GC-MS analysis of black poplar buds exudates, which were found to contain free phenolic acids, flavonoids and monoesters of phenolic acids and flavonoids. Unlike black poplars, 70EE of aspens contained free phenolic acids, phenolic acid glycerides (especially 2-acetyl-1,3-di-*p*-coumaroylglycerol and phenolic acid monoesters (for example, *p*-coumaric acid benzyl ester). Some positions of glycerol groups substituting their given compounds were tentatively identified according to GC-MS literature data of propolis and aspen buds analysis [3,4,22]. Only some position isomers were shown to be present in propolis in those works. For instance, only 2-acetyl-1,3-di-*p*-coumaroylglycerol was observed, while 1-acetyl-2,3-di-*p*-coumaroylglycerol was not found. Similarly, in the case of aspen buds, 2-acetyl-1,3-di-*p*-coumaroylglycerol was shown to be the dominant isomer [22]. It is therefore highly probable that isomer present in our sample was 2-acetyl-1,3-di-*p*-coumaroylglycerol.

Previous HPLC research [23] exhibited presence of salicylate-like components such as salicin and others in *P. nigra* buds, however salicin is absent in black poplar propolis. In our work, PDA peak of this substance (UVλ_max_ = 210, 260–265) was not observed for black poplar bud extracts, which is in full agreement with the above-mentioned author. However, some lower concentration ions in some peaks show similarity to salicin (M/Z for ESI-NEG = 285.1) and other salicylate-like compounds. This result was probably due to low concentration of salicin-like substances in poplar buds, extraction procedure and HPLC division condition [23,24]. It is also noteworthy that salicylic compounds characteristic for aspens (e.g., tremuloidin or tremulacin) were not observed [25]. Nevertheless, the presence of some higher mass ions (accompanying others peaks) in ESI-NEG mode may suggest their presence in samples, albeit at trace levels (such as in *P. nigra* buds case). Low concentration of salicylate-like substances in aspen buds is probably related to specific composition [3,4,22] and protective function of bud exudates. In our opinion, these components were a contamination from green tissues of the buds, but not the exudates themselves. Their presence in samples was likely caused by our extraction methods and not their function in exudates.

Propolis samples had a composition resembling a mixture of substances characteristic of aspen (phenolic acid glycerides) or poplar (flavonoids) (18 samples) or exhibited profile similar to *P. nigra* (three samples: Polish, German and Canadian propolis). The most common black poplar components in propolis include flavonoids chrysine, pinocembrine and pinobanksin-3-*O*-acetate [3,4,5,9]. Among the various phenolic acid glycerides, a higher concentration was observed for 2-acetyl-1,3-di-*p*-coumaroylglycerol. Some substances showed bigger peaks in propolis (*p*-coumaric acid benzyl ester) and some in its plant sources (pinostrobin chalcone). This apparent discrepancy in concentration could however be explained.

Firstly, bee enzymes (for example, cellulase, esterase, β-glucosidase [26], acidic pH and wet atmosphere of hive may lead to degradation of some components, especially compounds such as phenolic acid esters.

### 3.3. Statistical Analysis

Obtained results demonstrated that 70EE composition is more homogenous than EOS. It is worth noting, however, that samples of *P. nigra* sub clusters formed similar groupings for both 70EE and EOs analyzes. This is in contrast to the samples of aspens and propolises, where such behavior was not observed. It may indicate higher stability of essential oils from black poplar in comparison to those from aspens and propolises. It may be speculated it is so because *P. nigra* produces the exudates constantly throughout the whole vegetative season, while *P. tremula* produces exudates in any notable amounts only before the cracking of the buds [27]. These observations may also be a result of additional factors such as instability of essential oils during storage in hive, plant metabolism, spreading of different chemotypes of both *P. nigra* and *P. tremula* [28] and selection of plant material by bees. This also determined that non-volatile components (such as polyphenols) are a better choice to track propolis plant origin and may be used by bees as primary marker to select plant material before collecting [4,17]. However, the role of EOs as a secondary marker cannot be excluded due to an attractant role of some of the monoterpenes, sesquiterpenes and aromatic esters ([29]). Further research in this area is required to study their role.

It is significant that EOs did not contain any substance which may be used as strong marker of propolis origin. Specific markers were presented in propolis 70EE: phenolic acid glycerides for aspen and some flavonoids (galangin, chrysin, pinocembrin and its derivates) as *P. nigra* and *P. tremula* markers [3,4,5,9].

In preceding research, PCA analysis of polyphenols content and composition was used to investigate propolis samples of different origins such as Croatian [30], Portuguese [31], Serbian [30] (one work included also *P. nigra* sample [9]), Greek [32,33], German [34], and Turkish [32,35]. Statistical analyses based on EOs were less utilized, albeit performed (for example, in Brazil [36], China [37,38] and Estonia [39]). Chemometric analyses were also performed for *Salicaceae* family (including *Populus* genus) [40]. Ristivojević et al. [9] based PCA analysis of propolis and black poplars buds on quantified amounts of poliphenolic components. In their article, galangin, together with CAPE, chrysin and pinocembrin, were sufficient to classify samples of black poplar and of propolis types, albeit other types were not analyzed. In our opinion, it was possible because the abovementioned work was focused on only one *Populus* species and propolis that was the product of its exudates. As such, the samples were much more homogenous than the one investigated in our work, which makes their method valid for the particular task. Considering the diversity of chemical compositions of our samples, between black poplars and aspens, between their various chemotypes, and between those and various propolises collected by the bees from those sources, we felt such an approach would have been untenable for our work. Therefore, we tried applying UPLC-DAD chromatogram fingerprints. To avoid complications resulting from the impossibility to obtain standards of some of the compounds commercially, especially phenolic acid glycerides, peaks were calculated as a percentage of the whole chromatogram area, not as content in samples. In analyses of the 70% ethanol in water extract, we obtained a satisfactory discrimination of variables and two principal components model explained more than 70% of variability. Moreover, preliminary reduction analysis eliminated overlapping and unidentified peaks which may have disrupted the analysis. Considering this, we feel our techniques are tenable for our work.

Similar approach was also used for different GC-MS chromatograms. Isidorov et al. [3,4] successfully utilized it for hierarchical fuzzy clustering of different types of propolis.

In our work, we have shown for the first time the analysis of the similarities and differences between 70EE and EOs of Polish and other propolises, aspens and poplar buds. Simultaneous analyses of EOs and polyphenols were previously performed only for Brazilian samples [41].

## 4. Materials and Methods

### 4.1. Research Materials, Reagents and Standards

Twenty samples of propolis, seven of black poplars (PN1–7) and six of aspen (PT1–6) buds were analyzed in this work. Samples of bee glues were a mixture of material from different hives in the same apiary. These samples originated from Lower Silesia (12 samples: PR-LS1–6, PR-NW1–2, PR-ŚL1–2, PR-MR, and PR-NSW), West Pomerania (Szczecin, 4 samples: PR-SZ1–4), Podkarpacie (2 samples: PR-S1–2), Germany (commercial sample: PR-GR) and Canada (PR-CN).

*P. nigra* buds were collected in Szczodre (2013 and 2014), Wrocław (2015) (Lower Silesia, Poland) and Kórnik Arboretum of Institute of Dendrology of the Polish Academy of Sciences (2015) and bought from commercial sources. *P. tremula* buds were collected in Szczodre (2013, 2015 and 2016) and Arboretum Wojsławice, branch of Wrocław University Botanical Garden (2017). All of the samples were dried for three weeks prior to analysis.

Propolis was frozen in liquid nitrogen and mechanically ground in mortar. *Populus* buds were mechanically ground in an automatic mixer. Research materials were stored at −20 °C.

Ethanol and dichloromethane were purchased from POCH (Gliwice, Poland), Acetonitryl LC-MS from VWR Prolabo Chemicals (Leicestershire, UK) and 98% formic acid from Fluka (Buchs, Switzerland). Standards of *n*-alkanes (C_8_–C_23_) were obtained from Fluka (Buchs, Switzerland), those of polyphenols were purchased from Extrasynthese (Genay, France) which included acacetin, apigenin, caffeic acid, chrysin, ferulic acid, genkwanin, isoferulic acid, kempferol, and quercetin dihydrate. Pinobanksin was obtained from Sigma-Aldrich (Saint Louis, MO, USA).

### 4.2. Isolation and Analysis of Essential Oils (EOs)

Essential oils were obtained by hydro distillation–simultaneous extraction (SDE) according to Kujumgiev et al. [18]. Distillation time was set at 2 h (from original 4 h). After distillation, dichloromethane was evaporated and the amount of EOs was evaluated as percentage (%) of whole sample mass. The procedure was repeated twice.

Next, essential oils were analyzed by GC-MS in Varian chromatograph with mass spectrometer (GC-MS CP 3800 + Saturn 2000, Varian, Palo Alto, CA, USA) equipped with Zebron ZB-1MS, GC Capillary column (Phenomenex, Torrance, CA, USA) according to methods of Szumny et al. [42].

Qualitative and quantitative GC-MS analyses were performed on Varian CP 3800 + chromatographer (Varian, Palo Alto, CA, USA) coupled with mass spectrometer Saturn 2000 (Varian, Palo Alto, CA, USA). The system was equipped with capillary column Zebron ZB-1MS (10 m × 0.53 mm × 2.65 μm) (Phenomenex, Torrance, CA, USA). Collected data were analyzed by Varian MS Workstation (version 6.5.) (Varian, Palo Alto, CA, USA) equipped with NIST05 Mass Spectral Library with Search Program (National Institute of Standard and Technology, Gaithersburg, MD, USA) and MestreNova 9.0 (trial version, Mestrelab, Research, Santiago de Compostela, Spain).

The normalization of chromatograms of GC-MS was performed as manual correction of the peaks to the baseline. Peaks of multiple compounds were separated into singular peaks according to the main ion in GC-MS. For GC-MS standard mix of *n*-alkanes (C_8_–C_23_) was used.

For the statistical analysis of EOs, peaks of GC-MS chromatograms were integrated and their areas were calculated as a percentage (%) of combined area of all peaks.

Some overlapped peaks were described as single component (if one strongly dominated in peak) or multicomponent mix. Overlapped peaks were divided into single peaks according to major ions concentration.

Single components were identified by comparison of experimental mass spectra and retention indexes with standards of essential oils and literature. Identification data are available as Supplementary Materials.

### 4.3. Preparation and UPLC-PDA-MS Analysis of 70% Ethanol in Water Extract (70EE)

Previously ground research material (propolis and buds) was extracted by 70% ethanol in water (proportion: 1.0 g/10 mL) in an ultrasonic bath. Extraction conditions were set at 400 °C for 45 min and 756 W (90% of ultrasound bath power). Next, extracts were stored at room temperature for 12 h and then filtrated through Wattman No. 10 filtrate paper.

Composition of obtained 70EE was analyzed by Waters Acquity UPLC system (Waters, Milford, CT, USA) equipped with PDA 200–500 nm, mass spectrometer Xevo-Q-TOF (Waters, Milford, CT, USA) and column BEH C18 130 Å, (1.7 μm, 2.1 mm × 100 mm) (Waters, Milford, CT, USA). We used modified method of Shi et al. [42,43]. The technique adopted in this research differed in terms of elution parameters and electrospray ionization parameters (only ESI-NEG mode was used—electrospray ionization in negative mode).

The elution system consisted of acetonitrile/0.1% solution formic acid in water. The gradient elution program began with 20% acetonitrile, and was increased to 30% in 10.70 min → 31% in 15.30 min → 31% in 15.90 min → 32% in 17.00 min → 34% in 18.00 min → 36% in 20.30 min → 40% in 21.50 min → 45% in 25.50 min → 50% in 29.70 min → 100% in 33.00 min → 100% in 36.00 min → 20% in 38.00 min.

Parameters of ESI-NEG were set at capillary voltage of 2.80 kV, sampling cone of 66 kV and extraction cone of 4.0 kV. Collision energy was set at 0, 20, 20–30, 30 and 30–50 kV.

Data were processed using Masslynx 2.0 (Waters, Milford, CT, USA) and MestreNova 9.0 (trial version, Mestrelab, Research, Santiago de Compostela, Spain). Single components were identified by comparison of experimental mass, UV absorption spectra and retention time to standards and literature data. Identification data are available as Supplementary Materials.

The normalization of chromatograms of UPLC was performed as manual correction of the peaks to the baseline. Peaks of multiple compounds were separated into singular peaks according to the spectrum of the main component and main ion in UPLC. UPLC peaks were read at 200–500 nm spectrum for the reason of acquiring average values. For UPLC a mix of acacetin, apigenin, caffeic acid, chrysin, *p*-coumaric acid, ferulic acid, genkwanin, isoferulic acid, kempferol, quercetin dihydrate and pinobanksin was used. As standards of some of the major compounds present in the samples and contributing to the UV spectrum significantly, mostly phenol acid glycerides and monoesters, proved themselves to be unobtainable, it was decided that the analysis could only be reliably carried out as comparison of peak areas calculated as a percentage of total chromatogram peak area.

For statistical analysis of 70EE, peaks of UV chromatograms were integrated in the range of 200–500 nm. Area of integrated peaks was calculated as a percentage (%) of combined area of all peaks.

Some overlapped peaks were described as single component (if one strongly dominated in peak) or multicomponent mix. These data were used in the principal component analysis of 70EE.

### 4.4. Statistical Analysis of 70EE and EOS Composition

Statistical analysis included principal component analysis (PCA) (according to Statsoft, Inc., Kraków, Poland [44]) and Spearman’s coefficient rank [44]. UPLC-PDA (for 70EE analyze compounds) and GC-MS (for EOS analyze) peaks (at least ≥10% in one sample) were used as variables. Reductive analyses were carried out, namely those considering peaks constituting ≥1%, ≥2%, ≥3%, ≥5%, and ≥10% of total area. Meta-analysis of the results demonstrated that ≥10% model explained the data sufficiently, while simultaneously eliminating excessive data input and preventing excessively close groupings of the points, both of which posed a problem in the remaining models, albeit to varying degrees. The authors felt it best to present only the last model in the publication not to make the interpretation of the results excessively bothersome and improve clarity.

Calculations were performed on Statistica 12.5 software (Statsoft, Inc., Kraków, Poland). Calculations of Spearman’s coefficient rank were performed using two models. The first model encompassed *Populus* buds and propolis and the second model contained only some propolis samples.

## 5. Conclusions

Our work has proven that 70EE of propolis is better for identifying propolis plant origins compared to EOs because of the presence of specific plant precursors’ markers in 70EE and a general lack of specific markers in EOs. Some black poplar and aspen buds exhibited differences in EOs and 70EE composition. Among those, higher variability in *Populus* buds EOs than 70EE was observed. For these reasons, polyphenols may be the component which determines bees’ selection of plant material collection for propolis. EOs may however play a role as a secondary attractant. 

## Figures and Tables

**Table 1:** Main components of *Populus* spp. buds essential oils *.

Component	Black Poplars (*Populus nigra* L.)							Aspens (*Populus tremula* L.)					
	PN1	PN2	PN3	PN4	PN5	PN6	PN7	PT1	PT2	PT3	PT4	PT5	PT6
Benzyl alcohol	3.22	4.19	0.07	0.99	0.06	1.03	0.75	1.03	0.29	1.79	4.23	7.04	15.1
2-Phenylethanol	0.34	4.25	tr	0.25	-	1.06	1.00	-	-	-	1.28	3.69	34.5
Benzoic acid	tr	tr	-	-	-	0.12	0.05	-	-	-	0.08	0.07	-
Eugenol	0.20	0.28	tr	-	tr	0.05	0.05	0.30	0.50	0.20	2.28	3.78	14.2
*cis*-β-Cariophyllen	-	0.26	0.23	-	1.18	0.11	0.08	0.32	18.76	24.0	tr	0.09	-
α-Guaien	-	-	-	-	-	-	-	-	6.91	10.2	-	-	-
Prenyl benzoate	-	-	-	21.6	-	20.3	19.8	-	-	-	-	-	-
*ar*-Curcumen	27.2	8.92	-	0.51	-	8.62	9.14	-	-	-	-	-	-
γ-Curcumen	9.50	9.32	-	2.29	3.30	3.79	11.6	-	-	-	-	-	-
δ-Cadinen	7.74	12.0	1.78	4.24	0.40	0.33	0.45	4.79	0.53	0.77	0.30	0.39	0.68
*trans*-Nerolidol	0.06	0.07	-	17.3	-	0.61	0.31	-	-	0.10	-	-	-
Cariophyllene oxide	0.06	0.19	-	-	-	-	-	0.22	18.1	12.0	-	-	-
γ-Eudesmol	-	-	9.90	-	14.4	0.65	0.47	-	-	-	-	-	-
β-Eudesmol	-	-	16.00	-	19.3	0.65	0.12	-	-	-	-	-	-
α-Eudesmol	-	-	49.3	-	22.2	1.18	1.30	-	-	-	-	-	-
Bulnezol	-	-	-	0.69	10.1	3.89	3.68	-	-	-	-	-	-
Benzyl benzoate	0.26	0.20	-	3.65	-	4.47	3.83	45.45	1.12	0.50	26.4	13.5	0.62
Salicyl benzoate	0.07	0.08	-	7.20	-	12.7	10.6	7.02	-	-	16.0	11.7	0.23
*trans*-Benzyl cinnamate	-	-	-	-	-	-	-	-	-	-	26.8	24.7	1.83
Aliphatic component	0.49	0.73	0.05	0.06	0.06	2.38	1.79	3.41	3.40	1.84	4.48	10.5	16.2

Legend: * amount of single component calculated as percent (%) of whole GC-MS chromatogram area; tr, trace concentration of component <0.05%; - substance not detected under detection conditions.

**Table 2:** Main components of propolis essential oils *.

Component	Polish Propolis																Foreign Propolis	
	PR-NW1	PR-NW2	PR-ŚL1	PR-ŚL2	PR-SZ1	PR-SZ2	PR-SZ3	PR-SZ4	PR-LS1	PR-LS2	PR-LS3	PR-LS4	PR-LS5	PR-LS6	PR-S1	PR-S2	PR-GR	PR-CN
Benzyl alcohol	8.38	3.67	5.60	8.47	1.73	2.84	1.26	2.77	2.68	6.39	6.19	0.87	1.46	4.93	6.48	3.23	1.13	1.39
2-Phenylethanol	0.68	0.12	0.59	0.30	0.50	0.38	0.23	0.65	0.21	1.14	1.30	0.43	0.44	2.31	1.22	0.33	5.83	0.21
Benzoic acid	8.52	16.7	1.82	0.78	13.3	33.0	20.7	42.2	13.1	12.1	12.4	5.23	3.75	1.57	12.8	30.2	-	47.1
Eugenol	0.20	0.11	0.11	0.14	0.06	0.22	0.13	0.11	0.25	0.13	0.20	0.08	0.11	0.23	0.20	0.12	0.28	0.39
*cis*-β-Cariophyllen	1.27	0.62	0.13	0.44	0.21	0.34	0.40	0.44	-	tr	0.09	0.43	0.43	0.11	0.16	0.07	-	-
α-Guaien	1.27	0.38	0.22	0.33	0.18	0.41	0.38	0.24	-	-	0.47	0.67	-	0.11	0.30	0.41	2.21	-
Prenyl benzoate	tr	tr	tr	-	tr	-	-	-	-	-	0.65	3.29	3.26	tr	3.85	0.36	-	-
*ar*-Curcumen	-	-	-	-	-	-	-	-	-	-	-	-	-	-	-	-	11.1	1.41
γ-Curcumen	-	-	-	-	-	-	-	-	-	-	-	-	-	-	-	-	6.77	0.84
δ-Cadinen	2.42	0.71	0.31	0.66	0.72	0.78	1.07	0.79	0.67	0.50	0.55	1.62	2.17	2.33	0.55	0.29	0.86	0.44
*trans*-Nerolidol	0.40	0.13	0.16	0.24	0.38	0.22	0.36	0.23	-	0.29	-	0.15	0.19	0.06	0.17	0.30	-	0.26
Cariophyllene oxide	1.96	1.36	0.75	0.50	1.24	1.24	1.44	0.67	0.98	1.04	1.29	2.29	2.12	0.52	5.60	0.52	-	-
γ-Eudesmol	0.53	3.03	2.37	1.14	8.77	2.84	8.71	3.56	3.23	5.33	6.96	14.8	11.3	16.9	1.17	3.16	5.56	5.35
β-Eudesmol	0.32	1.59	1.82	0.33	9.50	2.58	8.49	2.60	1.29	0.57	5.24	10.1	9.91	18.7	1.22	0.66	5.02	5.18
α-Eudesmol	-	0.82	4.38	1.49	9.29	4.10	9.60	4.25	3.46	9.60	10.6	16.8	10.9	17.8	3.08	4.59	6.63	6.72
Bulnezol	-	-	-	-	2.54	1.15	1.30	0.90	-	-	-	3.86	2.84	-	-	-	4.80	-
Benzyl benzoate	11.89	28.6	26.4	23.6	2.65	6.29	7.38	5.80	19.6	20.5	13.7	7.40	11.8	1.28	17.6	19.3	-	6.26
Salicyl benzoate	5.42	0.55	3.33	3.75	1.09	2.04	2.43	1.42	3.74	1.74	3.29	1.38	3.95	0.22	5.01	2.42	-	0.67
*trans*-Benzyl cinnamate	0.68	3.91	5.52	8.97	1.80	3.28	2.72	4.62	3.97	1.33	3.69	0.41	1.21	0.18	3.45	4.93	-	0.41
Aliphatic component	-	0.91	0.09	0.20	0.89	1.05	1.10	0.95	-	0.57	0.88	0.18	0.28	-	0.91	0.77	-	0.47

Legend: * amount of single component calculated as percent (%) of whole GC-MS chromatogram area; tr, trace concentration of component <0.05%; - substance not detected under detection condition.

**Table 3:** General composition of *Populus* spp. buds and Polish propolis essential oils *.

Chemical Component Group	Black Poplars (*Populus nigra* L.)							Aspens (*Populus tremula* L.)						Polish Propolis							
	PN1	PN2	PN3	PN4	PN5	PN6	PN7	PT1	PT2	PT3	PT4	PT5	PT6	PR-NW1	PR-NW2	PR-ŚL1	PR-ŚL2	PR-SZ1	PR-SZ2	PR-SZ3	PR-SZ4
Monoterpenes	0.2	0.2	0.1	0.8	0.1	0.0	0.0	tr	0.1	0.2	1.9	3.4	-	0.3	tr	0.1	0.1	1.0	3.4	1.7	0.9
Sum of sesquiterpenes	90.5	85.5	99.2	45.5	96.7	33.5	41.9	36.8	91.4	92.3	3.4	5.2	3.0	48.6	31.0	28.6	31.4	63.1	29.4	51.5	28.7
Sesuiterpenes hydrocarbons	73.3	66.6	16.2	20.1	10.8	20.7	30.2	19.1	45.3	57.7	2.2	3.2	1.8	13.5	3.8	2.2	3.5	4.4	4.4	6.9	3.9
Oxygenated sesquiterpenoids	17.2	18.9	83.0	25.4	85.9	12.9	11.7	17.7	46.0	34.5	1.2	2.0	1.2	35.1	27.2	26.4	27.8	58.7	25.0	44.7	24.8
Benzoic acid derivates	5.1	5.1	0.1	49.3	1.2	56.5	50.4	54.9	1.5	3.8	76.7	59.9	21.8	37.0	54.4	44.0	47.3	21.1	47.9	35.1	57.6
Phenylethan and propan derivates	1.1	4.8	tr	1.1	tr	2.4	2.1	tr	-	tr	1.3	3.7	34.5	2.4	2.3	2.7	2.2	1.8	2.6	1.2	1.9
Phenols	0.4	0.6	tr	-	0.2	0.1	0.1	0.6	0.6	0.3	6.5	9.1	15.5	1.7	3.7	11.1	4.8	3.5	7.1	3.1	3.9
Aliphatic components	2.3	3.3	0.1	0.3	0.2	6.2	4.8	7.4	6.2	3.1	8.9	17.2	23.3	4.8	6.2	9.3	10.2	6.4	7.7	6.4	5.5
Others	0.4	0.6	0.6	3.0	1.7	1.2	0.8	0.3	0.3	0.4	1.3	1.6	1.9	5.4	2.4	4.3	4.0	3.1	1.8	1.0	1.6
Amount of EOs **	1.0	0.7	4.3	1.5	1.0	0.5	1.2	0.4	0.2	tr	0.1	0.1	0.1	1.1	1.2	2.8	0.7	1.1	0.8	1.8	1.4

Legend: * amount of chemical component group calculated as sum of single components (%, percent of whole GC-MS chromatogram area); ** amount of EOs calculated as percent (%) of mass sample; tr, trace concentration of component <0.05%; - substance not detected under detection conditions.

**Table 4:** General composition of Canadian, German and Polish propolis essential oils *.

Chemical Component Group	Polish Propolis										Foreign Propolis	
	PR-LS1	PR-LS2	PR-LS3	PR-LS4	PR-LS5	PR-LS6	PR-S1	PR-S2	PR-MR	PR-NSW	PR-GR	PR-CN
Monoterpenes	tr	-	-	-	0.2	0.2	0.2	-	-	0.1	0.1	-
Sum of sesquiterpenes	41.2	42.8	39.8	74.6	64.9	77.5	26.6	19.4	14.4	36.6	84.8	31.6
Sesuiterpenes hydrocarbons	2.0	1.6	3.7	13.2	13.6	9.3	4.9	2.1	3.6	5.2	46.9	8.1
Oxygenated sesquiterpenoids	39.2	41.2	36.1	61.4	51.3	68.2	21.6	17.3	10.8	31.4	37.9	23.6
Benzoic acid derivates	44.0	43.2	42.2	20.5	27.5	10.1	53.7	62.6	70.7	44.1	1.9	57.2
Phenylethan and propan derivates	0.7	2.3	2.8	0.8	1.1	4.2	3.3	0.8	0.8	1.5	11.2	3.0
Phenols	6.7	4.3	5.6	1.2	2.0	1.3	4.3	11.0	7.9	9.2	0.8	2.0
Aliphatic components	2.8	4.6	8.1	2.1	3.4	5.5	10.4	5.0	2.9	5.7	0.2	4.3
Others	4.6	2.9	1.6	0.9	1.0	1.3	1.6	1.3	3.3	2.9	1.1	1.8
Amount of EOs **	1.2	0.8	0.4	0.9	0.8	0.5	0.1	0.7	0.1	0.3	1.5	0.7

Legend: * amount of chemical component group calculated as sum of single components (%, percent of whole GC-MS chromatogram area); ** amount of EOs calculated as percent (%) of mass sample; tr, trace concentration of component <0.05%; - substance not detected under detection conditions.

**Table 5:** Main components of *Populus* spp. 70% ethanol in water extracts *.

Component	Black Poplars (*Populus nigra* L.)							Aspens (*Populus tremula* L.)					
	PN1	PN2	PN3	PN4	PN5	PN6	PN7	PT1	PT2	PT3	PT4	PT5	PT6
*p*-Coumaric acid	3.22	2.65	0.14	14.66	1.77	13.91	13.43	3.49	1.67	4.37	2.51	1.15	1.65
** 1,3-Di-*p*-coumaroylglycerol	-	-	-	-	-	-	-	6.68	12.98	3.79	1.47	1.37	2.88
2-Acetyl-1,3-*di*-caffeoylglycerol	-	-	-	-	-	-	-	5.50	0.47	0.31	10.46	17.30	17.37
Acetyl-caffeoyl-*p*-coumaroylglycerol	-	-	-	-	-	-	-	10.97	4.19	3.88	13.83	17.74	16.93
2-Acetylo-3-caffeoyl-1-feruloylglycerol	-	-	-	-	-	-	-	6.48	0.92	0.25	12.28	13.50	5.52
Chrysin	13.02	14.11	4.97	1.47	8.08	2.58	1.69	-	-	-	-	-	-
Sakuranetin	-	0.26	-	0.71	0.26	0.18	0.39	5.01	13.10	9.35	6.47	6.16	5.85
Pinocembrin chalcone	-	4.27	18.07	1.76	21.72	-	-	-	-	-	-	-	-
Galangin	15.25	15.61	10.90	4.46	7.20	2.82	3.22	-	-	-	-	-	-
Pinobanksin-3-*O*-acetate	7.25	7.22	11.36	1.48	5.87	2.21	2.44	-	-	-	-	-	-
** 2-Acetyl-1,3-di-*p*-coumaroylglycerol	-	-	-	-	-	-	-	22.21	37.91	37.74	13.74	11.82	9.63
** 2-Acetyl-*p*-3-coumaroyl-1-feruloylglycerol	-	-	-	-	-	-	-	10.19	7.19	6.49	14.07	10.34	2.02
*p*-Coumaric acid benzyl ester	2.95	0.46	-	1.12	-	1.58	2.13	1.93	2.02	16.39	1.08	0.35	6.78
Pinostrobin chalcone	-	0.94	10.64	5.72	10.77	6.25	1.73	-	-	-	-	-	-
*p*-Coumaric acid cinnamic ester	-	-	-	5.72	0.62	8.55	10.29	-	-	-	-	-	-

Legend: * amount of single component calculated as percent (%) of whole UV chromatogram area; - substance not detected under detection condition; tr, trace concentration of component <0.05%; ** substitution positioning of glycerol was tentatively identified.

**Table 6:** Main components of propolis 70% ethanol in water extracts *.

Component	Polish Propolis																		Foreign Propolis	
	PR-NW1	PR-NW2	PR-ŚL1	PR-ŚL2	PR-SZ1	PR-SZ2	PR-SZ3	PR-SZ4	PR-LS1	PR-LS2	PR-LS3	PR-LS4	PR-LS5	PR-LS6	PR-S1	PR-S2	PR-MR	PR-NSW	PR-GR	PR-CN
*p*-Coumaric acid	8.62	8.96	8.65	8.48	6.87	8.81	12.91	9.13	11.88	9.66	9.95	6.38	12.17	2.09	11.62	11.24	9.42	9.59	0.10	6.48
** 1,3-Di-*p*-coumaroylglycerol	1.53	2.44	2.22	3.75	0.92	1.06	0.79	0.87	1.53	0.86	0.89	0.57	0.76	0.07	0.89	1.02	0.63	1.39	-	0.11
2-Acetyl-1,3-*di*-caffeoylglycerol	0.50	0.71	0.45	0.54	0.18	0.23	0.09	0.06	0.68	0.21	0.29	0.74	0.32	-	0.36	0.24	0.59	0.36	-	-
Acetyl-caffeoyl-*p*-coumaroylglycerol	2.38	3.08	1.93	2.44	1.39	1.47	1.10	1.12	2.55	0.92	1.08	1.70	1.01	-	1.23	1.34	1.76	1.95	-	-
2-Acetylo-3-caffeoyl-1-feruloylglycerol	1.24	1.61	1.05	1.21	0.53	0.53	0.40	0.28	1.37	0.62	0.75	1.05	0.77	-	0.91	0.85	1.14	1.02	-	-
Chrysin	4.98	3.25	4.58	3.38	5.98	5.54	6.55	6.91	1.78	6.81	7.24	4.59	3.89	11.85	4.71	2.02	4.39	7.37	15.25	5.82
Sakuranetin	0.60	0.86	0.58	0.68	1.14	0.90	0.50	0.67	1.07	1.61	0.57	0.55	0.84	-	0.56	1.01	0.43	0.57	-	-
Pinocembrin chalcone	-	-	-	-	-	-	-	-	-	-	-	-	-	-	-	-	-	-	-	-
Galangin	3.38	2.34	3.05	2.30	5.92	6.10	7.10	7.13	2.08	5.65	5.60	5.95	3.20	12.23	4.39	3.19	2.29	5.04	10.59	6.08
Pinobanksin-3-*O*-acetate	1.24	1.15	1.82	1.21	3.92	3.43	4.37	4.93	1.18	2.96	4.20	3.94	1.63	8.92	2.88	1.72	1.92	1.94	4.04	8.73
** 2-Acetyl-1,3-di-*p*-coumaroylglycerol	11.41	14.95	12.16	13.34	6.52	7.32	5.54	6.00	11.12	5.43	7.25	4.52	7.60	0.50	6.33	8.31	5.87	9.61	-	0.84
** 2-Acetyl-*p*-3-coumaroyl-1-feruloylglycerol	4.45	5.68	6.03	5.21	2.62	2.69	2.21	2.43	4.44	2.84	2.99	2.53	3.00	-	3.32	3.72	3.28	3.95	-	0.20
*p*-Coumaric acid benzyl ester	10.71	1.10	9.67	11.39	5.83	6.37	5.12	5.55	10.51	7.63	6.89	4.39	9.01	2.63	8.97	9.31	5.33	5.35	2.18	3.37
Pinostrobin chalcone	-	-	-	-	-	-	-	-	-	-	-	-	-	-	-	-	-	-	-	-
*p*-Coumaric acid cinnamic ester	0.12	0.13	0.25	0.22	-	0.41	0.22	-	0.34	0.32	0.98	2.76	1.66	0.59	2.42	0.80	-	0.22	-	7.90

Legend: * amount of single component calculated as percent (%) of whole UV chromatogram area; - substance not detected under detection condition; trace, concertation of component <0.05%; ** substitution positioning of glycerol was tentatively identified.

## Supplementary Materials

The following are available online, Supplement Table S1A–H: Full results of GC-MS analysis of *Populus* spp. buds essential oils, Supplement Table S2A–G: Full result of GC-MS analysis of Polish propolis essential oils, Supplement Table S3A–H: Full result of GC-MS analysis of Polish propolis essential oils, Supplement Table S4A–E: UPLC-DAD-MS identification of 70% ethanol in water extracts composition, Supplement Table S5A–E: Composition of *Populus* spp. buds 70% ethanol in water extracts, Supplement Table S6A–E: Composition of Polish propolis 70% ethanol in water extracts, Supplement Table S7A–E: Composition of Canadian, German and Polish propolis 70% ethanol in water extracts.
