# Supplementary material for: Profile of Polyphenolic and Essential Oil Composition of Polish Propolis, Black Poplar and Aspens Buds
Source: Molecules. 2018 May 25;23(6):1262. doi: 10.3390/molecules23061262 (PMC6099949; doi:10.3390/molecules23061262)
Supplement: Supplementary file 1 [file molecules-23-01262-s001.pdf]

Suplement 1A. Full results of GC-MS analysis of *Populus* spp. buds essential oils*

Component	RT	KI_{EXP}	KI_{MF}	KI_{NIST}	Black poplars (*Populus nigra* L.)							Aspens (*Populus tremula* L.)					
					PN1	PN2	PN3	PN4	PN5	PN6	PN7	PT1	PT2	PT3	PT4	PT5	PT6
**2-Methylbutanoic acid**	**4.35**	**836**	**ND1**	**839**	tr	tr	-	tr	tr	-	-	-	-	-	-	-	-
*******o***-Xylene**	**5.39**	**879**	**ND1**	**881**	-	-	-	-	-	tr	tr	-	-	-	-	-	-
**Prenyl acetate**	**6.00**	**901**	**902**	**902**	0.07	tr	-	-	-	-	-	-	-	-	-	-	-
***trans*-2-Methyl-2-butenoic acid (Tiglic acid)**	**6.15**	**907**	**ND1**	**946**	0.07	0.05	tr	-	-	tr	-	-	-	-	-	-	-
******α***-Thujene**	**6.58**	**921**	**932**	**925**	tr	tr	-	-	-	-	-	-	-	-	-	-	-
**Benzaldehyde**	**6.76**	**927**	**941**	**933**	0.10	0.08	-	tr	-	tr	tr	tr	-	0.13	0.18	0.15	0.07
***α*-Pinene**	**6.79**	**930**	**936**	**933**	-	-	0.18	tr	0.24	-	tr	-	-	-	-	-	-
****Camphene**	**7.23**	**942**	**950**	**946**	tr	tr	-	-	-	-	-	-	-	-	-	-	-
**Phenol**	**7.77**	**957**	**ND1**	**957**	-	-	-	-	-	0.12	-	-	-	-	0.19	0.20	0.23
***3-Hydroxy-2-methyl-cyclopent-2-enone**	**7.92**	**962**	**ND1**	**977**	0.14	0.06	0.06	-	0.42	-	-	-	-	-	-	-	-
**6-Methyl-5-hepten-2-one**	**8.01**	**965**	**ND1**	**964**	-	-	-	-	-	0.12	-	-	-	-	-	-	-
****Sabinene**	**8.09**	**967**	**973**	**967**	-	-	tr	-	tr	-	-	-	-	-	-	-	-
*******n***-Hexanoic acid**	**8.26**	**971**	**ND1**	**982**	-	-	-	-	-	tr	tr	tr	-	-	-	-	-
*******n***-Octanal**	**8.60**	**980**	**981**	**982**	-	-	-	-	0.06	-	-	-	-	-	-	-	-
******β***-Pinene**	**8.67**	**982**	**978**	**973**	-	-	tr	tr	tr	-	-	-	-	-	-	-	-
*******α***-Phellandrene**	**9.02**	**992**	**1002**	**998**	-	-	tr	-	-	-	-	-	-	-	-	-	-
**Benzyl alcohol**	**9.49**	**1001**	**1006**	**1012**	3.22	4.19	0.07	0.99	0.06	1.03	0.75	1.03	0.29	1.79	4.23	7.04	15.15
*******α***-Terpinene**	**9.54**	**1004**	**1013**	**1010**	-	-	tr	-	-	-	-	-	-	-	-	-	-
**Salicylaldehyde**	**9.57**	**1005**	**1012**	**1029**	-	-	-	0.24	1.06	0.20	0.17	-	0.06	0.64	0.74	0.65	1.08
*******m***-Cymene**	**9.66**	**1007**	**1013**	**1010**	-	-	tr	-	-	-	-	-	-	-	-	-	-
*******β***-Phellandrene**	**9.96**	**1015**	**1023**	**1021**	-	-	tr	-	-	-	-	-	-	-	-	-	-
**Eucalyptol (1,8-Cyneole)**	**10.02**	**1016**	**1024**	**1022**	tr	tr	-	-	tr	-	-	-	-	tr	-	-	-
**Limonene**	**10.03**	**1017**	**1025**	**1023**	-	-	0.12	tr	0.10	tr	tr	-	-	0.06	-	-	-
*******cis***-β-Ocimene**	**10.35**	**1026**	**1029**	**1028**	tr	tr	-	-	-	-	-	-	-	-	-	-	-
**Acetophenone**	**10.56**	**1030**	**1036**	**1042**	0.09	0.15	-	tr	tr	-	-	-	-	tr	-	-	-
*******trans***-β-Ocimene**	**10.82**	**1038**	**1041**	**1038**	-	-	tr	-	tr	-	-	-	-	-	-	-	-
**Benzyl formate**	**11.15**	**1046**	**1060**	**1046**	-	-	-	-	-	-	-	-	-	tr	0.10	0.06	-
*******γ***-Terpinene**	**11.24**	**1047**	**1051**	**1050**	-	-	-	tr	tr	-	-	-	-	-	-	-	-

Suplement 1B.

Component	RT	KI_{EXP}	KI_{MF}	KI_{NIST}	PN1	PN2	PN3	PN4	PN5	PN6	PN7	PT1	PT2	PT3	PT4	PT5	PT6
trans-Linalool oxide (furanoid)	11.61	1055	1058	1064	tr	tr	-	0.39	tr	-	-	0.14	0.15	0.12	-	-	-
o-Guaiacol	11.66	1058	1072	1063	-	-	-	-	-	tr	tr	-	-	-	0.15	0.15	0.35
Methyl benzoate	12.01	1065	1072	1072	-	tr	-	0.08	-	tr	tr	tr	-	-	0.07	0.10	0.19
cis-Linalool oxide (furanoid)	12.21	1065	1072	1072	tr	tr	-	0.09	tr	tr	tr	tr	-	tr	0.34	0.40	0.39
p-Cymene	12.15	1069	1075	1074	tr	tr	-	-	-	-	-	-	-	-	-	-	-
*Terpinolene	12.50	1075	1082	1079	-	-	tr	-	tr	-	-	-	-	-	-	-	-
2-Phenylethanol	12.65	1079	1081	1088	0.34	4.25	tr	0.25	-	1.06	1.00	-	-	-	1.28	3.69	34.53
2-Nonanol	12.71	1082	1085	1089	-	-	-	-	-	tr	tr	-	-	0.05	0.10	0.12	0.18
Linalool	12.78	1082	1086	1086	-	0.22	tr	1.63	0.71	tr	tr	-	-	0.09	0.11	0.13	0.29
*Benzyl component	12.91	1086	ND1	ND1	-	-	-	-	-	-	-	-	-	-	-	-	-
**2-Metylobutyl-3-methylbutanoate	13.12	1090	ND1	1091	-	-	-	-	-	tr	tr	-	-	-	-	-	-
**2-ethenyl-1,1-dimethyl-3-methylene-cyclohexane	13.49	1098	ND1	ND1	-	-	-	0.05	-	-	-	-	-	-	-	-	-
**Veratrole	13.91	1106	1117	1111	-	-	-	0.06	-	-	-	-	-	-	-	-	-
*2-Metyleno-acetophenone	14.19	1115	ND1	ND1	-	-	-	0.27	-	-	-	-	-	-	-	-	-
Benzenepropanal	14.26	1117	ND1	1130	-	-	-	-	-	0.53	0.39	-	-	-	-	-	-
**cis*-Pinocarveol	14.28	1118	1126	1126	tr	tr	-	-	-	-	-	-	-	-	-	-	-
*Walerian 3-metylobut-2-enylu	14.55	1124	ND1	ND1	-	-	-	0.12	-	-	-	-	-	-	-	-	-
**2-Fenylobutadien	14.66	1127	ND1	ND1	-	0.05	-	-	-	-	-	-	-	-	-	-	-
Benzyl acetate	14.78	1130	1134	1139	0.48	0.14	-	-	tr	tr	tr	-	-	tr	0.07	0.06	0.08
**cis*-2-Nonenal	14.94	1134	ND1	1135	-	-	-	-	-	-	-	-	-	-	0.18	0.20	-
tran/cis-Linalool oxide (Pyranoid)	15.45	1144	1144	1164	-	-	-	tr	-	-	-	0.09	0.16	tr	0.14	0.17	0.28
Benzoic acid	16.26	1138	1160	1161	tr	tr	-	-	-	0.12	0.05	-	-	-	0.08	0.07	-
o-Ethylphenol	15.21	1140	ND1	1139	-	-	-	-	-	-	-	-	-	-	-	-	-
Ethyl benzoate	15.24	1141	1150	1142	-	-	-	0.06	-	-	-	-	-	-	-	-	-
**Borneol	15.31	1142	1150	1136	-	tr	tr	-	-	-	-	-	-	-	-	-	-
p-Cymen-8-ol	15.81	1154	ND1	1163	-	-	-	tr	tr	-	-	-	-	-	-	-	-

Suplement 1C.

Component	RT	KI_{EXP}	KI_{MF}	KI_{NIST}	PN1	PN2	PN3	PN4	PN5	PN6	PN7	PT1	PT2	PT3	PT4	PT5	PT6
**Terpinen-4-ol	15.88	1155	1164	1160	-	tr	tr	tr	tr	-	-	-	-	tr	-	-	-
Methyl salicylate	16.29	1163	1171	1175	tr	tr	-	0.44	-	0.59	0.75	0.27	-	0.12	0.86	0.78	0.34
***a*-Terpineol	16.45	1166	1176	1175	tr	tr	tr	-	tr	tr	tr	-	-	0.08	-	-	-
**Safranal	16.39	1167	1182	1173	-	-	-	0.09	-	-	-	-	-	-	-	-	-
β-Cyclocitral	17.48	1187	1195	1173	0.07	0.09	tr	0.75	tr	-	-	-	-	-	-	-	-
2,3-Dihydrobenzofuran	17.53	1188	ND1	1190	-	-	-	0.56	-	0.92	0.68	tr	-	-	0.35	0.55	0.38
***p*-Anisaldehyde	18.24	1206	1218	1224	-	-	-	-	-	tr	tr	-	-	-	-	-	-
2-Phenylethyl acetate	18.83	1221	1235	1228	0.36	0.09	-	tr	-	tr	0.06	-	-	-	-	-	-
***cis*-Cinnamaldehyde	18.94	1223	1234	1237	-	-	-	-	-	-	-	tr	-	-	-	-	-
**Allyl benzoate	19.00	1224	ND1	1239	-	-	-	-	-	-	-	tr	-	-	-	-	-
cis-Geraniol	19.43	1233	1235	1237	0.10	0.13	-	-	-	-	-	tr	0.05	0.06	1.87	3.44	-
n-Propyl benzoate	19.56	1238	1275	1275	-	-	-	0.20	-	0.13	0.12	-	-	-	-	-	-
Cinnamic alcohol	20.86	1269	1274	1275	-	0.09	-	-	-	0.67	0.50	-	-	-	-	-	-
**p*-Vinylguaiacol	21.31	1277	1311	1295	0.24	0.27	-	-	-	tr	tr	0.30	0.13	0.09	4.24	5.26	1.31
2-Hydroxy-5-methylacetophenone	21.90	1290	ND1	1285	-	-	-	-	tr	-	-	-	-	-	-	-	-
Methyl o-methoxybenzoate	21.97	1291	1300	1291	-	0.06	-	1.35	-	-	-	-	-	-	-	-	-
1-Methylpropyl benzoate	22.12	1294	1306	1302	-	-	-	3.18	-	-	-	-	-	-	-	-	-
**Methyl geramate	22.26	1297	1306	1308	0.12	0.11	-	-	-	-	-	-	-	-	-	-	-
**Methyl decanoate	22.60	1306	1300	1308	-	tr	-	-	-	-	-	-	-	-	-	-	-
Methyl benzoylformate	22.73	1312	1313	ND1	-	-	-	1.48	-	-	-	-	-	-	-	-	-
**8,9-Dehydro-cycloisolongifolene	22.98	1325	1320	ND1	-	-	tr	tr	-	-	-	-	-	-	-	-	-
Eugenol	23.26	1331	1331	1335	0.20	0.28	tr	-	tr	0.05	0.05	0.30	0.50	0.20	2.28	3.78	14.21
3,4-Dimethoxystyrene	23.44	1337	1337	1354	-	-	-	-	0.14	-	-	-	-	-	-	-	-
Butyl benzoate	23.79	1349	1556	1351	-	-	-	0.72	-	1.60	1.82	-	-	-	-	-	-
α-Cubebene	23.79	1349	1355	1365	-	-	tr	-	tr	-	-	0.11	0.06	0.13	-	-	-
**Vanillin	23.93	1354	1355	1360	-	-	-	-	-	-	-	tr	-	-	-	-	-
**Cyklosativene	24.15	1362	1390	ND1	-	tr	0.05	-	tr	-	-	-	-	-	-	-	-

Suplement 1D.

Component	RT	KI$_{EXP}$	KI$_{MF}$	KI$_{NIST}$	PN1	PN2	PN3	PN4	PN5	PN6	PN7	PT1	PT2	PT3	PT4	PT5	PT6
**Benzyl 3-methyl butanoate	24.20	1363	ND1	1371	0.05	0.05	-	-	-	-	-	-	-	-	-	-	-
**Cypera-2,4-diene	24.33	1373	1365	ND1	-	-	0.07	-	-	-	-	-	-	-	-	-	-
α-Ylanglene	24.40	1370	1376	1370	0.35	0.30	0.61	0.27	0.36	-	-	0.09	tr	0.06	-	-	-
α-Copaene	24.52	1374	1379	1376	1.43	1.65	tr	0.59	tr	tr	tr	0.82	0.16	0.52	0.34	0.51	0.17
***β*-Bourbonene	24.67	1379	1386	1382	-	-	-	-	-	-	-	tr	0.05	tr	0.05	0.05	tr
***β*-Elemene	24.73	1381	ND1	1388	-	-	-	-	-	-	-	-	0.06	0.08	-	-	-
***β*-Cubebene	24.76	1382	1378	ND1	-	-	0.12	-	-	-	-	tr	-	0.06	-	-	-
7-*epi*-Sesquithujene	24.84	1385	1387	1385	tr	0.16	-	-	tr	-	-	-	-	-	-	-	-
**Cyperene	25.11	1394	1402	1398	-	-	-	-	-	-	-	tr	-	tr	-	-	-
2-Hydroxy-2-methylpropyl benzoate	25.06	1392	ND1	1406	-	-	-	tr	-	0.57	0.53	-	-	-	-	-	-
**Cedrene	25.13	1392	ND1	1406	-	-	tr	-	-	-	-	-	-	-	-	-	-
**Sesquithujene	25.15	1395	1399	1411	-	tr	-	-	tr	-	-	-	-	-	-	-	-
α-Cedrene	25.20	1397	1413	1418	0.17	0.14	tr	0.06	tr	tr	0.09	tr	-	-	-	-	-
cis-*α*-Bergamotene	25.37	1400	1409	1409	1.39	1.31	-	-	0.14	0.64	0.91	-	-	-	0.11	0.11	-
cis-*β*-Caryophyllene	25.39	1401	1409	1409	-	0.26	0.23	-	1.18	0.11	0.08	0.32	18.76	24.01	tr	0.09	-
2-Methylbutyl benzoate	25.33	1402	1419	1422	-	-	-	5.98	-	4.61	5.97	-	-	-	-	-	-
**3-Methylbut-3-enyl benzoate	25.41	1408	ND1	1426	-	-	-	0.13	-	-	-	-	-	-	-	-	-
β-Copaene	25.50	1414	1430	1426	tr	0.17	0.05	0.06	0.56	tr	tr	0.17	tr	0.08	tr	tr	tr
*Sesquiterpene hydrocarbon	25.58	1419	ND1	ND1	-	-	tr	-	-	-	-	-	-	-	-	-	-
trans-*α*-Bergamoten	25.70	1427	1434	1443	2.87	3.28	-	0.49	0.25	1.42	0.76	-	-	-	0.34	0.35	-
α-Guaiene	25.73	1430	1434	1444	-	-	-	-	-	-	-	-	6.91	10.24	-	-	-
***cis*-Muurola-3,5-diene	25.83	1436	1447	1454	-	-	-	-	tr	-	-	tr	0.10	-	-	-	-
**Cadena-3 5-diene	25.85	1437	1448	*1458	-	0.14	-	-	0.06	-	-	0.07	-	-	-	-	-
**Guaia-6,9-diene	25.86	1438	1448	1443	-	-	-	0.14	-	-	-	-	-	-	-	-	-
**Isogermacrene D	25.89	1440	1445	1437	-	-	-	-	-	0.09	0.13	-	-	-	-	-	-
α-Humulene	25.94	1444	1455	1447	0.13	0.39	0.31	0.05	0.40	-	-	0.15	8.30	8.77	-	-	-
cis-*β*-Farnesene	25.99	1447	1446	1448	0.36	1.13	0.37	0.23	0.18	0.28	0.14	-	-	-	-	-	-

Suplement 1E.

Component	RT	KI_{EXP}	KI_{MF}	KI_{NIST}	PN1	PN2	PN3	PN4	PN5	PN6	PN7	PT1	PT2	PT3	PT4	PT5	PT6
**Muurola-4,11-diene	26.03	1449	1458	ND1	-	-	-	-	0.05	-	-	-	-	-	-	-	-
Aromadendrene	26.04	1450	1443	1447	-	-	-	-	-	-	-	-	tr	0.18	-	-	-
allo-Aromadendrene	26.05	1451	1462	1460	1.57	2.28	0.75	0.31	-	0.08	-	0.89	0.07	0.08	0.17	0.17	0.09
Prenzl benzoate	26.08	1453	1450	1477	-	-	-	21.57	-	20.35	19.83	-	-	-	-	-	-
(*cis, cis*)-*α*-Farnesene	26.13	1456	1460	1469	1.91	2.04	-	-	0.09	0.53	0.75	-	-	-	-	-	-
Sesquiterpene hydrocarbon	26.14	1463	ND1	ND1	-	-	-	-	-	-	-	-	0.15	0.12	-	-	-
Phenethyl 2-methylbutyrate	26.20	1461	ND1	1469	0.33	0.21	-	0.54	-	0.12	0.11	-	-	-	-	-	-
7aH,10bH-Cadina-1(6),4-diene	26.20	1461	1460	1468	0.71	1.68	-	0.19	tr	-	-	0.30	-	-	-	-	-
γ-Gurjunene	26.22	1462	1472	1470	-	-	-	-	0.14	0.11	0.23	-	0.86	1.62	-	-	-
γ-Muurolene	26.23	1466	1474	1472	2.31	2.47	1.22	1.18	0.26	-	-	1.26	-	-	0.14	0.23	0.07
(+)-9-*epi*-*β*-Caryophyllene	26.24	1463	ND1	1464	-	-	-	-	-	-	-	-	0.49	0.63	-	-	-
α-Amorphene	26.29	1471	1477	1465	-	-	-	-	-	-	-	-	-	-	0.27	0.43	0.09
ar-Curcumene	26.29	1467	1473	1473	27.18	8.92	-	0.51	-	8.62	9.14	-	-	-	-	-	-
γ-Curcumene	26.33	1469	1475	1478	9.50	9.32	-	2.29	3.30	3.79	11.64	-	-	-	-	-	-
β-Eudesmene	26.42	1475	1486	1482	0.23	0.44	2.68	0.41	0.23	-	-	0.06	2.93	3.14	-	-	-
Germacrene D	26.47	1479	1479	1477	0.20	0.18	-	-	-	-	-	0.29	-	-	-	-	-
Eremophilene	26.47	1478	1486	1486	-	-	-	1.22	-	0.71	0.85	-	-	-	-	-	-
epi-Cubebol	26.52	1484	1490	1489	-	-	-	-	-	-	-	0.28	-	-	-	-	-
γ-Amorphene	26.53	1482	1492	1491	0.39	0.78	-	0.68	0.32	-	-	-	-	-	-	-	-
α-Selinene	26.58	1486	1494	1491	-	-	2.01	1.10	0.53	0.53	0.77	-	2.60	3.08	0.05	0.11	-
(+)-Valencene	26.58	1486	1494	1486	-	-	-	-	-	-	-	-	0.60	0.82	-	-	-
Epizonarene	26.59	1486	1494	1504	1.14	2.06	-	-	-	-	-	-	-	-	-	-	-
α-Muurolene	26.64	1488	1496	1496	2.09	2.61	-	0.68	-	-	-	2.20	-	-	0.09	0.17	0.18
Conocephalenol	26.65	1490	1497	ND1	-	-	-	1.28	-	-	-	-	-	-	-	-	-
α-Farnesene	26.70	1493	1498	1496	-	-	-	2.14	-	0.14	-	-	-	-	0.08	0.14	-
β-Himachalene	26.71	1494	1500	1498	1.97	0.24	-	-	-	1.62	1.77	-	-	-	-	-	-
α-Bulnesene	26.72	1494	1503	1502	-	-	-	-	-	1.16	1.75	-	1.97	2.64	-	-	-

Suplement 1F.

Component	RT	KI_{EXP}	KI_{MF}	KI_{NIST}	PN1	PN2	PN3	PN4	PN5	PN6	PN7	PT1	PT2	PT3	PT4	PT5	PT6
*Oxygenated sesquiterpenoid	26.80	1500	ND1	ND1	-	-	-	-	0.15	-	-	-	-	-	-	-	-
γ-Cadinene	26.81	1500	1507	1507	5.11	8.36	-	2.10	1.14	-	-	3.99	0.14	0.18	0.11	0.19	0.31
trans and *cis*-Calamene	26.85	1501	1520	1510	2.30	1.91	0.67	0.50	-	-	-	2.24	0.25	0.26	-	-	-
β-Curcumene	26.88	1503	1503	ND1	-	-	-	-	0.64	-	-	-	-	-	-	-	-
δ-Cadinene	26.92	1511	1526	1516	7.74	12.05	1.78	4.24	0.40	0.33	0.45	4.79	0.53	0.77	0.30	0.39	0.68
**α*-Copaen-11-ol	26.99	1517	ND1	ND1	-	-	1.28	-	2.71	-	-	-	-	-	-	-	-
Cadina-1,4-diene	27.03	1522	1523	1525	0.57	0.87	2.43	0.28	0.21	-	-	-	-	-	-	-	-
*4,5,9,10-Dehydroisolongifolene	27.03	1521	ND1	ND1	-	-	-	-	-	-	-	0.20	0.20	0.11	-	-	-
α-Calacorene	27.09	1524	1527	1532	0.78	0.42	-	0.13	-	tr	tr	0.47	tr	0.09	-	-	-
α-Cadinene	27.10	1526	1530	1528	0.69	0.78	-	0.19	-	-	-	0.54	-	-	0.11	0.19	0.11
trans-γ-Bisabolene	27.13	1531	1521	1525	0.16	0.21	-	0.05	-	0.18	0.29	-	-	-	-	-	-
**trans-Z-α*-Bisabolene epoxide	27.15	1532	ND1	1540	tr	tr	-	-	0.26	-	-	-	0.62	0.48	-	-	-
Selina-3,7(11)-diene	27.17	1534	1542	1549	-	-	1.74	-	0.10	-	-	-	-	-	-	-	-
Elemol	27.29	1545	1541	1537	-	-	0.24	-	-	-	-	-	-	-	-	-	-
trans-Nerolidol	27.29	1545	1553	1540	0.06	0.07	-	17.28	-	0.61	0.31	-	-	0.10	-	-	-
Germacrene B	27.32	1548	1552	1549	-	-	0.96	-	0.09	0.20	0.30	-	-	-	-	-	-
ar-Turmerol	27.38	1553	ND1	1564	0.10	0.13	-	-	-	-	-	-	-	-	-	-	-
α-Cedrene oxide	27.44	1562	1571	ND1	-	-	-	-	-	-	-	-	1.84	1.61	-	-	-
*(+)-Spathulenol	27.45	1560	1572	1568	tr	tr	-	0.23	-	-	-	-	-	-	-	-	-
*Oxygenated sesquiterpenoid	27.51	1565	ND1	ND1	-	-	-	-	-	-	-	-	0.87	0.83	-	-	-
Caryophyllene oxide	27.55	1569	1552	1574	0.06	0.19	-	-	-	-	-	0.22	18.11	12.04	-	-	-
(-)-Gleenol	27.57	1570	1574	1577	0.22	0.26	-	-	-	-	-	0.23	-	-	-	-	-
*(-)-Spathulenol	27.63	1576	ND1	1577	0.32	0.22	-	1.20	0.21	-	-	-	-	-	-	-	-
**Viridiflorol	27.67	1580	1592	1582	0.07	0.08	-	-	-	-	-	-	-	-	-	-	-
α-Guaiol	27.72	1584	1593	1589	-	-	-	-	8.77	3.34	3.08	-	-	-	0.13	0.31	0.35
**trans-Z-α*-Bisabolene epoxide	27.74	1581	ND1	1586	-	-	-	-	-	-	-	-	0.86	0.75	0.21	0.37	-
*Isoaromadendrene epoxide	27.81	1588	ND1	1590	-	-	-	0.44	-	-	-	-	-	-	-	-	-

Suplement 1G.

Component	RT	KI_{EXP}	KI_{MF}	KI_{NIST}	PN1	PN2	PN3	PN4	PN5	PN6	PN7	PT1	PT2	PT3	PT4	PT5	PT6
Ledol	27.83	1594	1602	ND1	0.28	0.37	-	-	-	-	-	-	-	-	-	-	-
Humulene epoxide (II)	27.85	1595.72	1602	1599	-	-	-	-	-	0.14	-	0.33	5.85	4.63	-	-	-
Neointermedeol	27.91	1601	ND1	1601	-	-	-	-	-	-	-	-	0.84	0.57	-	-	-
Di-epi-1,10-cubenol	27.93	1603	1615	1609	0.86	1.09	-	0.24	0.25	-	-	0.78	2.13	2.61	-	-	-
Muurola-4,10(14)-dien-1-β-ol	27.98	1608	ND1	1609	0.27	0.24	-	0.07	-	-	-	0.34	-	-	-	-	-
Epicubenol	28.03	1613	ND1	1617	1.24	1.18	-	-	-	tr	-	1.00	-	-	-	-	-
10-epi-γ-Eudesmol	28.04	1615	1609	1615	-	-	4.47	-	4.12	-	-	-	-	-	-	-	-
Cubenol	28.06	1616	1630	1620	0.23	0.36	-	0.30	-	-	-	0.10	-	-	0.10	0.13	-
Caryophylla-4(12),8(13)-dien-5α-ol	28.08	1619	ND1	1623	-	-	-	-	-	-	-	-	4.14	1.89	-	-	-
γ-Eudesmol	28.11	1621	1618	1618	-	-	9.90	-	14.43	0.65	0.47	-	-	-	-	-	-
τ-Cadinol	28.15	1626	1633	1631	5.99	6.70	-	1.14	-	0.19	0.20	6.15	0.78	0.82	0.20	0.33	0.42
Agarospirol	28.18	1628	1635	1631	-	-	1.61	-	2.33	0.25	0.16	-	-	-	-	-	-
δ-Cadinol	28.21	1632	ND1	1627	1.29	1.51	-	0.48	-	-	-	2.12	-	-	0.08	0.15	0.37
(+)-Intermedeol	28.25	1636	ND1	1645	-	-	-	-	-	-	-	-	2.72	2.59	-	-	-
β-Eudesmol	28.26	1636	1641	1636	-	-	16.00	-	19.31	0.65	0.12	-	-	-	-	-	-
*Oxygenated sesquiterpenoid	28.28	1639	ND1	ND1	-	-	-	-	-	-	-	-	2.43	1.82	-	-	-
α-Cadinol	28.28	1642	1643	1643	3.82	4.16	-	1.43	-	-	-	5.56	-	-	0.45	0.71	0.05
α-Eudesmol	28.30	1640	1659	1654	-	-	49.28	-	22.18	1.18	1.30	-	-	-	-	-	-
*Ledene oxide (II)	28.35	1645	ND1	1646	-	-	-	0.16	-	-	-	-	2.14	1.62	-	-	-
β-Bisabolol	28.42	1653	1659	ND1	0.63	0.55	0.13	-	-	-	-	-	-	-	-	-	-
Bulnesol	28.43	1654	1665	1655	-	-	-	0.69	10.07	3.89	3.68	-	-	-	-	-	-
Cadalene	28.45	1655	1659	1660	0.24	0.25	-	0.27	-	-	-	0.35	-	-	-	-	-
*Eudesma-3,11-dien-8-one	28.53	1664	1666	1646	-	-	-	-	-	-	-	-	0.93	0.31	-	-	-
α-Bisabolol	28.58	1668	1673	1680	1.28	1.42	-	-	0.33	1.93	2.39	-	-	-	-	-	-
*<cis-14-nor->Muurol-5-en-4-one	28.59	1675	ND1	1673	-	-	-	-	-	-	-	0.16	-	-	-	-	-
*Eudesma-4(15),7-dien-1β -ol	28.64	1675	1671	1685	-	-	-	-	-	-	-	0.05	-	0.76	-	-	-
**Eudesm-7(11)-en-4-ol	28.66	1677	1676	1682	-	-	0.07	-	0.11	-	-	-	-	-	-	-	-

Suplement 1H.

Component	RT	KI_{EXP}	KI_{MF}	KI_{NIST}	PN1	PN2	PN3	PN4	PN5	PN6	PN7	PT1	PT2	PT3	PT4	PT5	PT6
*(+)-Cyperotundon	28.70	1681	1684	1685	-	-	-	-	-	-	-	-	0.54	0.26	-	-	-
Germacra-4(15),5,10(14)-trien-1α-ol	28.70	1680	1680	ND1	-	-	-	0.15	-	-	-	-	-	-	-	-	-
*Oxygenated sesquiterpenoid	28.82	1692	ND1	ND1	-	-	-	-	-	-	-	-	1.20	0.84	-	-	-
**(E,E)-Farnesol	28.83	1693	1694	1704	0.11	0.06	-	-	-	-	-	-	-	-	-	-	-
Methyl tetradecanoate	28.94	1704	ND1	1708	-	-	-	-	-	0.18	0.20	-	-	-	1.56	2.16	2.80
Benzyl benzoate	29.10	1726	1730	1732	0.26	0.20	-	3.65	-	4.47	3.83	45.45	1.12	0.50	26.37	13.55	0.62
**γ-Costol	29.20	1737	1730	ND1	-	-	-	-	tr	-	-	-	-	-	-	-	-
β-Costol	29.32	1753	1754	1769	-	-	-	-	0.22	-	-	-	-	-	-	-	-
**α-Costol	29.48	1772	1761	1757	-	-	-	-	0.08	-	-	-	-	-	-	-	-
Selina-4α,11-diol	29.60	1787	ND1	1780	-	-	-	-	0.31	-	-	-	-	-	-	-	-
Phenylethyl benzoate	29.83	1813	1815	1828	0.86	0.17	-	1.56	-	9.37	5.50	-	-	-	-	-	0.60
Benzyl salicylate	29.98	1831	1847	1836	0.07	0.08	-	7.20	-	12.72	10.56	7.02	-	-	16.04	11.71	0.23
*Aliphatic alcohol	30.41	1900	ND1	ND1	-	-	-	-	tr	-	-	-	-	-	-	-	-
Methyl 4,7,10,13-hexadecatetraenote	30.46	1906	ND1	ND1	0.34	0.38	-	-	-	-	-	0.67	0.46	0.13	-	-	-
Phenethyl salicylate	30.60	1926	ND1	ND1	-	-	-	tr	-	0.48	0.28	-	-	-	-	-	-
*Benzyl component	30.70	1939	ND1	ND1	-	-	-	0.34	-	0.23	0.12	0.97	-	-	-	-	-
**cis*-Benzyl cinnamate	31.32	2029	ND1	1959	-	-	-	-	-	-	-	-	-	-	1.20	1.08	1.55
trans-Benzyl cinnamate	31.51	2059	2023	ND1	-	-	-	-	-	-	-	-	-	-	26.81	24.66	1.83
*Aliphatic ester	31.63	2077	ND1	ND1	0.75	1.11	-	0.13	tr	-	-	0.87	1.07	0.13	-	-	-
n-Heneicosane	31.74	2094	2100	2100	-	-	-	-	-	0.22	0.13	0.74	-	0.33	-	-	-
*Benzyl dodecanoate	31.95	2128	ND1	2097	-	-	-	-	-	-	-	0.06	-	0.63	-	-	-
*Aliphatic component	32.33	2189	ND1	ND1	-	-	-	-	-	0.16	0.15	-	-	-	0.57	0.72	1.71
*Aliphatic component	32.77	2265	ND1	ND1	-	-	-	-	-	0.07	tr	-	-	-	-	-	-
*Aliphatic component	32.89	2286	ND1	ND1	-	-	-	-	-	-	-	0.78	0.88	0.35	-	-	-
Tricosane	32.91	2290	2301	2300	0.50	0.80	tr	0.12	tr	2.82	2.11	0.75	-	0.16	1.24	2.22	-
*Aliphatic component	33.47	ND	ND1	ND1	0.11	0.18	-	-	-	-	-	0.12	0.37	0.06	-	-	-
*Aliphatic component	33.85	ND	ND1	ND1	-	-	-	-	-	0.28	0.32	-	-	-	0.77	1.24	2.42
*Aliphatic component	34.00	ND	ND1	ND1	0.49	0.73	0.05	0.06	0.06	2.38	1.79	3.41	3.40	1.84	4.48	10.53	16.22

Suplemet 1 Legend

* – amount of single component calculated as percent (%) of whole GC-MS chromatogram area

** – possible xenobiotic

tr – trace concentration of component <0,05%

"–" - substance not detected under detection conditions

KI_{EXP} – Experimental retention index

KI_{MF} – Retention index according to Terpenoid Library [1]

KI_{NIST} – Retention index according to NIST Chemistry WebBook [2]

ND – Retention Indices was not determined

ND1 – No data for DB-1 cappilary column type

Literature:

1. König, W.A.; Joulain, D.; Grasse, R.S.; Hochmuth, D.H. Terpenoids and related constituents of essential oils, Hochmuth Scientific Consulting. Aviable on: http://massfinder.com/wiki/Terpenoids_Library. (accessed on: 15th May 2017)
2. National Institute of Standard and Technology. NIST Chemistry WebBook. Old York Road, Ringoes, USA, National Institute of Standards and Technology. Aviable on: http://webbook.nist.gov/chemistry/ (accessed on: 15th May 2017)

Suplement 2A. Full result of GC-MS analysis of Polish propolis essential oils

Component	RT	KI_{EXP}	KI_{MF}	KI_{NIST}	PR-NW1	PR-NW1	PR-ŚL1	PR-ŚL2	PR-SZ1	PR-SZ2	PR-SZ3	PR-SZ4
***trans*-2-Hexenal	4.15	826	832	827	tr	-	0.17	0.10	-	-	-	-
2-Methylbutanoic acid	4.35	836	ND1	839	0.16	tr	0.12	0.06	0.10	-	0.06	tr
***trans*-3-Hexen-1-ol	4.41	838	ND1	839	tr	-	-	-	-	-	-	-
**Ethylbenzene	4.59	847	843	850	-	-	-	-	-	-	-	-
***n*-Hexanol	4.72	852	837	854	tr	-	-	-	-	-	-	-
***p*-Xylene	4.81	856	ND1	860	-	-	-	-	-	-	-	-
**3-methyl-3-buten-1-ol acetate	5.02	865	ND1	862	tr	-	-	-	-	-	-	-
**Styrene	5.21	872	ND1	878	tr	-	tr	tr	tr	tr	tr	tr
***n*-Heptanal	5.36	878	882	880	tr	-	tr	tr	-	-	-	-
trans-2-Methyl-2-butenoic acid (Tiglic acid)	6.15	907	ND1	946	0.13	tr	0.16	0.12	0.26	0.12	0.11	0.20
***trans*-1-Ethyl-2-methyl-cyklohexane	6.62	923	ND1	968	tr	-	0.10	0.06	-	-	-	-
Benzaldehyde	6.76	927	941	933	0.87	0.28	0.28	0.27	0.14	0.23	0.14	0.12
α-Pinene	6.79	930	936	933	0.09	-	0.13	0.12	tr	tr	tr	tr
**Camphene	7.23	942	950	946	tr	-	-	-	-	-	-	-
**6-Methyl-5-hepten-2-one	8.01	965	ND1	964	tr	tr	tr	tr	-	-	-	-
n-Hexanoic acid	8.26	971	ND1	982	0.34	0.13	0.14	0.26	0.07	0.26	0.13	0.19
n-Octanal	8.60	980	981	982	0.29	0.07	0.32	0.33	0.11	0.14	0.05	0.08
**2-Methyl-5-(prop-1-en-2-yl)-2-vinyl tetrahydrofuran	9.09	990	ND1	983	tr	-	tr	tr	-	-	-	-
3-*δ*-Carene	9.47	1001	1000	998	-	-	-	-	-	-	-	-
Benzyl alcohol	9.49	1001	1006	1012	8.38	3.67	5.60	8.47	1.73	2.84	1.26	2.77
Eucalyptol (1,8-Cyneole)	10.02	1016	1024	1022	0.22	0.07	0.12	0.13	0.13	0.17	0.31	0.10
Limonene	10.03	1017	1025	1023	-	-	0.12	0.13	-	-	-	-
Acetophenone	10.56	1030	1036	1042	0.37	0.09	0.12	0.14	0.07	0.07	0.05	0.06
**Benzyl formate	11.15	1046	1060	1046	0.05	tr	tr	tr	-	-	-	-
***γ*-Terpinene	11.24	1047	1051	1050	tr	-	-	-	-	-	-	-
trans-Linalool oxide (furanoid)	11.61	1055	1058	1064	0.56	0.14	0.23	0.28	0.09	0.14	0.10	0.13

Suplement 2B.

Component	RT	KI_{EXP}	KI_{MF}	KI_{NIST}	PR-NW1	PR-NW1	PR-ŚL1	PR-ŚL2	PR-SZ1	PR-SZ2	PR-SZ3	PR-SZ4
**Methyl benzoate**	**12.01**	**1065**	**1072**	**1072**	0.25	0.16	0.08	0.09	tr	0.09	tr	tr
***cis*-Linalool oxide (furanoid)**	**12.21**	**1065**	**1072**	**1072**	0.05	0.31	0.13	0.15	0.05	0.08	0.05	0.08
****6-Methyl-3,5-heptadien-2-one**	**12.47**	**1077**	**ND1**	**1076**	0.05	0.05	-	-	tr	tr	tr	tr
****Terpinolene**	**12.50**	**1075**	**1082**	**1079**	-	-	tr	tr	-	-	-	-
**2-Phenylethanol**	**12.65**	**1079**	**1081**	**1088**	0.22	0.68	0.59	0.30	0.50	0.38	0.23	0.65
**2-Nonanol**	**12.71**	**1082**	**1085**	**1089**	0.56	0.70	0.75	0.79	0.27	0.32	0.13	0.15
**Linalool**	**12.78**	**1082**	**1086**	**1086**	0.46	0.65	0.17	0.23	0.11	0.14	0.13	0.18
***2-ethenyl-1,1-dimethyl-3-methylene-cyclohexane**	**13.49**	**1098**	**ND1**	**ND1**	0.07	tr	-	-	-	-	-	-
**Camphor**	**14.09**	**1113**	**1123**	**1123**	0.17	0.08	tr	-	tr	0.18	tr	tr
**Benzenepropanal**	**14.26**	**1117**	**ND1**	**1130**	0.14	0.17	0.14	0.14	0.14	0.07	0.05	0.09
*****p*-Vinylanisole**	**14.63**	**1126**	**1134**	**1152**	tr	0.10	tr	tr	tr	tr	tr	tr
****2-Fenylobutadien**	**14.66**	**1127**	**ND1**	**ND1**	tr	0.05	tr	tr	0.07	tr	tr	tr
**Benzyl acetate**	**14.78**	**1130**	**1134**	**1139**	0.19	0.21	tr	tr	tr	0.05	tr	0.11
*****cis*-2-Nonenal**	**14.94**	**1134**	**ND1**	**1135**	tr	0.07	tr	tr	-	-	-	-
***tran/cis*-Linalool oxide (Pyranoid)**	**15.45**	**1144**	**1144**	**1164**	tr	0.06	0.14	0.25	-	-	-	-
**Benzoic acid**	**16.26**	**1138**	**1160**	**1161**	4.91	8.52	1.82	0.78	13.28	32.97	20.71	42.16
**Methyl salicylate**	**16.29**	**1163**	**1171**	**1175**	-	-	-	-	-	-	-	-
*****a*-Terpineol**	**16.45**	**1166**	**1176**	**1175**	0.06	0.05	-	-	-	-	-	-
****Verbenone**	**16.77**	**1175**	**1183**	**1170**	tr	0.05	-	-	-	-	-	-
***n*-Decenal**	**17.17**	**1183**	**ND1**	**1240**	0.73	0.96	1.82	0.78	-	-	-	-
***β*-Cyclocitral**	**17.48**	**1187**	**1195**	**1173**	0.07	0.07	-	-	-	-	-	-
**2,3-Dihydrobenzofuran**	**17.53**	**1188**	**ND1**	**1190**	0.09	0.27	1.51	0.56	1.21	-	-	-
****Methyl nonanoate**	**18.01**	**1200**	**ND1**	**1208**	-	-	tr	-	-	-	-	-
*****p*-Anisaldehyde**	**18.24**	**1206**	**1218**	**1224**	-	-	tr	tr	-	-	-	-
****Benzyl acrylate**	**18.53**	**1213**	**ND1**	**ND1**	0.11	0.09	0.07	0.11	tr	0.05	-	-
***cis*-Cinnamaldehyde**	**18.94**	**1223**	**1234**	**1237**	0.75	0.15	0.09	0.15	0.07	0.54	0.06	-
**Allyl benzoate**	**19.00**	**1224**	**ND1**	**1239**	-	0.59	0.44	0.54	0.19	-	0.20	0.39

Suplement 2C.

Component	RT	KI$_{EXP}$	KI$_{MF}$	KI$_{NIST}$	PR-NW1	PR-NW1	PR-ŚL1	PR-ŚL2	PR-SZ1	PR-SZ2	PR-SZ3	PR-SZ4
cis-Geraniol	19.43	1233	1235	1237	0.12	tr	0.07	0.10	0.10	-	0.06	0.09
***p*-Ethylguaiacol	19.98	1247	ND1	1252	tr	-	-	-	-	-	-	-
***n*-Nonanoic acid	20.39	1257	1263	1272	0.06	tr	0.07	0.14	tr	-	-	-
Cinnamic alcohol	20.86	1269	1274	1275	tr	0.06	0.12	0.11	0.16	-	tr	0.11
Thymol	20.85	1267	1267	1270	-	-	-	-	0.86	3.44	1.64	0.77
**p*-Vinylguaiacol	21.31	1277	1311	1295	0.83	1.26	8.30	0.41	2.23	4.93	2.09	1.90
*Phenacyl alcohol	21.41	1279	ND1	ND1	0.82	1.79	1.51	1.32	0.72	1.47	0.67	0.89
**2-Hydroxy-5-methylacetophenone	21.90	1290	ND1	1285	0.06	tr	0.05	0.05	0.06	0.05	tr	tr
Methyl decanoate	22.60	1306	1300	1308	tr	tr	tr	0.05	-	-	-	-
***cis*-4-Phenyl-3-buten-2-one	22.81	1315	ND1	1323	0.06	tr	0.05	tr	0.08	0.07	0.05	0.07
**8,9-Dehydro-cycloisolongifolene	22.98	1325	1320	ND1	0.10	tr	tr	tr	tr	tr	tr	tr
Eugenol	23.26	1331	1331	1335	0.20	0.11	0.11	0.14	0.06	0.22	0.13	0.11
3,4-Dimethoxystyrene	23.44	1337	1337	1354	0.19	0.08	0.13	0.14	0.06	0.05	tr	tr
**Aliphatic component	23.55	1341	ND1	ND1	tr	tr	-	-	-	-	-	-
Vanillin	23.93	1354	1355	1360	0.38	1.99	2.30	3.54	1.05	1.82	0.78	1.79
**o*-Vanillin	24.15	1362	ND1	ND1	tr	0.23	0.26	0.61	0.09	0.12	tr	0.11
Cypera-2,4-diene	24.33	1373	1365	ND1	tr	-	-	-	-	-	-	-
α-Ylanglene	24.40	1370	1376	1370	0.08	tr	0.09	0.13	0.06	tr	0.09	0.06
α-Copaene	24.52	1374	1379	1376	0.52	0.13	-	-	0.13	0.18	0.30	0.15
***β*-Bourbonene	24.67	1379	1386	1382	tr	-	-	-	tr	tr	0.10	-
***β*-Cubebene	24.76	1382	1378	ND1	tr	tr	-	-	tr	tr	tr	tr
***n*-Dodecanal	24.96	1389	ND1	1389	0.09	tr	0.13	tr	0.05	0.05	tr	tr
***n*-Tetradecane	25.06	1392	1392	1400	tr	0.08	-	-	tr	-	-	tr
**Cyperene	25.11	1394	1402	1398	tr	-	-	-	-	-	-	-
**Sesquithujene	25.15	1395	1399	1411	-	-	-	-	-	-	-	-
***cis*-*α*-Bergamotene	25.37	1400	1409	1409	-	-	-	-	-	-	-	-
cis-*β*-Caryophyllene	25.39	1401	1409	1409	1.27	0.62	0.13	0.44	0.21	0.34	0.40	0.44

Suplement 2D.

Component	RT	KI_{EXP}	KI_{MF}	KI_{NIST}	PR-NW1	PR-NW1	PR-ŚL1	PR-ŚL2	PR-SZ1	PR-SZ2	PR-SZ3	PR-SZ4
*5,5-Dimethyl-4-(3-methyl-1,3-butadienyl)-1-oxaspiro[2.5]octane	25.46	1411	ND1	ND1	2.56	1.08	1.00	1.39	1.14	0.66	0.19	0.99
β-Copaene	25.50	1414	1430	1426	tr	tr	-	-	tr	tr	tr	tr
α-Guaiene	25.73	1430	1434	1444	1.27	0.38	0.22	0.33	0.18	0.41	0.38	0.24
Isogermacrene D	25.89	1440	1445	1437	0.34	0.10	0.08	0.12	0.13	0.10	0.05	0.08
α-Humulene	25.94	1444	1455	1447	0.41	0.13	tr	0.07	0.06	0.11	0.15	0.08
cis-*β*-Farnesene	25.99	1447	1446	1448	-	-	-	-	-	-	-	-
allo-Aromadendrene	26.05	1451	1462	1460	0.63	0.12	0.07	0.13	0.16	0.16	0.24	0.18
**Prenyl benzoate	26.08	1453	1450	1477	tr	tr	tr	-	tr	-	-	-
**(*cis*, *cis*)-*α*-Farnesene	26.13	1456	1460	1469	-	-	-	-	-	-	-	-
7aH,10bH-Cadina-1(6),4-diene	26.20	1461	1460	1468	0.14	tr	tr	0.05	tr	tr	0.06	-
γ-Muurolene	26.23	1466	1474	1472	0.99	0.29	0.21	0.30	0.28	0.32	0.34	0.34
α-Amorphene	26.29	1471	1477	1465	0.25	0.12	0.08	0.15	0.42	0.17	0.40	0.15
ar-Curcumene	26.29	1467	1473	1473	-	-	-	-	-	-	-	-
γ-Curcumene	26.33	1469	1475	1478	-	-	-	-	-	-	-	-
β-Eudesmene	26.42	1475	1486	1482	0.79	0.31	0.24	0.38	0.15	0.26	0.25	0.17
Germacrene D	26.47	1479	1479	1477	0.16	tr	-	-	0.18	0.15	0.10	0.07
epi-Cubebol	26.52	1484	1490	1489	0.23	0.05	0.07	0.14	0.37	0.31	0.43	0.19
α-Selinene	26.58	1486	1494	1491	0.67	0.30	0.23	0.26	-	-	-	-
α-Muurolene	26.64	1488	1496	1496	1.01	0.29	0.21	0.36	0.53	0.35	0.51	0.37
α-Bulnesene	26.72	1494	1503	1502	0.28	0.10	tr	0.07	0.11	0.08	0.16	0.07
γ-Cadinene	26.81	1500	1507	1507	1.97	0.50	0.35	0.64	0.53	0.63	0.78	0.64
trans and *cis*-Calamene	26.85	1501	1520	1510	1.45	0.43	0.25	0.66	0.51	0.51	0.61	0.63
Methyl dodecanoate	26.87	1505	ND1	1508	0.19	0.30	0.43	0.46	0.21	0.32	0.18	-
δ-Cadinene	26.92	1511	1526	1516	2.42	0.71	0.31	0.66	0.72	0.78	1.07	0.79
*4,5,9,10-Dehydroisolongifolene	27.03	1521	ND1	ND1	0.44	0.13	0.14	0.11	0.83	0.23	0.66	0.26
α-Calacorene	27.09	1524	1527	1532	0.52	0.10	0.10	0.15	0.32	0.23	0.38	0.17
α-Cadinene	27.10	1526	1530	1528	0.26	0.09	0.43	0.08	0.05	-	0.08	0.10

Suplement 2E.

Component	RT	KI_{EXP}	KI_{MF}	KI_{NIST}	PR-NW1	PR-NW1	PR-ŚL1	PR-ŚL2	PR-SZ1	PR-SZ2	PR-SZ3	PR-SZ4
***trans-Z-α*-Bisabolene epoxide**	**27.15**	**1532**	**ND1**	**1540**	0.64	0.39	tr	0.57	0.28	0.31	0.15	0.17
***trans*-Nerolidol**	**27.29**	**1545**	**1553**	**1540**	0.40	0.13	0.16	0.24	0.38	0.22	0.36	0.23
***α*-Cedrene oxide**	**27.44**	**1562**	**1571**	**ND1**	1.03	0.53	0.59	0.74	0.56	0.55	0.49	0.59
***Oxygenated sesquiterpenoid**	**27.51**	**1565**	**ND1**	**ND1**	0.54	0.27	0.23	0.36	0.49	0.32	0.59	0.20
**Caryophyllene oxide**	**27.55**	**1569**	**1552**	**1574**	1.96	1.36	0.75	0.50	1.24	1.24	1.44	0.67
**(-)-Gleenol**	**27.57**	**1570**	**1574**	**1577**	0.49	0.27	0.36	0.96	-	-	-	-
***α*-Guaiol**	**27.72**	**1584**	**1593**	**1589**	-	-	-	-	1.31	0.15	0.87	0.29
***trans-Z-α*-Bisabolene epoxide**	**27.74**	**1581**	**ND1**	**1586**	0.37	0.14	0.06	0.18	-	-	-	-
****Isoaromadendrene epoxide**	**27.81**	**1588**	**ND1**	**1590**	0.17	tr	0.06	0.10	-	-	-	-
**Humulene epoxide (II)**	**27.85**	**1596**	**1602**	**1599**	1.49	1.22	1.05	1.33	1.87	0.95	1.24	0.71
**Neointermedeol**	**27.91**	**1601**	**ND1**	**1601**	0.19	0.17	0.20	0.26	-	-	-	-
**Di-epi-1,10-cubenol**	**27.93**	**1603**	**1615**	**1609**	0.69	0.28	0.18	0.37	-	-	-	-
**Muurola-4,10(14)-dien-1-β-ol**	**27.98**	**1608**	**ND1**	**1609**	0.28	0.14	0.11	0.18	-	-	-	-
**Epicubenol**	**28.03**	**1613**	**ND1**	**1617**	1.09	3.03	1.11	0.53	0.46	0.31	0.47	0.31
**10-epi-γ-Eudesmol**	**28.04**	**1615**	**1609**	**1615**	-	-	-	-	2.24	0.92	2.87	1.18
**Caryophylla-4(12),8(13)-dien-5*α*-ol**	**28.08**	**1619**	**ND1**	**1623**	1.46	1.19	1.97	1.63	5.04	1.26	-	0.10
**γ-Eudesmol**	**28.11**	**1621**	**1618**	**1618**	0.53	3.03	2.37	1.14	8.77	2.84	8.71	3.56
**τ-Cadinol**	**28.15**	**1626**	**1633**	**1631**	4.58	2.89	2.15	3.12	3.92	2.27	4.52	2.68
**δ-Cadinol**	**28.21**	**1632**	**ND1**	**1627**	1.58	1.08	1.24	2.14	1.54	1.72	1.21	1.47
**β-Eudesmol**	**28.26**	**1636**	**1641**	**1636**	0.32	1.59	1.82	0.33	9.50	2.58	8.49	2.60
***α*-Cadinol**	**28.28**	**1642**	**1643**	**1643**	3.10	3.32	0.96	3.43	-	-	-	-
***α*-Eudesmol**	**28.30**	**1640**	**1659**	**1654**	-	0.82	4.38	1.49	9.29	4.10	9.60	4.25
***Ledene oxide (II)**	**28.35**	**1645**	**ND1**	**1646**	0.25	0.29	0.21	0.30	1.25	0.34	-	0.20
**Bulnesol**	**28.43**	**1654**	**1665**	**1655**	-	-	-	-	2.54	1.15	1.30	0.90
**Cadalene**	**28.45**	**1655**	**1659**	**1660**	0.33	0.53	-	-	-	-	-	-
***Eudesma-3,11-dien-8-one**	**28.53**	**1664**	**1666**	**1646**	0.72	0.88	1.12	1.59	0.48	0.36	0.23	0.34
***Eudesma-4(15),7-dien-1β -ol**	**28.64**	**1675**	**1671**	**1685**	0.22	0.05	0.08	0.15	-	-	-	-

Suplement 2F.

Component	RT	KI_{EXP}	KI_{MF}	KI_{NIST}	PR-NW1	PR-NW1	PR-ŚL1	PR-ŚL2	PR-SZ1	PR-SZ2	PR-SZ3	PR-SZ4
*Oxygenated sesquiterpenoid	28.69	1679	ND1	ND1	0.07	tr	-	-	0.20	0.08	0.16	0.09
*Oxygenated sesquiterpenoid	28.82	1692	ND1	ND1	0.11	-	-	-	-	-	-	-
*6α-Hydroxygermacra-1(10),4-diene	28.84	1694	ND1	ND1	0.08	-	-	-	-	-	-	-
n-Heptadecane	28.87	1697	1700	1700	0.08	0.11	0.08	0.19	0.12	0.12	0.11	0.12
Methyl tetradecanoate	28.94	1704	ND1	1708	0.13	0.09	0.15	tr	0.15	0.30	0.16	0.09
Benzyl benzoate	29.10	1726	1730	1732	11.89	28.61	26.43	23.57	2.65	6.29	7.38	5.80
γ-Costol	29.20	1737	1730	ND1	1.82	0.50	0.77	0.81	0.77	0.39	0.20	0.48
*15-Hydroxy-α-muurolene	29.22	1740	ND1	1757	0.64	0.18	0.24	0.35	0.19	0.11	0.12	0.12
β-Costol	29.32	1753	1754	1769	0.85	0.21	0.44	0.26	0.42	0.19	0.20	0.21
α-Costol	29.48	1772	1761	1757	6.24	1.32	2.50	2.81	4.01	1.38	0.57	1.97
Selina-4α,11-diol	29.60	1787	ND1	1780	-	-	-	-	0.25	0.05	0.05	tr
*Oxygenated sesquiterpenoid	29.69	1797	ND1	ND1	-	tr	0.12	0.22	0.10	0.11	0.08	0.08
Phenylethyl benzoate	29.83	1813	1815	1828	0.09	-	0.05	-	0.05	-	tr	tr
Benzyl salicylate	29.98	1831	1847	1836	5.42	0.55	3.33	3.75	1.09	2.04	2.43	1.42
*Methyl 7-hexadecenoate	30.19	1869	ND1	1888	0.26	-	0.12	0.11	0.22	0.21	0.33	0.41
*Aliphatic alcohol	30.41	1900	ND1	ND1	0.12	0.26	0.41	0.88	-	-	-	-
Methyl 4,7,10,13-hexadecatetraenote	30.46	1906	ND1	ND1	0.26	1.03	0.51	0.75	0.80	1.08	0.50	0.26
*Aliphatic component	31.17	2004	ND1	ND1	0.42	-	0.25	-	-	-	-	-
**cis*-Benzyl cinnamate	31.32	2029	ND1	1959	-	-	0.24	0.53	0.10	0.07	0.08	0.11
trans-Benzyl cinnamate	31.51	2059	2023	ND1	0.68	3.91	5.52	8.97	1.80	3.28	2.72	4.62
*Aliphatic ester	31.63	2077	ND1	ND1	-	0.23	0.47	-	0.31	0.34	0.27	0.18
n-Heneicosane	31.74	2094	2100	2100	0.17	0.47	0.56	-	0.88	1.57	1.01	0.69
*Benzyl dodecanoate	31.95	2128	ND1	2097	-	0.14	tr	0.16	-	-	0.08	tr
*Aliphatic component	32.33	2189	ND1	ND1	-	tr	0.05	0.09	0.07	0.05	0.05	0.06
*Aliphatic component	32.77	2265	ND1	ND1	tr	0.10	0.12	0.22	0.19	0.13	0.15	0.15
Tricosane	32.91	2290	2301	2300	0.29	1.32	1.38	2.84	1.49	1.41	1.70	1.60
*Aliphatic component	33.47	ND	ND1	ND1	-	tr	-	-	0.07	0.05	0.06	0.05

Suplement 2G.

Component	RT	KI_{EXP}	KI_{MF}	KI_{NIST}	PR-NW1	PR-NW1	PR-ŚL1	PR-ŚL2	PR-SZ1	PR-SZ2	PR-SZ3	PR-SZ4
***Aliphatic component**	**33.85**	**ND**	**ND1**	**ND1**	-	0.11	0.05	0.10	0.14	0.08	0.10	0.07
***Aliphatic component**	**34.00**	**ND**	**ND1**	**ND1**	-	0.91	0.09	0.20	0.89	1.05	1.10	0.95
***Aliphatic component**	**34.07**	**ND**	**ND1**	**ND1**	0.09	0.14	1.21	1.81	0.11	0.32	0.29	0.10
***Aliphatic component**	**34.50**	**ND**	**ND1**	**ND1**	-	0.07	0.12	0.32	0.05	0.06	0.06	0.05

Legend

* – amount of single component calculated as percent (%) of whole GC-MS chromatogram area

** – possible xenobiotic

tr – trace concentration of component <0,05%

"–" - substance under detection condition

KI_{EXP} – Experimental retention index

KI_{MF} – Retention index according to Terpenoid Library **[1]**

KI_{NIST} – Retention index according to NIST Chemistry WebBook **[2]**

ND – Retention Indices was not determined

ND1 – No data for DB-1 capillary column type

Literature:

1. König, W.A.; Joulain, D.; Grasse, R.S.; Hochmuth, D.H. Terpenoids and related constituents of essential oils, Hochmuth Scientific Consulting. Aviable on: http://massfinder.com/wiki/Terpenoids_Library. (accessed on: 15th May 2017)
2. National Institute of Standard and Technology. NIST Chemistry WebBook. Old York Road, Ringoes, USA, National Institute of Standards and Technology. Aviable on: http://webbook.nist.gov/chemistry/ (accessed on: 15th May 2017)

Suplement 3A. Full result of GC-MS analysis of Canadian, German and Polish propolis essential oils

Component	RT	KI_{EXP}	KI_{MF}	KI_{NIST}	PR-LS1	PR-LS2	PR-LS3	PR-LS4	PR-LS5	PR-LS6	PR-S1	PR-S2	PR-MR	PR-NSW	PR-GR	PR-CN
***trans*-2-Hexenal	4.15	826	832	827	-	-	-	-	-	tr	-	-	-	-	-	-
2-Methylbutanoic acid	4.35	836	ND1	839	tr	0.11	0.10	0.21	0.19	-	0.06	tr	0.09	0.18	0.05	tr
n-Hexanol	4.72	852	837	854	-	-	-	-	-	0.15	-	-	-	-	-	-
***p*-Xylene	4.81	856	ND1	860	-	-	-	-	-	tr	-	-	-	-	-	-
*3-methyl-3-buten-1-ol acetate	5.02	865	ND1	862	-	-	-	-	-	-	-	-	-	-	tr	-
Styrene	5.21	872	ND1	878	-	-	-	-	-	-	-	-	-	-	-	tr
***o*-Xylene	5.39	879	ND1	881	-	0.05	-	tr	-	-	-	-	tr	tr	-	-
**Prenyl acetate	6.00	901	902	902	-	-	-	-	-	-	-	-	-	-	tr	-
trans-2-Methyl-2-butenoic acid (Tiglic acid)	6.15	907	ND1	946	0.10	0.16	0.20	0.15	0.14	0.76	0.10	0.06	0.16	0.22	tr	0.17
Benzaldehyde	6.76	927	941	933	0.08	0.24	0.37	0.12	0.20	0.30	0.23	0.09	0.34	0.12	0.15	0.09
α-Pinene	6.79	930	936	933	-	-	-	tr	tr	-	-	-	-	-	-	-
Phenol	7.77	957	ND1	957	-	-	-	-	-	-	-	-	-	-	0.11	-
**6-Methyl-5-hepten-2-one	8.01	965	ND1	964	-	tr	tr	tr	tr	-	tr	-	-	-	-	0.10
n-Hexanoic acid	8.26	971	ND1	982	0.50	0.20	0.19	0.07	0.14	0.17	0.36	0.23	0.31	0.35	-	0.21
n-Octanal	8.60	980	981	982	tr	0.15	0.20	tr	0.07	0.15	0.11	tr	tr	0.08	-	tr
**3-*δ*-Carene	9.47	1001	1000	998	-	tr	tr	tr	tr	tr	-	-	tr	-	-	-
Benzyl alcohol	9.49	1001	1006	1012	2.68	6.39	6.19	0.87	1.46	4.93	6.48	3.23	2.10	4.31	1.13	1.39
**Salicylaldehyde	9.57	1005	1012	1029	-	tr	tr	tr	tr	-	tr	tr	tr	0.05	-	-
***m*-Cymene	9.66	1007	1013	1010	-	-	-	tr	tr	tr	-	-	tr	-	-	-
Eucalyptol (1,8-Cyneole)	10.02	1016	1024	1022	tr	0.05	tr	0.16	0.14	0.05	tr	tr	tr	tr	tr	tr
Limonene	10.03	1017	1025	1023	-	tr	tr	tr	tr	tr	tr	-	2.14	tr	tr	-
Acetophenone	10.56	1030	1036	1042	0.18	-	-	-	-	0.15	-	-	-	-	0.26	0.19
**Benzyl formate	11.15	1046	1060	1046	tr	0.06	tr	-	tr	tr	0.11	tr	tr	0.06	-	-
trans-Linalool oxide (furanoid)	11.61	1055	1058	1064	-	0.11	0.12	0.06	0.10	0.24	0.12	tr	0.09	0.11	tr	tr
o-Guaiacol	11.66	1058	1072	1063	-	-	-	-	-	-	-	-	-	-	0.19	-
Methyl benzoate	12.01	1065	1072	1072	-	0.05	0.11	tr	0.08	tr	0.08	tr	0.09	0.09	tr	0.08
cis-Linalool oxide (furanoid)	12.21	1065	1072	1072	0.09	0.19	0.17	0.10	0.16	0.15	0.22	0.05	0.14	0.19	tr	tr

Suplement 3B.

Component	RT	KI_{EXP}	KI_{MF}	KI_{NIST}	PR-LS1	PR-LS2	PR-LS3	PR-LS4	PR-LS5	PR-LS6	PR-S1	PR-S2	PR-MR	PR-NSW	PR-GR	PR-CN
**6-Methyl-3,5-heptadien-2-one	12.47	1077	ND1	1076	tr	-	-	-	-	-	-	-	-	-	-	tr
2-Phenylethanol	12.65	1079	1081	1088	0.21	1.14	1.30	0.43	0.44	2.31	1.22	0.33	0.35	0.84	5.83	0.21
2-Nonanol	12.71	1082	1085	1089	0.08	0.25	0.51	0.14	0.27	0.32	0.36	0.13	0.17	0.31	-	0.11
Linalool	12.78	1082	1086	1086	0.27	0.07	0.13	0.18	0.21	0.23	0.13	tr	0.11	0.17	-	0.12
*Benzyl component	12.911	1086	ND1	ND1	-	-	-	-	-	-	-	-	-	-	0.13	-
*2-Metylobutyl-3-methylbutanoate	13.12	1090	ND1	1091	-	tr	0.06	tr	0.05	-	0.17	tr	0.08	0.05	-	-
**2-Ethenyl-1,1-dimethyl-3-methylene-cyclohexane	13.49	1098	ND1	ND1	tr	-	-	-	-	-	-	-	-	-	-	-
**Veratrole	13.91	1106	1117	1111	tr	-	-	-	-	-	-	-	-	-	-	-
**Camphor	14.09	1113	1123	1123	tr	tr	tr	-	tr	tr	-	-	tr	tr	tr	-
*2-Metyleno-acetophenone	14.19	1115	ND1	ND1	-	-	-	-	-	-	-	-	-	-	0.32	-
Benzenepropanal	14.26	1117	ND1	1130	0.07	0.21	0.43	0.08	0.16	0.52	0.65	0.07	tr	0.16	-	0.57
p-Vinylanisole	14.63	1126	1134	1152	tr	-	-	-	-	0.19	0.13	tr	0.10	0.06	-	-
**2-Fenylobutadien	14.66	1127	ND1	ND1	tr	-	-	-	-	-	-	-	-	-	-	0.06
Benzyl acetate	14.78	1130	1134	1139	tr	0.25	0.17	tr	tr	0.68	0.28	tr	0.06	0.26	0.23	-
***cis*-2-Nonenal	14.94	1134	ND1	1135	-	tr	tr	tr	tr	tr	tr	tr	tr	tr	-	-
*Benzyl component	15.14	1138	ND1	ND1	-	-	-	-	-	-	-	-	-	-	0.16	-
o-Ethylphenol	15.21	1140	ND1	1139	-	-	-	-	-	-	-	-	-	-	0.13	-
tran/cis-Linalool oxide (Pyranoid)	15.45	1144	1144	1164	0.16	-	-	-	-	-	-	-	-	-	tr	-
**Terpinen-4-ol	15.88	1155	1164	1160	-	-	-	-	-	-	-	-	-	-	tr	-
Benzoic acid	16.26	1138	1160	1161	13.10	12.14	12.38	5.23	3.75	1.57	12.79	30.24	16.15	12.94	-	47.06
Methyl salicylate	16.29	1163	1171	1175	-	-	0.11	0.22	0.18	0.18	0.16	-	0.10	0.43	0.08	0.06
a-Terpineol	16.45	1166	1176	1175	-	-	-	-	0.19	0.06	0.24	-	-	-	tr	-
**Verbenone	16.77	1175	1183	1170	-	-	-	-	-	-	-	-	-	-	tr	-
*Alipatic component	17.03	1180	ND1	ND1	-	-	-	-	-	0.31	-	-	-	-	-	-
n-Decenal	17.17	1183	ND1	1240	0.47	0.62	1.31	0.15	0.33	0.71	1.01	0.36	0.37	0.56	-	0.16
**β-Cyclocitral	17.48	1187	1195	1173	-	-	-	-	-	-	-	-	-	-	0.06	-
2,3-Dihydrobenzofuran	17.53	1188	ND1	1190	0.82	0.58	1.04	0.20	0.29	0.30	0.85	1.14	0.64	0.97	tr	0.62

Suplement 3C.

Component	RT	KI_{EXP}	KI_{MF}	KI_{NIST}	PR-LS1	PR-LS2	PR-LS3	PR-LS4	PR-LS5	PR-LS6	PR-S1	PR-S2	PR-MR	PR-NSW	PR-GR	PR-CN
**3-Phenylpropanol**	**17.57**	**1191**	**ND1**	**1202**	-	-	-	-	-	-	-	-	-	-	0.21	-
***p*-Anisaldehyde**	**18.24**	**1206**	**1218**	**1224**	-	tr	tr	tr	tr	-	0.05	tr	tr	tr	-	-
****Benzyl acrylate**	**18.53**	**1213**	**ND1**	**ND1**	-	tr	tr	tr	tr	tr	0.08	tr	tr	tr	-	-
**2-Phenylethyl acetate**	**18.83**	**1221**	**1235**	**1228**	-	0.19	0.37	0.10	0.23	0.28	0.38	0.20	0.23	0.19	1.38	-
***cis*-Cinnamaldehyde**	**18.94**	**1223**	**1234**	**1237**	0.05	0.43	0.28	0.12	0.12	0.23	0.51	0.06	0.12	0.13	-	0.21
**Allyl benzoate**	**19.00**	**1224**	**ND1**	**1239**	0.23	-	-	-	-	-	-	-	-	-	-	0.12
*****cis*-Geraniol**	**19.43**	**1233**	**1235**	**1237**	tr	-	-	-	-	0.12	-	-	-	0.05	tr	-
*****n*-Propyl benzoate**	**19.56**	**1238**	**1275**	**1275**	-	-	-	-	-	-	-	-	-	-	tr	-
***p*-Ethylguaiacol**	**19.98**	**1247**	**ND1**	**1252**	-	-	-	-	-	-	-	-	-	-	0.18	-
***n*-Nonanoic acid**	**20.39**	**1257**	**1263**	**1272**	0.13	0.11	0.16	tr	tr	0.06	0.20	0.05	tr	0.12	-	-
**Cinnamic alcohol**	**20.86**	**1269**	**1274**	**1275**	0.10	0.29	0.42	0.08	0.11	0.44	0.52	0.11	0.06	0.13	2.36	1.59
****p*-Vinylguaiacol**	**21.31**	**1277**	**1311**	**1295**	5.98	2.04	3.18	0.55	1.17	0.53	2.00	8.26	6.41	7.70	0.11	1.19
****Phenacyl alcohol**	**21.41**	**1279**	**ND1**	**ND1**	-	-	-	-	-	tr	-	-	-	-	-	-
**2-Hydroxy-5-methylacetophenone**	**21.90**	**1290**	**ND1**	**1285**	-	-	-	-	-	0.20	-	-	-	-	0.06	0.12
****Methyl o-methoxybenzoate**	**21.97**	**1291**	**1300**	**1291**	tr	-	-	-	-	-	-	-	-	-	-	tr
****Methyl geramate**	**22.26**	**1297**	**1306**	**1308**	-	-	-	-	-	0.05	-	-	-	-	-	-
**Methyl benzoylformate**	**22.73**	**1312**	**1313**	**ND1**	tr	0.35	0.67	0.43	0.44	-	1.02	1.21	1.37	0.25	-	-
***Ethyl dihydrocinnamate**	**22.74**	**1312**	**ND1**	**1320**	-	-	-	-	-	-	-	-	-	-	0.11	-
***cis*-4-Phenyl-3-buten-2-one**	**22.81**	**1315**	**ND1**	**1323**	tr	-	-	-	-	-	-	-	-	-	0.19	0.09
**2-Phenylethyl propanoate**	**22.87**	**1317**	**ND1**	**1325**	-	-	-	-	-	-	-	-	-	-	0.31	-
****8,9-Dehydro-cycloisolongifolene**	**22.98**	**1325**	**1320**	**ND1**	tr	-	-	-	-	-	-	-	-	-	-	-
**Eugenol**	**23.26**	**1331**	**1331**	**1335**	0.25	0.13	0.20	0.08	0.11	0.23	0.20	0.12	0.15	0.15	0.28	0.39
**3,4-Dimethoxystyrene**	**23.44**	**1337**	**1337**	**1354**	0.06	0.15	0.16	0.07	0.08	0.12	0.17	tr	tr	0.27	0.19	-
**3-Phenyl-1-propyl acetate**	**23.53**	**1340**	**ND1**	**1347**	-	-	-	-	-	-	-	-	-	-	0.07	-
***Aliphatic component**	**23.55**	**1341**	**ND1**	**ND1**	-	0.09	0.20	-	-	0.05	0.08	0.07	tr	0.06	-	-
****Methyl cinnamate**	**23.78**	**1349**	**ND1**	**1348**	-	-	-	-	-	-	-	-	-	-	0.07	-
***α*-Cubebene**	**23.79**	**1349**	**1355**	**1365**	tr	-	-	0.06	0.10	-	tr	-	-	-	-	-

Suplement 3D.

Component	RT	KI_{EXP}	KI_{MF}	KI_{NIST}	PR-LS1	PR-LS2	PR-LS3	PR-LS4	PR-LS5	PR-LS6	PR-S1	PR-S2	PR-MR	PR-NSW	PR-GR	PR-CN
Vanillin	23.93	1354	1355	1360	0.34	1.94	2.04	0.51	0.60	0.44	1.91	2.59	1.34	1.08	-	0.43
Unidentified	24.00	1357	ND1	ND1	-	-	-	-	-	-	-	-	-	-	0.18	-
***o*-Vanillin	24.15	1362	ND1	ND1	0.06	-	-	-	-	-	-	-	-	-	-	-
Unidentified	24.27	1366	ND1	ND1	-	-	-	-	-	-	-	-	-	-	0.13	-
α-Ylanglene	24.40	1370	1376	1370	-	tr	-	0.20	0.21	0.09	0.09	-	tr	0.06	-	tr
α-Copaene	24.52	1374	1379	1376	0.11	0.13	0.10	0.50	0.57	0.37	0.26	0.09	0.12	0.17	-	0.07
β-Patchoulene	24.52	1374	ND1	1381	-	-	-	-	-	-	-	-	-	-	0.22	-
β-Bourbonene	24.67	1379	1386	1382	-	-	tr	0.27	0.31	tr	tr	-	tr	tr	-	-
β-Cubebene	24.76	1382	1378	ND1	-	tr	tr	0.05	0.05	tr	0.17	tr	tr	-	-	-
**7-*epi*-Sesquithujene	24.84	1385	1387	1385	-	-	-	-	-	-	-	-	-	-	-	tr
Methyloeugenol	24.96	1389	ND1	1372	-	-	-	-	-	-	-	-	-	-	0.15	-
***n*-Dodecanal	24.96	1389	ND1	1389	tr	-	-	-	-	0.09	-	-	-	-	-	tr
((Sativene	24.97	1389	1394	ND1	-	-	-	-	-	-	-	-	-	-	0.09	-
α-Cedrene	25.20	1397	1413	1418	-	-	-	-	-	-	-	-	-	-	0.25	-
Cinnamic acid	25.32	1401	ND1	1394	-	-	-	-	-	-	-	-	-	-	-	0.84
cis-*α*-Bergamotene	25.37	1400	1409	1409	-	-	-	-	-	-	-	-	-	-	1.28	-
cis-*β*-Caryophyllene	25.39	1401	1409	1409	-	tr	0.09	0.43	0.43	0.11	0.16	0.07	0.25	0.15	-	-
*5,5-Dimethyl-4-(3-methyl-1,3-butadienyl)-1-oxaspiro[2.5]octane	25.46	1411	ND1	ND1	2.95	1.79	-	-	-	-	-	-	-	1.30	-	-
2-Methylbutyl benzoate	25.33	1402	1419	1422	-	tr	0.08	0.29	0.31	-	0.37	tr	tr	tr	-	-
*Sesquiterpene hydrocarbon	25.35	1403	ND1	ND1	-	-	-	-	-	-	-	-	-	-	-	0.63
3-Methylbut-3-enyl benzoate	25.41	1408	ND1	1426	-	-	-	-	-	0.08	-	-	-	-	-	-
***β*-Copaene	25.50	1414	1430	1426	-	-	-	0.07	0.07	tr	tr	tr	tr	-	-	-
trans-*α*-Bergamoten	25.70	1427	1434	1443	-	-	-	-	-	tr	-	-	-	-	-	0.37
α-Guaiene	25.73	1430	1434	1444	-	-	0.47	0.67	-	0.11	0.30	0.41	0.32	0.40	2.21	-
*Sesquiterpene hydrocarbon	25.77	1432	ND1	ND1	-	-	-	-	-	-	-	-	-	-	0.12	-
Guaia-6,9-diene	25.86	1438	1448	1443	-	0.08	tr	0.22	0.23	-	-	tr	tr	-	-	-
Isogermacrene D	25.89	1440	1445	1437	-	tr	tr	0.08	0.10	tr	-	-	-	-	-	-

Suplement 3E.

Component	RT	KI_{EXP}	KI_{MF}	KI_{NIST}	PR-LS1	PR-LS2	PR-LS3	PR-LS4	PR-LS5	PR-LS6	PR-S1	PR-S2	PR-MR	PR-NSW	PR-GR	PR-CN
**α-Humulene**	**25.94**	**1444**	**1455**	**1447**	0.32	0.05	0.05	0.20	0.20	0.06	-	tr	0.11	0.28	0.36	tr
***cis*-β-Farnesene**	**25.99**	**1447**	**1446**	**1448**	-	-	-	-	-	-	-	-	-	-	-	0.19
***allo*-Aromadendrene**	**26.05**	**1451**	**1462**	**1460**	0.42	0.16	0.14	0.46	0.58	0.43	0.23	0.07	0.14	0.28	1.35	0.08
**Prenzl benzoate**	**26.08**	**1453**	**1450**	**1477**	-	-	0.65	3.29	3.26	tr	3.85	0.36	-	-	-	-
**(*cis, cis*)-α-Farnesene**	**26.13**	**1456**	**1460**	**1469**	tr	-	-	-	-	-	-	-	-	-	1.77	0.20
****Phenethyl 2-methylbutyrate**	**26.20**	**1461**	**ND1**	**1469**	-	-	-	-	-	0.06	-	-	-	-	-	-
****7aH,10bH-Cadina-1(6),4-diene**	**26.20**	**1461**	**1460**	**1468**	-	0.05	-	0.08	0.10	0.07	-	tr	tr	0.05	-	-
**γ-Muurolene**	**26.23**	**1466**	**1474**	**1472**	0.37	0.15	0.19	0.54	0.62	0.56	0.30	0.10	0.19	0.31	-	-
**7-*epi*-α-Selinene**	**26.24**	**1463**	**ND1**	**1473**	-	-	-	-	-	-	-	-	-	-	1.80	-
**α-Amorphene**	**26.29**	**1471**	**1477**	**1465**	0.37	0.08	-	0.62	0.52	0.15	-	-	0.11	0.15	-	-
***ar*-Curcumene**	**26.29**	**1467**	**1473**	**1473**	-	-	-	-	-	-	-	-	-	-	11.10	1.41
**γ-Curcumene**	**26.33**	**1469**	**1475**	**1478**	-	-	-	-	-	-	-	-	-	-	6.77	0.84
**β-Eudesmene**	**26.42**	**1475**	**1486**	**1482**	0.36	0.18	0.28	0.39	0.42	0.25	0.43	0.13	0.28	0.20	1.72	0.17
**Germacrene D**	**26.47**	**1479**	**1479**	**1477**	-	0.09	0.07	0.34	0.34	-	0.13	tr	-	-	2.31	-
**γ-Amorphene**	**26.53**	**1482**	**1492**	**1491**	-	-	-	-	-	-	-	-	-	-	-	tr
**α-Selinene**	**26.58**	**1486**	**1494**	**1491**	0.30	0.14	0.24	0.67	0.70	0.34	0.15	0.14	0.28	0.29	2.33	0.36
**α-Muurolene**	**26.64**	**1488**	**1496**	**1496**	0.36	0.19	0.22	0.57	0.57	0.90	0.15	0.09	0.22	0.48	-	0.13
**α-Farnesene**	**26.70**	**1493**	**1498**	**1496**	-	-	-	-	-	-	-	-	-	-	-	1.74
**α-Bulnesene**	**26.72**	**1494**	**1503**	**1502**	0.08	0.36	0.15	0.94	0.99	-	0.31	tr	0.11	0.10	2.09	-
**γ-Cadinene**	**26.81**	**1500**	**1507**	**1507**	0.47	0.46	0.37	0.91	0.93	1.69	0.74	0.19	0.35	0.83	5.17	-
**β-Curcumene**	**26.83**	**1503**	**1503**	**ND1**	-	-	-	-	-	-	-	-	-	-	0.92	0.82
***trans* and *cis*-Calamene**	**26.85**	**1501**	**1520**	**1510**	0.43	0.22	0.24	0.81	0.93	1.26	0.33	0.12	0.24	0.64	1.02	0.12
***Methyl dodecanoate**	**26.87**	**1506**	**ND1**	**ND1**	0.16	-	-	-	-	-	-	-	-	-	-	-
**δ-Cadinene**	**26.92**	**1511**	**1526**	**1516**	0.67	0.50	0.55	1.62	2.17	2.33	0.55	0.29	0.41	1.49	0.86	0.44
***α-Copaen-11-ol**	**26.99**	**1517**	**ND1**	**ND1**	-	-	-	-	-	0.93	-	-	-	-	2.66	0.15
**Cadina-1,4-diene**	**27.03**	**1522**	**1523**	**1525**	-	tr	0.05	0.16	0.17	0.08	0.10	tr	0.05	0.06	0.81	0.11

Suplement 3F.

Component	RT	KI$_{EXP}$	KI$_{MF}$	KI$_{NIST}$	PR-LS1	PR-LS2	PR-LS3	PR-LS4	PR-LS5	PR-LS6	PR-S1	PR-S2	PR-MR	PR-NSW	PR-GR	PR-CN
*4,5,9,10-Dehydroisolongifolene	27.03	1521	ND1	ND1	0.29	0.32	0.26	1.63	1.50	-	0.23	0.06	0.15	0.32	-	-
α-Calacorene	27.09	1524	1527	1532	0.25	0.11	0.12	0.68	0.74	0.38	0.24	0.10	0.13	0.27	-	-
α-Cadinene	27.10	1526	1530	1528	0.07	-	-	-	-	-	-	-	-	-	-	0.14
trans-γ-Bisabolene	27.13	1531	1521	1525	-	-	-	-	-	-	-	-	-	-	0.93	0.11
**trans*-*Z*-α-Bisabolene epoxide	27.15	1532	ND1	1540	0.72	1.03	0.91	0.25	0.27	-	0.47	0.21	0.18	0.68	-	-
Selina-3,7(11)-diene	27.17	1534	1542	1549	-	-	-	-	-	-	-	-	-	-	0.56	tr
Elemol	27.29	1545	1541	1537	-	-	-	-	-	-	-	-	-	-	-	-
trans-Nerolidol	27.29	1545	1553	1540	-	0.29	-	0.15	0.19	0.06	0.17	0.30	0.12	0.45	-	0.26
Germacrene B	27.32	1548	1552	1549	-	-	-	-	-	-	-	-	-	-	0.81	-
α-Cedrene oxide	27.44	1562	1571	ND1	0.12	-	-	-	-	-	-	-	-	-	-	-
(+)-Spathulenol	27.45	1560	1572	1568	-	0.43	0.24	0.24	0.26	-	0.17	0.25	0.20	0.38	-	-
*Oxygenated sesquiterpenoid	27.51	1565	ND1	ND1	0.71	0.68	1.29	0.38	0.36	-	4.55	0.22	1.26	-	-	-
Caryophyllene oxide	27.55	1569	1552	1574	0.98	1.04	1.29	2.29	2.12	0.52	5.60	0.52	0.48	1.22	-	-
*(-)-Spathulenol	27.63	1576	ND1	1577	-	-	-	-	-	-	-	-	-	-	0.24	-
α-Guaiol	27.72	1584	1593	1589	-	0.10	0.27	2.44	2.08	0.36	-	-	0.19	-	4.96	0.27
**trans*-*Z*-α-Bisabolene epoxide	27.74	1581	ND1	1586	1.45	0.10	0.09	0.24	0.10	0.39	0.13	0.08	tr	0.14	-	0.06
*Isoaromadendrene epoxide	27.81	1588	ND1	1590	0.23	-	-	-	-	0.23	-	-	-	-	-	-
Humulene epoxide (II)	27.85	1596	1602	1599	1.53	1.19	0.49	1.86	1.21	0.42	0.38	0.78	0.59	0.63	-	-
*Rosifoliol	27.89	1599	ND1	1600	-	-	-	-	-	-	-	-	-	-	-	0.18
Neointermedeol	27.91	1601	ND1	1601	0.17	-	-	-	-	-	-	-	-	-	0.10	tr
Di-epi-1,10-cubenol	27.93	1603	1615	1609	0.49	-	-	-	-	-	-	-	-	-	-	-
Muurola-4,10(14)-dien-1-β-ol	27.98	1608	ND1	1609	0.19	-	-	-	-	-	-	-	-	-	-	-
Epicubenol	28.03	1613	ND1	1617	-	-	0.35	-	0.48	0.68	0.29	0.25	0.21	0.69	-	0.13
10-epi-γ-Eudesmol	28.04	1615	1609	1615	-	-	-	-	-	-	-	-	-	-	-	1.80
Cubenol	28.06	1616	1630	1620	1.08	0.30	0.29	0.36	0.26	1.07	0.33	0.24	0.18	1.04	1.13	-
Caryophylla-4(12),8(13)-dien-5α-ol	28.08	1619	ND1	1623	2.17	-	-	-	-	-	-	-	-	-	-	-

Suplement 3G.

Component	RT	KI_{EXP}	KI_{MF}	KI_{NIST}	PR-LS1	PR-LS2	PR-LS3	PR-LS4	PR-LS5	PR-LS6	PR-S1	PR-S2	PR-MR	PR-NSW	PR-GR	PR-CN
**γ-Eudesmol**	**28.11**	**1621**	**1618**	**1618**	3.23	5.33	6.96	14.77	11.26	16.90	1.17	3.16	1.16	1.73	5.56	5.35
**τ-Cadinol**	**28.15**	**1626**	**1633**	**1631**	3.12	5.82	4.19	3.85	4.11	7.75	2.22	2.11	1.54	1.73	-	-
**Agarospirol**	**28.18**	**1628**	**1635**	**1631**	-	1.55	-	-	1.12	-	-	-	-	-	1.74	0.84
**Isospathulenol**	**28.19**	**1630**	**ND1**	**1628**	-	-	-	-	-	-	-	-	-	-	2.30	-
**δ-Cadinol**	**28.21**	**1632**	**ND1**	**1627**	2.84	1.14	0.81	0.63	0.64	1.03	0.62	0.38	0.29	7.27	-	-
**(+)-Intermedeol**	**28.25**	**1636**	**ND1**	**1645**	-	-	-	-	-	-	-	-	-	-	-	-
**β-Eudesmol**	**28.26**	**1636**	**1641**	**1636**	1.29	0.57	5.24	10.06	9.91	18.71	1.22	0.66	-	-	5.02	5.18
**α-Cadinol**	**28.28**	**1642**	**1643**	**1643**	2.20	-	-	-	-	-	-	-	2.41	6.10	-	-
**α-Eudesmol**	**28.30**	**1640**	**1659**	**1654**	3.46	9.60	10.59	16.78	10.92	17.79	3.08	4.59	0.50	1.89	6.63	6.72
***Ledene oxide (II)**	**28.35**	**1645**	**ND1**	**1646**	0.55	1.16	0.74	-	-	0.37	0.15	0.20	0.26	0.24	-	-
***Oxygenated sesquiterpenoid**	**28.39**	**1650**	**ND1**	**ND1**	-	-	-	-	-	0.36	-	-	-	-	-	-
**β-Bisabolol**	**28.42**	**1653**	**1659**	**ND1**	-	-	-	-	-	-	-	-	-	-	-	0.47
**Bulnesol**	**28.43**	**1654**	**1665**	**1655**	-	-	-	3.86	2.84	-	-	-	-	-	4.80	-
**Cadalene**	**28.45**	**1655**	**1659**	**1660**	-	-	-	-	-	0.20	0.16	0.13	-	0.19	-	-
***Eudesma-3,11-dien-8-one**	**28.53**	**1664**	**1666**	**1646**	2.01	1.56	0.75	-	-	-	0.30	0.52	0.36	0.63	-	-
**α-Bisabolol**	**28.58**	**1668**	**1673**	**1680**	-	1.47	-	1.27	1.11	-	-	-	0.10	-	2.55	1.83
***Eudesma-4(15),7-dien-1β -ol**	**28.64**	**1675**	**1671**	**1685**	0.87	0.11	0.08	0.21	0.15	0.08	-	-	-	-	-	-
***Eudesm-7(11)-en-4-ol**	**28.66**	**1677**	**1676**	**1682**	-	-	-	-	-	-	-	-	-	-	-	0.12
***Oxygenated sesquiterpenoid**	**28.69**	**1679**	**ND1**	**ND1**	0.29	1.08	0.33	0.30	0.26	0.09	-	0.44	0.14	0.38	0.25	-
**Germacra-4(15),5,10(14)-trien-1α-ol**	**28.70**	**1680**	**1680**	**ND1**	-	0.38	0.17	0.91	0.80	-	0.33	0.48	tr	0.19	-	-
***Oxygenated sesquiterpenoid**	**28.82**	**1692**	**ND1**	**ND1**	-	0.50	0.21	-	0.15	-	0.17	0.18	0.39	0.23	-	-
**Methyl tetradecanoate**	**28.94**	**1704**	**ND1**	**1708**	-	0.38	0.53	0.23	0.23	0.35	0.89	0.34	0.26	0.47	-	0.13
**Benzyl benzoate**	**29.10**	**1726**	**1730**	**1732**	19.58	20.49	13.75	7.40	11.79	1.28	17.64	19.32	4tr	19.19	-	6.26
**γ-Costol**	**29.20**	**1737**	**1730**	***1752**	1.00	0.61	0.08	0.06	0.07	tr	-	0.21	-	0.58	-	-
***15-Hydroxy-α-muurolene**	**29.22**	**1740**	**ND1**	**1757**	0.42	-	-	-	-	-	-	-	-	-	-	-
**β-Costol**	**29.32**	**1753**	**1754**	**1769**	0.38	-	-	-	-	-	-	-	-	-	-	-
***7-(2-Hydroxypropan-2-yl)-1,4a-dimethyldecahydronaphthalen-1-ol**	**29.37**	**1758**	**ND1**	**ND1**	-	-	-	-	-	-	-	-	-	-	-	0.12

Suplement 3H.

Component	RT	KI_{EXP}	KI_{MF}	KI_{NIST}	PR-LS1	PR-LS2	PR-LS3	PR-LS4	PR-LS5	PR-LS6	PR-S1	PR-S2	PR-MR	PR-NSW	PR-GR	PR-CN
**α-Costol**	**29.48**	**1772**	**1761**	**1757**	4.58	2.86	0.58	0.53	0.54	-	0.14	0.97	0.14	3.27	-	-
**Selina-4α,11-diol**	**29.60**	**1787**	**ND1**	**1780**	-	-	-	-	-	-	-	-	-	-	-	tr
***Oxygenated sesquiterpenoid**	**29.69**	**1797**	**ND1**	**ND1**	0.15	0.49	0.10	tr	0.07	0.17	-	0.39	-	0.44	-	-
**Phenylethyl benzoate**	**29.83**	**1813**	**1815**	**1828**	0.08	0.05	0.65	0.73	0.79	0.09	2.08	0.69	0.08	0.06	-	0.20
**Benzyl salicylate**	**29.98**	**1831**	**1847**	**1836**	3.74	1.74	3.29	1.38	3.95	0.22	5.01	2.42	5.43	2.67	-	0.67
***Methyl 7-hexadecenoate**	**30.19**	**1869**	**ND1**	**1888**	-	-	-	-	-	-	-	-	-	-	-	-
***Aliphatic alcohol**	**30.41**	**1900**	**ND1**	**ND1**	-	0.25	0.46	0.20	0.46	-	1.61	0.62	-	0.42	-	0.27
**Methyl 4,7,10,13-hexadecatetraenote**	**30.46**	**1906**	**ND1**	**ND1**	0.69	-	-	-	-	-	-	-	-	-	0.12	0.11
***Aliphatic component**	**31.17**	**2004**	**ND1**	**ND1**	-	-	-	-	-	0.13	-	-	-	-	-	-
****cis*-Benzyl cinnamate**	**31.32**	**2029**	**ND1**	**1959**	-	-	-	-	-	-	-	-	-	-	-	0.85
***trans*-Benzyl cinnamate**	**31.51**	**2059**	**2023**	**ND1**	3.97	1.33	3.69	0.41	1.21	0.18	3.45	4.93	4.82	3.56	-	0.41
***Aliphatic ester**	**31.63**	**2077**	**ND1**	**ND1**	-	-	-	-	-	-	-	-	-	-	-	0.20
**n-Heneicosane**	**31.74**	**2094**	**2100**	**2100**	0.41	0.43	0.53	0.17	0.37	-	1.41	0.50	0.20	0.61	-	0.37
***Benzyl dodecanoate**	**31.95**	**2128**	**ND1**	**2097**	0.39	-	-	-	-	0.45	-	-	-	-	-	-
***Aliphatic component**	**32.33**	**2189**	**ND1**	**ND1**	-	0.09	0.22	0.05	0.08	-	0.38	0.11	0.07	0.15	-	0.30
***Aliphatic component**	**32.77**	**2265**	**ND1**	**ND1**	0.18	0.11	0.32	0.05	0.05	0.10	-	0.12	0.07	0.13	-	-
***Aliphatic component**	**32.89**	**2286**	**ND1**	**ND1**	-	-	-	-	-	0.10	-	-	-	-	-	0.05
**Tricosane**	**32.91**	**2290**	**2301**	**2300**	0.12	0.98	2.03	0.33	0.62	1.23	2.44	1.39	0.60	1.26	-	0.70
***Aliphatic component**	**33.47**	**ND**	**ND1**	**ND1**	-	-	-	-	-	-	-	-	-	-	-	0.24
***Aliphatic component**	**33.85**	**ND**	**ND1**	**ND1**	0.10	0.05	0.17	tr	0.06	0.08	0.28	0.08	0.09	0.12	-	tr
***Aliphatic component**	**34.00**	**ND**	**ND1**	**ND1**	-	0.57	0.88	0.18	0.28	-	0.91	0.77	0.29	0.62	-	0.47
***Aliphatic component**	**34.07**	**ND**	**ND1**	**ND1**	-	-	-	-	-	0.66	-	-	-	-	-	0.69
***Aliphatic component**	**34.50**	**ND**	**ND1**	**ND1**	-	-	-	-	-	-	-	-	-	-	-	0.06

Legend

*– amount of single component calculated as percent (%) of whole GC-MS chromatogram area
** – possible xenobiotic
tr – trace concentration of component <0,05%
"–" - substance under detection condition

KI_{EXP} – Experimental retention index
KI_{MF} – Retention index according to Terpenoid Library **[1]**
KI_{NIST} – Retention index according to NIST Chemistry WebBook **[2]**
ND – Retention Indices was not determined
ND1 – No data for DB-1 capillary column type

Literature

1. König, W.A.; Joulain, D.; Grasse, R.S.; Hochmuth, D.H. Terpenoids and related constituents of essential oils, Hochmuth Scientific Consulting. Aviable on: http://massfinder.com/wiki/Terpenoids_Library. (accessed on: 15th May 2017)
2. National Institute of Standard and Technology. NIST Chemistry WebBook. Old York Road, Ringoes, USA, National Institute of Standards and Technology. Aviable on: http://webbook.nist.gov/chemistry/ (accessed on: 15th May 2017)

Suplement 4A. UPLC-DAD-MS identification of 70% ethanol in water extracts composition

NB.	Component	RT	UV λ max (nm)	$[M-H]^-$	Fragments	CE (kV)	MI	Rf
1	Caffeic acid	0.83	**324**, 298sh, 242	179	135	20	A, B, C	[1, 2, 3, 4, 5, 6, 7, 8]
2	*p*-Coumaric acid	1.23	**310**, 300sh, 229	163	119	20	A, B, C	[1, 2, 3, 4, 5, 6, 7, 8]
3	Vanilline	1.34	**310**,280, 231	151	135, 121	0	A, B, C	[6, 8]
4	*Salicortin	1.34	320, 278	423	317, 161, 155, 137, 123, 111, 109, 83	20	B, C	[9]
5	Ferulic acid	1.44	**324**, 298sh, 236	193	149, 134	20-30	A, B, C	[1, 2, 3, 4, 6, 7, 8]
6	Isoferulic acid	1.6	**323**, 295sh, 221	193	149, 134	20-30	A, B, C	[1, 2, 3, 6, 7, 8]
7	Benzoic acid	2.33	281sh, 274sh, **236**	123	103, 77	20	A, B, C	[6, 8]
8	Acetyl-*p*-coumaroyl glycerol	2.94	**312,** 300sh	279	219, 163, 145, 119	20	C	[10, 11]
9	3,4-Dimethyl caffeic acid (DMCA)	3.35	**322**, 294sh, 236	207	163, 133	20	B, C	[3, 7]
10	*Chrysin derivate	3.67	315sh, **265**	431	268, 239, 195	20-30	B, C	ND
11	Quercetin	4.91	368, 293sh, 270sh, **256**	301	271, 179, 151, 121, 107	40	A, B, C	[3, 6, 7, 12]
12	Cinnamic acid	5.30	**277**	147	103	20	A, B, C	[3, 6, 7, 13]
13	Pinobanksin 5-O-methyl ether	5.41	322sh, **288**, 228	285	267, 252, 239, 224, 208, 195, 180, 165, 152, 136	20-30	B, C	[2, 3, 5, 7]
14	Quercetin 3-O-methyl ether	6.08	355, 293sh, 268sh, **255**	315	300, 271, 243	30-50	B, C	[3]
15	**1-Caffeoyl-3-*p*-coumaroyl glycerol	6.75	**315**, 298sh, 235	399	253, 235, 219, 179, 163, 135, 119	20	B, C	[1, 7]
16	Pinobanksin	7.01	332sh, **292**, 229	271	253, 225, 209, 185, 151, 107	20	A, B, C	[1, 2, 3, 7]
17	Apigenin	7.26	**338**, 290sh, 268, 226	269	227, 181, 151, 149, 117, 107	20-30	A, B, C	[3, 7, 12]
18	Caffeoyl-feruloylglycerol isomer 1	7.33	**326**, 298sh, 240	429	253, 235, 179, 135, 134	20	C	[10]

Suplement 4B.

NB.	Component	RT	UV λ max (nm)	[M-H]-	Fragments	CE (kV)	MI	Rf
19	Caffeoyl-feruloylglycerol isomer 2	7.55	**326**, 298sh, 240	429	267, 253, 249, 235, 193, 179, 149, 135, 134	20	C	[10]
20	Chrysin 5-O-Methyl ether	7.59	314sh, **264**, 247sh	267	252, 242, 180	20	B, C	[5, 13]
21	Kaemferol	7.72	366, 322sh, 295sh, 266,	285	-	20	A, B, C	[1, 6, 7, 12]
22	Isoramnetin	8.31	371, 298sh, 268sh, **255**,	315	300, 151	20-30	B, C	[1, 2, 6, 7, 12, 13]
23	Luteolin 5-O-methyl ether	9.02	350, 298sh, **266**,	299	284, 255, 227, 211	30-50	B, C	[3]
24	**1,3-Di-*p*-coumaroylglycerol	9.25	**310**, 300sh, 233	383	237, 219, 163, 145, 119	20	C	[1]
25	*Caffeic acid propyl or isopropyl ester	9.35	**326**, 298sh, 245	221	179, 161, 135	20	C	[8]
26	Quercetin-O-5-O7-dimethyl ether	9.64	356, 296sh, 269sh, **255**	329	314, 299, 285, 271, 257, 243, 227	30-50	B, C	[3, 7]
27	*p*-Coumaroyl-feruloylglycerol isomer 1	9.73	**316**, 298sh, 233	413	235, 193, 163	20-30	B, C	[7]
28	*p*-Coumaroyl-feruloylglycerol isomer 2	9.84	**316**, 298sh, 233	413	235, 193, 163	20-30	B, C	[7]
29	***Di*- 1,3-feruloylglycerol	10.17	**326**, 299sh, 245	443	249, 193	20	C	[10, 11]
30	2-Acetyl-1,3-*di*-caffeoylglycerol	10.38	**328**, 298sh, 244	457	397, 295, 235, 173, 179, 163, 161, 135	20	B, C	[4, 7, 10]
31	*β*-styryloacrylic acid	10.60	**311**, 240sh	173	-	0	B, C	[3, 15]
32	Galangin-5-O-Methylether	11.05	352, 300sh, **261**, 240sh	283	268, 239, 211	20-30	B, C	[2, 3]
33	*Caffeic acid butenic or isobutenic ester	11.17	**326**, 298sh, 245	233	179, 161, 135	20	C	[16]

Suplement 4C.

NB.	Component	RT	UV λ max (nm)	[M-H]-	Fragments	CE (kV)	MI	Rf
34	Rhamnetin	11.68	354, 268sh, **255**	315	300, 165, 151, 193, 121, 109	20-30	B,C	[7, 12, 17]
35	*p-Coumaric acid propyl or isopropyl ester	13.09	311, 300sh	205	163, 145, 119	20	C	[8]
36	2-Acetyl-1-caffeoyl-3-*p*-coumaroylglycerol	13.16	316, 299sh 235	441	381, 295, 235, 163, 135, 119	20-30	B, C	[1, 4, 7, 10]
37	Quercetin-O,O-dimethyl ether	13.24	356, 268sh, **256**	329	314, 299, 271, 243, 227	20-30	B, C	[7, 14]
38	*Caffeic acid butyl or isobutyl ester isomer 1	13.37	**326**, 298sh, 245	235	179, 161, 135	20	C	[1, 16]
39	2-Acetyl-3-caffeoyl-1-feruloylglycerol	13.87	**328**, 300sh, 244	471	411, 295, 235, 193, 179, 149, 135	20-30	B,C	[4, 10]
40	* Caffeic acid butyl or isobutyl ester isomer 2	14.1	**326**, 298sh, 245	235	179, 161, 135	20	C	[1, 16]
41	Chrysin	14.31	314sh, **268,** 246sh	253	209, 181, 167, 165, 151, 107, 145, 143, 119	20-30	A, B, C	[1, 3, 4, 5, 6, 7]
42	Caffeic acid prenyl or isoprenyl ester isomer 1	14.41	**326**, 298sh, 246	247	179, 161, 135	20	B, C	[2, 3, 5, 6, 7]
43	Sakuranetin (Naringenin-7-O-methyl ester)	14.96	328sh, **291**, 270sh,	285	270, 243, 164, 151, 136, 107	20-30	B, C	[6, 7, 13]
44	Pinocembrin	15.2	330sh, **290**, 235	255	213, 187, 171, 164, 151, 145, 136,	20	B, C	[3, 4, 5, 6, 7]
45	Caffeic acid prenyl or isoprenyl ester isomer 2	15.36	**326**, 298sh, 246	247	179, 161, 135	20	B, C	[3, 4, 6, 7]
46	Caffeic acid benzyl ester	15.66	**328**, 298sh, 244	269	178, 161, 134	20	B, C	[2, 3, 7]
47	*Pinocembrin chalcone	15.74	**345**	255	213, 151, 101	20-30	C	[1, 18]
48	Galangin	16.21	360, 290sh, **266**, 240sh	269	227, 197, 183, 151	20-30	B, C	[1, 2, 3, 5, 7, 14]
49	Pinobansin-3-O-acetate	17.25	332sh, **294**, 238	313	271, 253, 209, 181, 165, 143, 151, 107	30-50	B, C	[1, 2, 3, 7]
50	**2-Acetyl-1,3-di-*p*-coumaroylglycerol	17.55	360sh, **312**, 232	425	365, 321, 215, 163, 119	30-50	B, C	[1, 7, 10]
51	Metoxychrysin	18.23	340sh, 310sh, **266,** 245sh	283	268, 239, 211, 195	20	B, C	[2, 3, 15]
52	**2-Acetyl-3-*p*-coumaroyl-1-feruloylglycerol	18.34	**318**, 299sh 235	455	395, 351, 193, 163, 149, 119	20	B, C	[1, 4]

Suplement 4D.

NB.	Component	RT	UV λ max (nm)	[M-H]-	Fragments	CE (kV)	MI	Rf
53	Caffeic acid phenethyl ester (CAPE)	18.58	321, 300sh, 264	283	179, 161, 119	20	B, C	[1, 2, 3, 5, 7]
54	**3-Acetyl-1,2-di-*p*-coumaroylglycerol	18.62	**312**, 300sh, 238	425	365, 163	20	C	[1]
55	**2-Acetyl-1,3-di-feruloilo glycerol	18.99	**328**, 298sh, 243	485	425, 249, 230, 193, 175, 149, 134	20	B, C	[1, 4, 11]
56	Caffeic acid pentyl or isopentyl ester	19.46	**326**, 298sh, 246	249	179, 161, 119	20	B, C	[16, 19]
57	* *p*-Coumaric acid butyl or isobutyle ester	19.81	**311**, 299sh, 245sh	219	163, 145, 119	20	C	[8]
58	*p*-Coumaric acid prenyl or isoprenyl ester isomer 1	20.17	**311**, 299sh, 245sh	231	163, 145, 119	20	B, C	[2, 3, 5]
59	*p*-Coumaric acid prenyl or isoprenyl ester isomer 2	20.9	**311**, 299sh, 245sh	231	163, 145, 119	20	B, C	[3, 5]
60	*p*-Coumaric acid benzyl ester	21.08	**312**, 298sh, 244sh	253	162, 145, 118	20	B, C	[1, 2, 3]
61	*p*-Coumaric acid prenyl or isoprenyl ester isomer 3	21.83	**311**, 299sh, 238sh	231	163, 145, 119	20	B, C	[3]
62	*Ferulic acid benzyl ester	21.83	**326**, 298, 242	283	-	20	C	[1, 8, 20]
63	Caffeic acid cinnamyl ester	22.29	**326**, 300sh, 243	295	178, 163, 134, 92	20	B, C	[3, 7, 15]
64	Pinobansin-3-O-propionate	22.42	329sh, **294**, 234	327	271, 253, 225, 209, 181, 165, 143	20	B, C	[2, 3, 7]
65	*p*-Coumaric acid phenethyl ester	22.88	**312**, 300sh, 242sh	267	163, 145, 119	20	B, C	[5]
66	Pinostrobin chalcone (2,6-Dihydroxy-4'metoxychalkon)	22.92	**345**, 309sh, 267	269	254, 226, 198, 171, 165, 136, 122	20	B, C	[1, 18]
67	* *p*-Coumaric acid pentyl or isopentyl ester	23.95	**312**, 300sh, 242sh	233	163, 145, 119	20	C	[8]
68	Tectochrysin (Chrysin-7-O-methyl ether)	24.69	310sh, **268**	267	-	-	B, C	[2, 9]
69	Pinostrobin (Pinocembrin-7-O-methyl ether)	24.97	328sh, **290**, 248sh	-	-	-	B, C	[7]
70	*p*-Coumaric acid cinnamyl ester	25.49	**312**, 300sh, 250	279	119	20	B, C	[5, 15]
71	*3-O-Methylgalangin	25.75	353, **268**	-	-	-	-	[21]

Suplement 4E.

NB.	Component	RT	UV λ max (nm)	[M-H]-	Fragments	CE (kV)	MI	Rf
72	Pinobanksin-3-O-butanoate or isobutanoate	25.94	320sh, **293**, 240sh	341	271, 253, 209, 181, 165, 151, 107	30-50	B, C	[2, 3]
73	Pinobanksin-3-O-pentenoate or isopentenoate isomer 1	26.36	320sh, **294**, 242	353	271, 253, 209, 181, 165, 151, 107	30-50	B, C	[7, 14, 22]
74	Pinobanksin-3-O-pentenoate or isopentenoate isomer 2	26.48	320sh, **295**, 250	353	271, 253, 209, 181, 165, 151, 107	20-30	B, C	[7, 14, 22]
75	Pinobanksin-3-O-pentanoate or isopentanoate	29.05	332sh, **293**, 242	355	253, 209, 181, 165, 143, 107, 101	30-50	B, C	[2, 3, 7]
76	Pinobanksin-3-O-hexanoate or hexanoate	31.70	**282**	369	271, 253, 209, 151, 143	20	B, C	[2, 3, 15]
77	Metoxycinnamic acid cinnanyl ester	31.80	**280**	293	-	0	B, C	[3, 15]

Legenda

MI –method of identification
A – comparison with the standard
B – comparison with HPLC/UPLC-DAD-MS literature data
C – prediction of mass fragmentation
***** – component identified tentatively
– no ionisation under detection condition

RT – experimental retention time [min.]
EK_{exp} (kV) – mass fragmentation optimal collision energy under experimental conditions (kV)
$UV_{\lambda\ max}$ (nm) – UV maximum absortion (nm); the highest maximum is in **bold** and others UV maximums in normal font
sh – shoulder UV absortion maximum
Rf – references
ND – no literature data
****** – glycerol substitution condition predicted according to comparison with GC-MS data

Suplement 4: Literature.

1. Isidorov, V.A.; Szczepaniak, L.; Bakier, S. Rapid gc/ms determination of botanical precursors of Eurasian propoliss. *Food Chem.* **2014**, 142, 101–106.
2. Shi, H.; Yang, H.; Zhang, X.; Yu, L.L. Identification and quantification of phytochemical composition and anti-inflammatory and radical scavenging properties of methanolic extracts of chinese propolis. *J. Agric. Food Chem.* **2012**, 60, 12403–1210.
3. Pellati, F.; Orlandinia, G.; Pinetti, D.; Benvenutia, S. HPLC-DAD and HPLC-ESI-MS/MS methods for metabolite profiling of propolis extracts. *J. Pharm. Biomed. Anal.* **2011**, 55, 934–948.
4. Shi, H.; Yang, H.; Zhang, X.; Sheng, Y.; Huang, H.; Yu, L. Isolation and characterization of five glycerol esters from Wuhan propolis and their potential anti-inflammatory properties. J. Agric. Food Chem. **2012**, 60, 10041–10047
5. Trudić, B.; Anđelković, B.; Orlović, S.; Tešević, V.; Pilipović, A.; Cvetković, **M.;** Stanković, J. HPLC/MS-TOF analysis of surface resins from three poplar clones grown in Serbia. South-east Eur. for. 7. 2016, doi: 10.15177/seefor.16-12
6. Wishart Research Group. HMDB: the Human Metabolome Database. University of Alberta, Edmonton, Canada, Wishart Lab. Aviable online: www.hmdb.ca. (accesed on 30th August 2017)
7. Ristivojević, P.; Trifković, J.; Gašić, U.; Andrić, F.; Nedić, N.; Tešić, Ž.; Milojković-Opsenica, D. Ultrahigh-performance liquid chromatography and mass spectrometry (UHPLC-LTQ/Orbitrap/MS/MS) study of phenolic profile of Serbian poplar type propolis. *Phytochem. Anal.* **2015**, 26, 127–136.
8. Popova, M.; Giannopoulou, E.; Skalicka-Woźniak, K.; Graikou, K.; Widelski, J.; Bankova, V.; Kalofonos, H.; Sivolapenko, G.; Gaweł-Bęben, K.; Antosiewicz, B.; Chinou, I. Characterization and biological evaluation of propolis from Poland. Molecules. **2017**, 22, doi: 10.3390/molecules22071159.
9. Abreu, I.N.; Ahnlund, M.; Moritz, T.; Albrectsen, B.R. UHPLC-ESI/TOFMS Determination of Salicylate-like Phenolic Gycosides in Populus tremula Leaves. *J. Chem. Ecol.* **2011**, 37, 857–870.
10. Bertrams, J.; Müller, B.M.; Kunz, N.; Kammerer, D.R.; Stintzing, F.C. Phenolic compounds as marker compounds for botanical origin determination of German propolis samples based on TLC and TLC-MS. *J. Appl. Bot. Food Qual.* **2013**, 86, 143–153.
11. Isidorov, V.A.; Brzozowska, M.; Czyżewska, U.; Glinka, L. Gas chromatographic investigation of phenylpropenoid glycerides from aspen (*Populus tremula* L.) buds. *J. Chromatogr. A.* **2008**, 1198-1199, 196–201.
12. Ristivojević, P.; Trifković, J.; Andrić, F.; Milojković-Opsenica, D. Poplar-type propolis: Chemical composition, botanical origin and biological activity. *Nat. Prod. Commun.* **2015**, 11, 1869–1876.
13. Justesen, U. Negative atmospheric pressure chemical ionisation low-energy collision activation mass spectrometry for the characterisation of flavonoids in extracts of fresh herbs. *J. Chromatogr. A.* **2000**, 902, 369–379.
14. Metlin Scripps Center for Metabolomics and Mass Spectrometry (Metlin). La Jolla, USA The Scripps Research Institute. Aviable online: https://metlin.scripps.edu/index.php. (accesed on 28th August 2017)
15. Gardana, C.; Scaglianti, M.; Pietta, P.; Simonetti, P. Analysis of the polyphenolic fraction of propolis from different sources by liquid chromatography–tandem mass spectrometry. *J. Pharm. Biomed. Anal.* **2007**, 45, 390–399.
16. Greenaway, W.; Wollenweber, E.; Scaysbrook, T.; Whatley, F.R. Esters of caffeic acid with aliphatic alcohols in bud exudate of *Populus nigra*. *Z. Naturforsch. C.* **1988**, 43, 795–798.
17. Justen, U. Collision-induced fragmentation of deprotonated methoxylated flavonoids, obtained by electrospray ionization mass spectrometry. *J. Mass Spectrom.* **2001**, 36, 169–178.
18. Greenaway, W.; May, J.; Scaysbrook, T.; Whatley, F.R. Compositions of bud and leaf exudates of some *Populus* species compared. *Z. Naturforsch. C.* **1992**, 47, 329-334.
19. Medana, C.; Carbone, F.; Aigotti, R.; Appendino, G.; Baiocchi, C. Selective analysis of phenolic compounds in propolis by HPLC-MS/MS. *Phytochem. Anal.* **2008,** 19, 32–39.
20. Greenaway, W.; Whatley, F.R. Bud exudate composition of *Populus tremuloides*. *Can. J. Bot.* **1991**, 69, 2291–2295.
21. Greenaway, W.; Scaysbrook, T.; Whatley, F.R. The Composition and Plant Origins of Propolis: A Report of Work at Oxford. **1988,** 71, 107-118.
22. Kečkeš, S.; Gašić, U.; Veličković, T.Ć.; Milojković-Opsenica, D.; Natić, M.; Tešić, Ž. The determination of phenolic profiles of Serbian unifloral honeys using ultra-high-performance liquid chromatography/high resolution accurate mass spectrometry. *Food Chem.* **2013,** 138, 32–40.

Suplement 5A. Composition of *Populus* spp. buds 70% ethanol in water extracts*

Component	RT	Aspens (*Populus tremula* L.)						Black poplars (*Populus nigra* L.)						
		PT1	PT2	PT3	PT4	PT5	PT6	PN1	PN2	PN3	PN4	PN5	PN6	PN7
**Free phenolic acids**														
Caffeic acid	**0.83**	1.12	0.93	0,75	0,90	0,51	1,40	2,18	3,84	3,86	1,16	3,19	1,15	0,96
p-Coumaric acid	**1.23**	3.49	1.67	4,37	2,51	1,15	1,65	3,24	2,59	0,14	14,38	1,77	12,45	12,90
Ferulic acid	**1.44**	3.23	1.20	1,94	2,54	1,45	1,24	0,81	0,90	0,10	0,12	0,49	0,48	-
Isoferulic acid	**1.60**	-	-	-	-	-	-	0,54	0,62	0,41	0,23	1,43	0,18	-
3,4-Dimethyl caffeic acid (DMCA)	**3.35**	-	-	-	-	-	-	-	-	-	-	3,81	0,07	-
Cinnamic acid	**5.30**	-	-	-	-	-	-	0,08	-	-	-	-	0,16	0,05
β-styryloacrylic acid	**10.60**	-	-	-	-	-	-	1,43	0,15	-	-	-	-	-
**Phenolic acids glycerides**														
Caffeoyl-feruloylglycerol isomer 1	**7.33**	0.53	0.11	-	0,66	0,67	0,26	-	-	-	-	-	-	-
Caffeoyl-feruloylglycerol isomer 2	**7.55**	0.89	0.93	0,38	0,72	0,99	1,41	-	-	-	-	-	-	-
**1,3-Di-*p*-coumaroylglycerol	**9.25**	6.68	12.98	3,79	1,47	1,37	2,88	-	-	-	-	-	-	-
p-Coumaroyl- feruloylglycerol isomer 1	**9.73**	1.39	0.98	0,37	0,72	0,54	0,52	-	-	-	-	-	-	-
p-Coumaroyl- feruloylglycerol isomer 2	**9.84**	0.27	-	-	0,08	0,14	-	-	-	-	-	-	-	-
**1,3-Di-*p*-coumaroylglycerol	**10.17**	0.23	-	-	0,24	0,15	-	-	-	-	-	-	-	-
2-Acetyl-1,3-*di*-caffeoylglycerol	**10.38**	5.50	0.47	0,31	10,46	17,30	17,37	-	-	-	-	-	-	-
2-Acetyl-1-caffeoyl-3-*p*-coumaroylglycerol	**13.16**	10.97	4.19	3,88	13,83	17,74	16,93	-	-	-	-	-	-	-
2-Acetyl-3-caffeoyl-1-feruloylglycerol	**13.87**	6.48	0.92	0,25	12,28	13,50	5,52	-	-	-	-	-	-	-

Suplement 5B.

Component	RT	Aspens (*Populus tremula* L.)						Black poplars (*Populus nigra* L.)						
		PT1	PT2	PT3	PT4	PT5	PT6	PN1	PN2	PN3	PN4	PN5	PN6	PN7
**Phenolic acid glycerides**														
**2-Acetyl-1,3-di-*p*-coumaroylglycerol	**17.52**	22.21	37.91	37.74	13.74	11.82	9.63	-	-	-	-	-	-	-
**2-Acetyl-3-*p*-coumaroyl-1-feruloylglycerol	**18.34**	10.19	7.19	6.49	14.07	10.34	2.02	-	-	-	-	-	-	-
**3-Acetyl-1,2-di-*p*-coumaroylglycerol	**18.62**	-	0.42	0.60	-	-	0.21	-	-	-	-	-	-	-
**2-Acetyl-1,3-di-feruloilo glycerol	**18.99**	5.56	1.41	0.65	8.46	6.41	1.83	-	-	-	-	-	-	-
**Phenolic acids monoesters**														
*Caffeic acid propyl or isopropyl ester	**9.35**	-	-	-	-	-	-	-	-	0.68	-	-	-	-
*Caffeic acid butenic or isobutenic ester	**11.17**	-	-	-	-	-	-	-	-	0.16	0.15	0.32	-	-
**p*-Coumaric acid propyl or isopropyl ester	**13.09**	-	-	-	-	-	-	-	-	-	0.38	-	0.28	0.30
*Caffeic acid butyl or isobutyl ester isomer 1	**13.37**	-	-	-	-	-	-	-	-	3.91	0.29	-	0.31	-
*Caffeic acid butyl or isobutyl ester isomer 2	**14.10**	-	-	-	-	-	-	-	-	1.39	-	-	-	-
Caffeic acid prenyl or isoprenyl ester isomer 1	**14.41**	-	-	-	-	-	-	0.66	0.63	1.22	-	1.35	0.12	0.12
Caffeic acid prenyl or isoprenyl ester isomer 2	**15.36**	-	-	-	-	-	-	1.05	1.53	1.80	-	2.33	0.51	1.78
Caffeic acid benzyl ester	**15.66**	0.38	-	0.40	0.34	0.23	5.89	-	-	-	-	-	-	-
Caffeic acid phenethyl ester (CAPE)	**18.58**	-	-	-	-	-	-	1.00	1.02	2.85	0.34	1.76	0.50	0.38
Caffeic acid pentyl or isopentyl ester	**19.46**	-	-	-	-	-	-	-	-	9.77	2.38	1.00	2.87	0.43
* *p*-Coumaric acid butyl or isobutyl ester	**19.81**	-	-	-	-	-	-	-	-	-	-	-	025	tr
p-Coumaric acid prenyl or isoprenyl ester isomer 1	**20.17**	-	-	-	-	-	-	0.70	0.28	-	-	-	0.27	0.43

Suplement 5C.

Component	RT	Aspens (*Populus tremula* L.)						Black poplars (*Populus nigra* L.)						
		PT1	PT2	PT3	PT4	PT5	PT6	PN1	PN2	PN3	PN4	PN5	PN6	PN7
**Phenolic acids monoesters**														
p-Coumaric acid prenyl or isoprenyl ester isomer 2	**20.80**	-	-	-	-	-	-	-	0.89	-	-	-	-	-
p-Coumaric acid benzyl ester	**21.08**	1.93	2.02	16.39	1.08	0.35	6.78	2.97	0.45	-	1.16	-	1.53	2.04
p-Coumaric acid prenyl or isoprenyl ester isomer 3	**21.83**	-	-	-	-	-	-	-	-	-	4.03	-	5.00	4.97
*Ferulic acid benzyl ester	**21.83**	1.94	0.95	3.23	1.37	0.55	3.38	1.16	0.79	-	-	-	1.42	2.05
Caffeic acid cinnamyl ester	**22.29**	-	-	-	-	-	-	-	0.20	0.26	0.68	0.17	1.50	1.20
p-Coumaric acid phenethyl ester	**22.88**	-	-	-	0.07	0.07	0.41	-	0.30	-	2.19	-	1.31	2.01
* *p*-Coumaric acid pentyl or isopentyl ester	**23.95**	-	-	-	-	-	-	-	-	-	2.49	-	0.44	0.80
p-Coumaric acid cinnamyl ester	**25.49**	-	-	-	-	-	-	-	-	-	5.61	0.63	8.25	9.80
Metoxycinnamic acid cinnanyl ester	**31.18**	-	-	-	-	-	-	2.72	3.43	0.27	-	-	-	-
**Flavones**														
*Chrysin derivate	**3.67**	-	-	-	-	-	-	0.49	0.50	0.59	-	-	-	-
Apigenin	**7.26**	0.52	0.06	0.34	0.23	0.19	0.23	5.34	4.72	0.32	1.09	0.92	0.47	0.50
Chrysin 5-O-methyl ether	**7.59**	-	-	-	-	-	-	-	0.09	-	-	-	-	-
Luteolin 5-O-methyl ether	**9.02**	-	-	-	-	-	-	2.16	1.89	-	-	0.20	-	-
Chrysin	**14.31**	-	-	-	-	-	-	13.10	13.80	4.95	1.52	8.11	2.50	1.62
Metoxychrysin	**18.23**	-	-	-	-	-	-	2.98	3.26	0.40	-	0.78	-	-
Tectochrysin	**24.69**	-	-	-	-	-	-	5.46	1.15	-	0.77	1.12	-	-
**Flavanones**														
Sakuranetin	**14.96**	5.01	13.10	9.35	6.47	6.16	5.85	-	0.25	-	0.73	0.27	0.17	0.37
Pinocembrin	**15.20**	-	-	-	-	-	-	9.01	5.03	5.07	7.81	9.79	7.74	7.61

Suplement 5D.

Components	RT	Aspens (*Populus tremula* L.)						Black poplars (*Populus nigra* L.)						
		PT1	PT2	PT3	PT4	PT5	PT6	PN1	PN2	PN3	PN4	PN5	PN6	PN7
**Flavanones**														
Pinostrobin	**24.97**	-	-	-	-	-	-	0.57	0.39	3.82	2.69	5.91	5.25	7.31
**Flavonols**														
Quercetin	**4.91**	-	-	-	-	-	-	0.50	0.65	0.11	0.20	0.13	-	0.07
Quercetin-3-O-methyl ether	**6.08**	-	-	-	-	-	-	0.94	1.00	0.27	-	0.07	-	-
Kaempferol	**7.72**	0.65	0.40	0.27	0.14	0.10	-	1.61	1.72	0.15	1.82	0.05	0.48	0.69
Isorhamnetin	**8.31**	0.43	0.21	0.59	0.22	0.73	0.37	0.18	0.18	0.83	-	0.45	-	-
Quercetin-5,7-O-dimethyl ether	**9.64**	-	-	-	-	-	-	0.63	0.43	-	-	-	-	-
Galangin-5-O-methyl ether	**11.05**	-	-	-	-	-	-	0.64	0.48	-	-	-	-	-
Rhamnetin	**11.68**	-	-	-	-	-	-	0.87	0.78	-	0.20	0.24	-	-
Quercetin-O,O-dimethyl ether	**13.24**	-	-	-	-	-	-	1.05	0.89	-	-	-	-	-
Galangin	**16.21**	-	-	-	-	-	-	15.34	15.27	11.00	4.61	7.22	2.72	3.09
Kaempferid	**16.99**	3.03	3.78	2.41	1.13	1.24	1.38	0.31	0.29	0.23	2.79	-	1.16	1.50
*Galangin-3-O-methyl ether	**25.75**	-	-	-	-	-	-	1.56	0.76	0.89	2.71	0.53	3.43	1.65
**Flavanonols**														
Pinobanksin-5-O-methyl ether	**5.41**	-	-	-	-	-	-	1.34	1.44	-	-	-	0.11	-
Pinobanksin	**7.01**	4.09	3.59	2.27	2.85	2.44	1.36	4.32	4.83	1.52	1.06	1.16	0.70	0.84
Pinobansin-3-O-acetate	**17.25**	-	-	-	-	-	-	7.24	7.07	11.32	1.53	5.89	3.28	2.91
Pinobanksin-3-O-propionate	**22.42**	-	-	-	-	-	-	1.14	1.25	-	-	-	-	-
Pinobanksin-3-O-butanoate or isobutanoate	**25.94**	-	-	-	-	-	-	0.78	1.77	-	-	-	-	-

Suplement 5E.

Components	RT	Aspens (*Populus tremula* L.)						Black poplars (*Populus nigra* L.)						
		PT1	PT2	PT3	PT4	PT5	PT6	PN1	PN2	PN3	PN4	PN5	PN6	PN7
**Flavanonols**														
Pinobanksin-3-O-pentenoate or isopentenoate isomer 1	**26.36**	-	-	-	-	-	-	0.49	0.53	-	-	0.34	-	-
Pinobanksin-3-O-pentenoate or isopentenoate isomer 2	**26.48**	-	-	-	-	-	-	-	0.31	-	-	-	-	-
Pinobanksin-3-O-pentanoate or isopentanoate	**29.05**	-	-	-	-	-	-	0.97	1.32	-	-	-	-	-
Pinobanksin-3-O-hexanoate or hexanoate	**31.70**	-	-	-	-	-	-	tr	tr	tr	tr	tr	-	-
**Chalkons**														
Pinocembrin chalkone	**15.74**	-	-	-	-	-	-	-	4.18	17.99	1.82	21.79	2.23	-
Pinostrobin chalcone	**22.92**	-	-	-	-	-	-	-	0.92	10.59	5.61	10.80	5.60	4.38
**Others**														
Salicortin	**1.34**	-	-	-	-	-	-	0.10	0.10	0.22	0.20	0.66	0.44	-
Vanilline	**1.34**	0.31	-	0.38	0.06	-	0.46	-	-	-	-	-	-	-
**Unidentified peaks**														
Unidentified	**1.17**	0.38	0.40	0.55	0.27	0.23	0.61	0.12	-	-	0.07	0.19	-	-
Unidentified	**2.33**	-	-	-	-	-	-	0.28	0.27	0.18	0.49	0.36	1.40	0.32
Unidentified	**2.46**	1.20	1.05	0.75	0.26	0.33	2.31	-	-	-	-	-	-	-
Unidentified	**4.47**	-	0.86	-	1.06	1.05	1.09	-	-	-	-	-	-	-
Unidentified	**4.80**	-	0.45	-	-	1.06	1.09	-	-	-	-	-	-	-
Unidentified	**7.45**	-	-	-	-	-	-	-	-	-	0.54	-	0.21	0.23

Suplement 5F.

Components	RT	Aspens (*Populus tremula* L.)						Black poplars (*Populus nigra* L.)						
		**PT1**	**PT2**	**PT3**	**PT4**	**PT5**	**PT6**	**PN1**	**PN2**	**PN3**	**PN4**	**PN5**	**PN6**	**PN7**
**Unidentified peaks**														
Unidentified	**8.07**	-	-	-	-	-	-	-	-	0.23	-	0.33	-	-
Unidentified	**8.32**	-	-	-	-	-	-	-	-	-	-	-	-	-
Unidentified	**8.86**	-	-	-	-	-	-	-	-	0.27	-	-	-	-
Unidentified	**9.41**	-	-	-	-	-	-	-	-	-	0.70	-	0.69	0.68
Unidentified	**9.53**	-	1.57	1.40	0.22	0.22	1.89	-	-	-	-	-	-	-
Unidentified	**17.34**	-	-	-	-	-	-	-	-	-	1.11	-	3.04	2.92
Unidentified	**17.59**	-	-	-	-	-	-	-	-	0.92	-	-	-	-
Unidentified	**21.77**	-	-	-	-	-	-	-	-	-	0.85	-	-	-
Unidentified	**22.71**	-	-	-	-	-	-	1.34	0.86	-	-	0.92	-	-
Unidentified	**23.37**	-	-	-	-	-	-	-	-	-	-	1.16	-	-
Unidentified	**23.97**	-	-	-	-	-	-	-	-	-	3.52	-	5.70	6.44
Unidentified	**25.04**	1.25	0.24	0.16	0.17	0.16	-	-	-	-	-	-	-	-
Unidentified	**25.72**	-	-	-	-	-	-	-	-	1.06	-	-	-	-
Unidentified	**27.08**	-	-	-	-	-	-	-	-	-	2.14	-	1.36	1.57
Unidentified	**28.64**	-	-	-	1.26	0.45	-	-	-	-	-	-	-	-
Unidentified	**28.79**	-	-	-	-	-	-	-	-	-	0.85	-	0.29	0.88
Unidentified	**29.33**	-	-	-	-	-	-	-	-	-	0.52	0.46	0.45	0.54
Unidentified	**30.08**	-	-	-	-	-	-	-	-	-	1.29	1.00	0.93	1.05
Unidentified	**30.59**	-	-	-	-	-	-	-	0.66	-	-	-	-	-
Unidentified	**31.18**	-	-	-	-	-	-	-	-	-	0.78	0.90	0.54	1.69
Unidentified	**31.53**	-	-	-	-	-	-	0.59	-	-	-	-	-	-

Suplement 5G.

Components	RT	Black poplars (*Populus nigra* L.)							Aspens (*Populus tremula* L.)					
		**PN1**	**PN2**	**PN3**	**PN4**	**PN5**	**PN6**	**PN7**	**PT1**	**PT2**	**PT3**	**PT4**	**PT5**	**PT6**
**Unidentified peaks**														
Unidentified	**31.82**	-	3.38	0.27	-	-	-	-	-	-	-	-	-	-
Unidentified	**31.82**	-	-	-	0.97	-	1.24	1.82	-	-	-	-	-	-
**Overlapped peaks**														
Mix of substances	**13.85**	-	-	-	1.24	-	0.63	0.71	-	-	-	-	-	-
Mix of substances	**15.13**	-	-	-	-	-	-	-	-	-	-	-	-	2.54
Mix of substances	**15.66**	-	-	-	5.25	-	2.41	1.79	-	-	-	-	-	-
Mix of substances	**16.46**	-	-	-	3.17	-	2.46	1.93	-	-	-	-	-	-
Mix of substances	**26.01**	-	-	-	1.91	-	2.61	2.95	-	-	-	-	-	-
Mix of substances	**26.60**	-	-	-	0.87	-	0.38	0.93	-	-	-	-	-	-
Mix of substances	**30.57**	-	-	-	0.98	-	0.32	0.80	-	-	-	-	-	-

Legend

– not detected under detection conditions

RT – experimental retention time

Suplement 6A. Composition of Polish propolis 70% ethanol in water extracts*

Component	RT	Polish propolis									
		PR-LS1	PR-LS2	PR-LS3	PR-LS4	PR-LS5	PR-LS6	PR-S1	PR-S2	PR-MR	PR-NSW
**Free phenolic acids**											
Caffeic acid	**0.83**	1.92	2.84	2.35	1.93	2.02	3.09	1.92	1.20	2.33	1.88
p-Coumaric acid	**1.23**	11.88	9.66	9.95	6.38	12.17	2.09	11.62	11.24	9.59	9.42
Ferulic acid	**1.44**	9.05	6.74	6.03	4.67	6.32	0.98	6.64	8.48	6.51	8.45
Isoferulic acid	**1.60**	0.54	1.81	1.12	1.18	0.76	2.15	0.58	0.21	1.36	0.60
3,4-Dimethyl caffeic acid (DMCA)	**3.35**	0.35	1.69	0.98	1.41	1.04	1.05	0.50	0.22	1.85	0.65
Cinnamic acid	**5.30**	0.29	0.26	0.15	0.45	1.28	0.13	0.26	0.31	0.20	0.35
β-styrylacrylic acid	**10.60**	0.31	0.37	0.38	0.30	0.38	0.77	0.36	0.17	-	0.43
**Phenolic acids glycerides**											
Acetyl-*p*-coumaroyl glycerol	**2.94**	0.19	0.19	0.13	-	-	-	0.14	0.26	-	-
**1,3-Di-*p*-coumaroylglycerol	**9.25**	1.53	0.86	0.89	0.57	0.76	0.07	0.89	1.02	1.39	0.63
p-Coumaroyl- feruloylglycerol isomer 1	**9.73**	0.37	0.20	0.23	0.18	0.22	-	0.26	0.31	0.39	0.27
***Di*- 1,3-feruloylglycerol	**10.17**	0.12	-	-	0.08	0.10	-	-	-	-	0.14
2-Acetyl-1,3-*di*-caffeoylglycerol	**10.38**	0.68	0.21	0.29	0.74	0.32	-	0.36	0.24	0.36	0.59
2-Acetyl-1-caffeoyl-3-*p*-coumaroylglycerol	**13.16**	2.55	0.92	1.08	1.70	1.01	-	1.23	1.34	1.95	1.76
2-Acetyl-3-caffeoyl-1-feruloylglycerol	**13.87**	1.37	0.62	0.75	1.05	0.77	-	0.91	0.85	1.02	1.14
**2-Acetyl-1,3-di-*p*-coumaroylglycerol	**17.52**	11.12	5.43	7.25	4.52	7.60	0.50	6.33	8.31	9.61	5.87
**2-Acetyl-3-*p*-coumaroyl-1-feruloylglycerol	**18.34**	4.44	2.84	2.99	2.53	3.00	-	3.32	3.72	3.95	3.28
**3-Acetyl-1,2-di-*p*-coumaroylglycerol	**18.62**	0.76	1.35	1.39	-	0.96	-	1.26	0.84	1.15	0.96
**2-Acetyl-1,3-di-feruloilo glycerol	**18.99**	2.33	1.62	1.50	1.88	1.73	-	1.82	2.31	1.12	2.07

Suplement 6B.

Component	RT	Polish propolis									
		PR-LS1	PR-LS2	PR-LS3	PR-LS4	PR-LS5	PR-LS6	PR-S1	PR-S2	PR-MR	PR-NSW
**Phenolic acid monoesters**											
Caffeic acid prenyl or isoprenyl ester isomer 1	**14.41**	0.38	0.90	1.16	1.57	0.56	3.11	0.74	0.20	0.64	0.48
Caffeic acid prenyl or isoprenyl ester isomer 2	**15.36**	0.27	1.36	0.73	2.33	0.22	3.47	0.39	-	0.64	0.71
Caffeic acid benzyl ester	**15.66**	-	2.58	2.64	-	-	4.63	2.41	-	3.83	-
Caffeic acid phenethyl ester (CAPE)	**18.58**	-	-	-	2.37	-	2.53	-	-	-	-
p-Coumaric acid prenyl or isoprenyl ester isomer 1	**20.17**	-	-	0.16	-	-	0.23	-	-	0.17	-
p-Coumaric acid benzyl ester	**21.08**	10.51	7.63	6.89	4.39	9.01	2.63	8.97	9.31	5.35	5.33
p-Coumaric acid prenyl or isoprenyl ester isomer 3	**21.83**	-	-	0.64	1.05	1.06	-	1.51	0.95	-	0.16
*Ferulic acid benzyl ester	**21.83**	5.19	4.56	3.69	4.09	4.44	2.72	4.79	4.64	5.35	4.18
Caffeic acid cinnamyl ester	**22.29**	0.48	1.07	1.19	2.58	1.47	2.21	0.65	2.85	0.83	2.28
p-Coumaric acid phenethyl ester	**22.88**	-	0.37	0.73	0.77	0.73	0.60	0.80	0.31	0.50	0.29
p-Coumaric acid cinnamyl ester	**25.49**	0.34	0.32	0.98	2.76	1.66	0.59	2.42	0.80	0.22	-
Metoxycinnamic acid cinnanyl ester	**31.18**	-	0.33	0.53	0.32	0.46	0.72	0.32	1.59	0.45	0.25
**Flavons**											
Apigenin	**7.26**	0.73	1.11	1.28	1.05	1.00	1.80	1.06	0.68	0.90	0.58
Chrysin 5-O-methyl ether	**7.59**	-	-	-	-	-	0.13	-	-	-	-
Luteolin 5-O-methyl ether	**9.02**	0.26	0.67	0.55	0.40	0.53	1.00	0.40	0.15	0.78	0.23
Chrysin	**14.31**	1.78	6.81	7.24	4.59	3.89	11.85	4.71	2.02	7.37	4.39
Metoxychrysn	**18.23**	-	-	0.47	0.23	-	2.39	-	-	-	-
Tectochrysin	**24.69**	-	0.50	0.79	0.50	-	1.48	0.70	-	0.43	0.26

Suplement 6C.

Component	RT	Polish propolis									
		PR-LS1	PR-LS2	PR-LS3	PR-LS4	PR-LS5	PR-LS6	PR-S1	PR-S2	PR-MR	PR-NSW
**Flavanons**											
Sakuranetin	**14.96**	1.07	1.61	0.57	0.55	0.84	-	0.56	1.01	0.57	0.43
Pinocembrin	**15.20**	2.55	4.32	5.22	5.57	4.91	6.54	5.42	3.57	4.80	3.30
Pinostrobin	**24.97**	-	0.38	0.34	0.68	-	0.51	1.28	-	-	-
**Flavonols**											
Quercetin	**4.91**	0.09	0.32	0.27	0.20	0.32	0.46	0.22	0.11	0.56	0.39
Quercetin-3-O-methylether	**6.08**	0.06	0.41	0.37	0.29	0.29	0.66	0.26	-	0.49	0.30
Kaempferol	**7.72**	0.38	1.06	0.80	0.79	1.08	1.16	0.96	0.66	1.46	0.45
Isorhamnetin	**8.31**	-	0.33	0.09	0.25	0.14	0.20	-	-	0.42	0.20
Quercetin-5,7-O-dimethylether	**9.64**	0.20	0.44	0.36	0.15	0.35	0.35	0.24	0.16	0.34	0.24
Galangin-5-O-methylether	**11.05**	0.24	0.47	0.45	0.16	0.37	0.88	0.41	-	0.37	0.34
Rhamnetin	**11.68**	0.12	0.52	0.36	0.20	0.31	0.63	0.47	0.20	0.76	0.47
Quercetin-O,O-dimethylether	**13.24**	-	0.15	0.23	0.11	-	0.75	-	0.39	0.27	0.31
Galangin	**16.21**	2.08	5.65	5.60	5.95	3.20	12.23	4.39	3.19	5.04	2.29
Kaempferid	**16.99**	0.88	1.31	0.47	1.23	0.96	0.23	0.77	0.90	0.93	-
*Galangin-3-O-methylether	**25.75**	0.19	0.31	-	0.65	-	0.71	-	-	-	-
**Flavanonols**											
Pinobanksin-5-O-methylether	**5.41**	0.32	0.77	0.98	0.48	0.44	1.88	0.59	0.26	0.78	0.53
Pinobanksin	**7.01**	0.75	1.35	1.83	1.18	0.91	3.50	1.28	0.69	1.15	0.71
Pinobansin-3-O-acetate	**17.25**	1.18	2.96	4.20	3.94	1.63	8.92	2.88	1.72	1.94	1.92

Suplement 6D.

Component	RT	Polish propolis									
		PR-LS1	PR-LS2	PR-LS3	PR-LS4	PR-LS5	PR-LS6	PR-S1	PR-S2	PR-MR	PR-NSW
**Flavanonols**											
Pinobanksin-3-O-propionate	**22.42**	-	0.29	0.42	-	0.36	0.62	0.22	0.10	0.42	0.31
Pinobanksin-3-O-butanoate or isobutanoate	**25.94**	0.52	1.06	0.95	0.83	0.52	2.24	0.72	-	0.55	0.60
Pinobanksin-3-O-pentenoate or isopentenoate isomer 1	**26.36**	-	-	-	-	-	0.16	-	-	-	-
Pinobanksin-3-O-pentanoate or isopentanoate	**29.05**	-	0.30	0.46	0.24	-	0.61	0.15	0.35	0.35	0.52
**Other**											
Vanilline	**1.34**	1.48	2.64	1.61	0.70	2.94	0.12	3.02	3.17	1.40	2.21
Benzoic acid	**2.33**	3.46	2.26	1.93	1.73	2.51	0.53	2.14	3.22	2.64	3.52
**Unidentified peaks**											
Unidentified	**0.88**	0.10	0.14	0.10	-	-	-	0.16	0.14	0.12	0.12
Unidentified	**1.06**	0.17	-	0.09	0.05	0.10	-	-	-	-	0.20
Unidentified	**1.18**	0.36	0.38	0.28	0.22	0.42	-	0.36	0.29	0.22	0.53
Unidentified	**1.71**	0.40	0.08	0.12	0.14	0.18	-	0.14	0.35	0.29	0.40
Unidentified	**1.88**	0.19	0.23	0.15	-	0.33	-	0.32	0.31	0.18	0.39
Unidentified	**1.99**	0.15	0.12	0.08	-	0.25	-	0.16	0.20	0.09	0.27
Unidentified	**2.16**	0.48	0.49	0.31	0.33	0.80	-	0.79	0.99	0.34	1.11
Unidentified	**2.37**	0.13	0.18	0.16	0.09	0.26	0.07	0.28	0.18	0.11	0.26
Unidentified	**2.72**	0.18	0.27	0.22	0.15	0.40	-	0.48	0.52	0.16	0.14
Unidentified	**3.56**	0.18	-	0.06	0.07	0.12	0.11	0.22	0.31	0.18	0.24
Unidentified	**3.71**	0.17	-	0.09	0.06	0.26	0.05	0.22	0.38	0.11	0.37

Suplement 6E.

Component	RT	Polish propolis									
		PR-LS1	PR-LS2	PR-LS3	PR-LS4	PR-LS5	PR-LS6	PR-S1	PR-S2	PR-MR	PR-NSW
**Unidentified peaks**											
Unidentified	**5.66**	0.17	0.41	0.30	0.28	0.19	0.44	0.20	-	0.21	0.24
Unidentified	**6.91**	0.07	-	-	-	-	-	-	-	-	0.11
Unidentified	**7.33**	0.83	-	-	0.18	0.37	-	0.18	0.09	0.05	0.88
Unidentified	**8.07**	2.23	0.10	0.20	0.61	0.55	-	-	-	0.12	2.19
Unidentified	**8.86**	0.14	0.08	-	0.06	0.24	-	-	-	-	-
Unidentified	**15.76**	-	0.41	0.64	-	-	1.39	-	-	-	-
Unidentified	**18.84**	0.27	0.61	-	-	-	-	-	-	-	-
Unidentified	**21.44**	-	-	-	-	-	-	-	-	-	0.22
Unidentified	**25.72**	-	-	-	-	-	0.48	-	-	-	-
Unidentified	**28.64**	1.72	1.03	1.50	0.48	0.66	0.11	1.49	2.24	1.34	2.46
Unidentified	**29.46**	-	0.10	-	-	-	-	-	-	-	0.14
Unidentified	**30.59**	-	0.13	0.21	0.80	0.24	0.52	0.18	-	-	-
Unidentified	**31.82**	-	-	-	-	-	-	-	-	0.51	-
**Overlapped peaks**											
Mix of substances	**15.66**	3.59	-	-	5.60	4.54	-	-	3.29	-	5.96
Mix of substances	**16.71**	1.66	-	0.24	2.48	1.06	-	-	3.17	-	3.38
Mix of substances	**17.77**	1.45	0.55	0.61	3.92	1.44	-	0.54	3.33	0.42	3.68

Legend

– below detection treashold

RT – experimental retention time [min.]

Suplement 7A. Composition of Canadian, German and Polish and foreign propolis 70% ethanol in water extracts*

Component	RT	Polish propolis								Foreign propolis	
		PR-NW1	PR-NW1	PR-ŚL1	PR-ŚL2	PR-SZ1	PR-SZ2	PR-SZ3	PR-SZ4	PR-GR	PR-CN
**Cinnamic acid derivates**											
Caffeic acid	**0.83**	1.55	1.57	1.48	1.30	1.79	1.61	2.00	2.06	-	0.76
p-Coumaric acid	**1.23**	8.62	8.96	8.65	8.48	6.87	8.81	12.91	9.13	0.10	6.48
Ferulic acid	**1.44**	5.34	6.80	5.98	5.65	3.83	4.84	3.97	4.75	2.93	1.97
Isoferulic acid	**1.60**	0.72	0.41	0.49	0.34	0.76	0.57	1.26	0.94	-	0.05
3,4-Dimethyl caffeic acid (DMCA)	**3.35**	1.06	0.68	0.87	0.64	0.47	0.30	0.71	0.32	3.60	-
Cinnamic acid	**5.30**	0.12	0.11	0.13	0.10	0.25	0.29	0.28	0.25	2.98	3.03
β-styrylacrylic acid	**10.60**	-	-	-	-	0.50	0.42	0.58	0.65	2.28	0.20
**Phenolic acids glycerides**											
Acetyl-p-coumaroyl glycerol	**2.94**	0.25	0.33	0.27	0.36	0.14	0.21	0.12	0.17	-	-
**1,3-Di-*p*-coumaroylglycerol	**9.25**	1.53	2.44	2.22	3.75	0.92	1.06	0.79	0.87	-	0.11
p-Coumaroyl- feruloylglycerol isomer 1	**9.73**	0.39	0.52	0.56	0.79	0.20	0.20	0.12	0.17	-	-
***Di*- 1,3-feruloylglycerol	**10.17**	-	-	0.40	0.44	-	-	0.09	-	-	-
2-Acetyl-1,3-*di*-caffeoylglycerol	**10.38**	0.50	0.71	0.45	0.54	0.18	0.23	0.09	0.06	-	-
2-Acetyl-1-caffeoyl-3-*p*-coumaroylglycerol	**13.16**	2.38	3.08	1.93	2.44	1.39	1.47	1.10	1.12	-	-
2-Acetyl-3-caffeoyl-1-feruloylglycerol	**13.87**	1.24	1.61	1.05	1.21	0.53	0.53	0.40	0.28	-	-
**2-Acetyl-1,3-di-*p*-coumaroylglycerol	**17.52**	11.41	14.95	12.16	13.34	6.52	7.32	5.54	6.00	-	0.84
**2-Acetyl-3-*p*-coumaroyl-1-feruloylglycerol	**18.34**	4.45	5.68	6.03	5.21	2.62	2.69	2.21	2.43	-	0.20
**3-Acetyl-1,2-di-*p*-coumaroylglycerol	**18.62**	0.77	0.79	1.04	0.90	1.39	1.11	1.36	1.34	-	-
**2-Acetyl-1,3-di-feruloilo glycerol	**18.99**	1.42	2.34	1.96	2.05	0.90	0.91	0.63	0.66	-	0.34

Suplement 7B.

Component	RT	Polish propolis								Foreign propolis	
		PR-NW1	PR-NW1	PR-ŚL1	PR-ŚL2	PR-SZ1	PR-SZ2	PR-SZ3	PR-SZ4	PR-GR	PR-CN
**Phenolic acid monoesters**											
Caffeic acid prenyl or isoprenyl ester isomer 1	**14.41**	0.40	0.38	0.54	0.19	0.97	0.63	1.40	1.32	1.26	-
Caffeic acid prenyl or isoprenyl ester isomer 2	**15.36**	-	-	0.27	0.23	0.99	0.68	1.52	1.68	0.54	-
Caffeic acid phenethyl ester (CAPE)	**18.58**	-	-	-	-	-	-	-	-	1.76	-
p-Coumaric acid benzyl ester	**21.08**	10.71	1.10	9.67	11.39	5.83	6.37	5.12	5.55	2.18	3.37
p-Coumaric acid prenyl or isoprenyl ester isomer 3	**21.83**	-	-	-	-	-	-	-	-	0.90	-
*Ferulic acid benzyl ester	**21.83**	5.76	5.93	5.06	5.62	2.84	3.14	2.55	2.83	-	2.20
Caffeic acid cinnamyl ester	**22.29**	1.76	2.26	1.68	1.58	5.46	4.95	3.13	2.48	1.08	3.57
p-Coumaric acid phenethyl ester	**22.88**	0.34	0.19	0.19	0.20	0.31	0.31	0.23	0.26	0.52	0.77
p-Coumaric acid cinnamyl ester	**25.49**	0.12	0.13	0.25	0.22	-	0.41	0.22	-	-	7.90
Metoxycinnamic acid cinnanyl ester	**31.18**	0.38	0.29	0.40	0.31	0.43	0.34	0.29	0.32	1.49	1.17
**Flavons**											
Metoxychrysin derivate	**3.67**	-	-	-	-	-	-	-	-	-	0.45
Apigenin	**7.26**	0.84	0.89	0.91	0.72	1.13	1.11	1.23	1.32	0.86	0.47
Luteolin 5-O-methyl ether	**9.02**	0.62	0.52	0.39	0.30	0.62	0.56	0.63	0.71	0.76	1.22
Chrysin	**14.31**	4.98	3.25	4.58	3.38	5.98	5.54	6.55	6.91	15.25	5.82
Metoxychrysin	**18.23**	-	-	-	-	0.36	0.57	0.39	0.41	2.44	1.54
Tectochrysin	**24.69**	-	-	-	-	-	-	-	-	6.29	-
**Flavanons**											
Sakuranetin	**14.96**	0.60	0.86	0.58	0.68	1.14	0.90	0.50	0.67	-	-
Pinocembrin	**15.20**	4.50	3.67	3.83	3.54	4.79	4.59	4.77	4.70	8.64	9.13

Suplement 7C.

Component	RT	Polish propolis								Foreign propolis	
		PR-NW1	PR-NW1	PR-ŚL1	PR-ŚL2	PR-SZ1	PR-SZ2	PR-SZ3	PR-SZ4	PR-GR	PR-CN
**Flavanon**											
Pinostrobin	**24.97**	-	-	-	-	0.58	0.24	0.50	-	7.30	-
**Flavonole**											
Quercetin	**4.91**	0.39	0.23	0.27	0.26	0.24	0.24	0.26	0.28	0.20	0.31
Quercetin-3-O-methyl ether	**6.08**	0.37	0.23	0.25	0.20	0.24	0.20	0.23	0.27	0.64	0.93
Kaempferol	**7.72**	1.00	0.78	0.71	0.62	0.92	0.92	0.92	0.98	0.80	1.08
Isorhamnetin	**8.31**	0.33	0.26	0.19	0.21	0.25	0.20	0.16	0.29	0.21	0.66
Quercetin-5,7-O,O-dimethyl ether	**9.64**	0.30	0.30	0.19	0.19	0.31	0.24	0.22	0.30	1.05	1.31
Galangin-5-O-methyl ether	**11.05**	0.22	0.26	0.34	0.31	0.35	0.35	0.41	0.45	1.87	1.23
Rhamnetin	**11.68**	0.52	0.22	-	0.57	0.49	0.61	0.41	0.54	1.19	0.54
Quercetin-O,O-dimethyl ether	**13.24**	-	-	-	-	-	-	-	-	1.57	-
Galangin	**16.21**	3.38	2.34	3.05	2.30	5.92	6.10	7.10	7.13	10.59	6.08
Kaempferid	**16.99**	0.51	0.96	0.66	0.77	0.47	0.94	0.28	0.47	-	-
*Galangin-3-O-methyl ether	**25.75**	-	-	-	-	-	-	-	-	2.60	-
**Flavanonols**											
Pinobanksin-5-O-methyl ether	**5.41**	0.51	0.33	0.45	0.32	0.85	0.79	1.02	1.08	-	3.76
Pinobanksin	**7.01**	0.90	0.94	1.08	0.83	1.66	1.57	2.02	2.10	1.81	2.42
Pinobanksin-3-O-acetate	**17.25**	1.24	1.15	1.82	1.21	3.92	3.43	4.37	4.93	4.04	8.73
Pinobanksin-3-O-propionate	**22.42**	0.32	0.29	0.43	0.32	0.25	0.18	0.14	0.19	2.17	0.53
Pinobanksin-3-O-butanoate or isobutanoate	**25.94**	0.43	0.23	0.34	0.22	0.58	0.50	0.74	0.68	2.53	1.19

Suplement 7D.

Component	RT	Polish propolis								Foreign propolis	
		PR-NW1	PR-NW1	PR-ŚL1	PR-ŚL2	PR-SZ1	PR-SZ2	PR-SZ3	PR-SZ4	PR-GR	PR-CN
**Flavanonols**											
Pinobanksin-3-O-pentanoate or isopentanoate	**29.05**	1.37	0.38	0.38	0.44	0.49	0.39	0.36	0.40	-	-
**Other**											
Vanillin	**1.34**	1.68	2.53	2.45	2.57	1.76	1.60	1.14	1.37	-	0.84
Benzoic acid	**2.33**	2.06	2.72	1.96	1.75	1.98	2.52	2.29	2.82	0.31	3.44
**Unidentified peaks**											
Unidentified	**0.88**	0.07	0.13	0.13	0.14	0.13	0.12	0.11	0.11	-	-
Unidentified	**1.06**	0.07	0.10	-	-	0.06	0.05	0.07	0.11	-	-
Unidentified	**1.18**	0.24	0.25	0.29	0.27	0.22	0.18	0.13	0.19	-	-
Unidentified	**1.71**	0.14	0.26	0.11	0.18	0.15	0.14	0.18	0.19	-	0.09
Unidentified	**1.88**	0.28	0.40	0.34	0.37	0.36	0.32	0.24	0.29	-	0.05
Unidentified	**1.99**	0.13	0.19	0.15	0.13	0.18	0.15	0.11	0.11	-	0.06
Unidentified	**2.16**	0.55	0.76	0.63	0.59	0.62	0.57	0.37	0.43	-	0.13
Unidentified	**2.37**	0.11	0.16	0.14	0.13	0.18	0.12	0.11	0.12	-	-
Unidentified	**2.72**	0.33	0.52	0.35	0.36	0.32	0.26	0.24	0.23	-	-
Unidentified	**3.56**	0.13	0.29	0.18	0.21	0.22	0.20	0.13	0.19	-	-
Unidentified	**3.71**	0.18	0.35	0.23	0.27	0.43	0.33	0.24	0.29	-	-
Unidentified	**4.18**	-	-	-	-	-	-	-	-	0.72	-
Unidentified	**5.05**	-	-	-	-	-	-	-	-	0.94	-

Suplement 7E.

Component	RT	Polish propolis								Foreign propolis	
		PR-NW1	PR-NW1	PR-ŚL1	PR-ŚL2	PR-SZ1	PR-SZ2	PR-SZ3	PR-SZ4	PR-GR	PR-CN
**Unidentified peaks**											
Unidentified	**5.61**	-	-	-	-	-	-	-	-	-	0.15
Unidentified	**5.66**	0.14	0.10	0.10	0.12	0.19	0.15	0.19	0.17	0.58	-
Unidentified	**7.33**	0.24	0.47	0.14	-	-	-	0.28	0.29	-	0.35
Unidentified	**8.07**	0.37	0.90	0.11	-	0.25	-	0.47	0.53	-	1.29
Unidentified	**8.32**	-	-	-	-	-	-	-	-	0.40	1.16
Unidentified	**8.86**	-	-	0.14	-	-	0.14	-	-	-	-
Unidentified	**13.13**	-	-	-	-	-	-	-	-	-	0.26
Unidentified	**13.58**	-	-	-	-	-	-	-	-	-	0.80
Unidentified	**15.76**	-	-	-	-	0.93	1.20	0.97	0.73	-	-
Unidentified	**18.84**	-	-	-	-	0.51	0.43	0.16	0.30	-	-
Unidentified	**19.20**	-	-	-	-	-	-	-	-	-	0.24
Unidentified	**21.30**	-	-	-	-	-	-	-	-	-	0.44
Unidentified	**21.44**	-	-	-	-	1.18	0.63	0.39	0.26	-	-
Unidentified	**22.71**	-	-	-	-	-	-	-	-	-	0.07
Unidentified	**23.24**	-	-	-	-	-	-	-	-	-	0.36
Unidentified	**24.60**	-	-	-	-	-	-	-	-	-	0.44
Unidentified	**26.41**	-	-	-	-	-	-	-	-	-	0.20
Unidentified	**27.25**	-	-	-	-	-	-	-	-	-	0.20
Unidentified	**28.41**	-	-	-	-	-	-	-	-	-	0.24

Suplement 7F.

Component	RT	Polish propolis								Foreign propolis	
		PR-NW1	PR-NW1	PR-ŚL1	PR-ŚL2	PR-SZ1	PR-SZ2	PR-SZ3	PR-SZ4	PR-GR	PR-CN
**Unidentified peaks**											
Unidentified	**28.64**	0.30	1.57	1.93	2.19	1.45	1.53	1.31	2.32	-	-
Unidentified	**29.88**	-	-	-	-	-	-	-	-	-	0.34
Unidentified	**31.36**	-	-	-	-	-	-	-	-	-	2.08
Unidentified	**31.53**	-	-	-	-	-	-	-	-	-	0.32
Unidentified	**31.82**	-	-	-	-	-	-	-	-	2.62	0.28
**Overlapped**											
Mix of substances	**15.66**	5.09	4.76	2.95	3.58	5.54	4.50	5.08	4.96	-	2.95
Mix of substances	**16.71**	2.03	2.01	1.31	1.24	3.29	2.90	2.52	2.30	-	2.19
Mix of substances	**17.77**	1.33	2.18	1.37	1.21	2.45	2.34	1.37	1.20	-	0.60

Legend

– under detection treshold

RT – experimental retention time [min.]

Suplement 8. Full descritpion of samples

Code	Collecting time	Type of sample	Place of collect
PN1	February 2013	*P. nigra* L. buds	Szczodre, forest
PN2	March 2015	*P. nigra* L. buds	Szczodre, forest
PN3	March 2015	*P. nigra* L. buds	Kórnik, Arboretum
PN4	March 2015	*P. nigra* L. buds	Kórnik, Arboretum
PN5	January 2016	*P. nigra* L. buds	Wrocław
PN6	2016	*P. nigra* L. buds	commercial sample
PN7	2016	*P. nigra* L. buds	commercial sample
PT1	February 2013	*P. tremula* L. buds	Szczodre, forest
PT2	February 2015	*P. tremula* L.	Szczodre, forest
PT3	February 2015	*P. tremula* L. buds	Szczodre, forest
PT4	March 2016	*P. tremula* L. buds	Szczodre, forest
PT5	March 2016	*P. tremula* L. buds	Szczodre, forest
PT6	March 2016	*P. tremula* L. buds	Wojsławice, Arboretum
PR-LS1	2015	propolis	Down Silesia, (traveling apiary)
PR- LS2	2015	propolis	Down Silesia, (traveling apiary)
PR- LS3	2015	propolis	Down Silesia, (traveling apiary)
PR- LS4	2015	propolis	Down Silesia (Jedlina), stationary apiary
PR- LS5	2015	propolis	Down Silesia, (traveling apiary)
PR- LS6	2015	propolis	Down Silesia, (traveling apiary)
PR-MR	2013	propolis	Down Silesia, (Marszowice), stationary apiary
PR-NSW	2012	propolis	Down Silesia (Nowa Wieś, Nieciszów, Śliwice), mixed sample
PR-S1	2016	propolis	Podkarpacie, (Bircza), pasieka stojąca
PR-S2	2016	propolis	Podkarpacie, stationary apiary
PR-NW1	August 2013	propolis	Nowa Wieś, stationary apiary
PR-NW2	September 2013	propolis	Nowa Wieś, stationary apiary
PR-ŚL1	July 2013	propolis	Śliwice, stationary apiary
PR-ŚL2	August 2013	propolis	Śliwice, stationary apiary
PR-SZ1	May 2013	propolis	West Pomerania (Szczecin), stationary apiary
PR-SZ2	June 2013	propolis	West Pomerania (Szczecin), stationary apiary
PR-SZ3	July 2013	propolis	West Pomerania (Szczecin), stationary apiary
PR-SZ4	September 2013	propolis	West Pomerania (Szczecin), stationary apiary
PR-PR-CN	August 2015	propolis	Canada (Ontario), stationary apiary
PR-PR-GR	2014	propolis	Germany, commercial sample
